# Selection of Filtering and Image Texture Analysis in the Radiographic Images Processing of Horses’ Incisor Teeth Affected by the EOTRH Syndrome

**DOI:** 10.3390/s22082920

**Published:** 2022-04-11

**Authors:** Kamil Górski, Marta Borowska, Elżbieta Stefanik, Izabela Polkowska, Bernard Turek, Andrzej Bereznowski, Małgorzata Domino

**Affiliations:** 1 Department of Large Animal Diseases and Clinic, Institute of Veterinary Medicine, Warsaw University of Life Sciences, 02-787 Warsaw, Poland; kamil_gorski@sggw.edu.pl (K.G.); elzbieta_stefanik@sggw.edu.pl (E.S.); bernard_turek@sggw.edu.pl (B.T.); 2 Institute of Biomedical Engineering, Faculty of Mechanical Engineering, Białystok University of Technology, 15-351 Bialystok, Poland; m.borowska@pb.edu.pl; 3 Department and Clinic of Animal Surgery, Faculty of Veterinary Medicine, University of Life Sciences in Lublin, 20-950 Lublin, Poland; izabela.polkowska@up.lublin.pl; 4 Department of Veterinary Epidemiology and Economics, Faculty of Veterinary Medicine, Warsaw University of Life Sciences, 02-787 Warsaw, Poland; andrzej_bereznowski@sggw.edu.pl

**Keywords:** equine odontoclastic tooth resorption and hypercementosis, filtering, texture analysis, digital image processing, dental care

## Abstract

Equine odontoclastic tooth resorption and hypercementosis (EOTRH) is one of the horses’ dental diseases, mainly affecting the incisor teeth. An increase in the incidence of aged horses and a painful progressive course of the disease create the need for improved early diagnosis. Besides clinical findings, EOTRH recognition is based on the typical radiographic findings, including levels of dental resorption and hypercementosis. This study aimed to introduce digital processing methods to equine dental radiographic images and identify texture features changing with disease progression. The radiographs of maxillary incisor teeth from 80 horses were obtained. Each incisor was annotated by separate masks and clinically classified as 0, 1, 2, or 3 EOTRH degrees. Images were filtered by *Mean*, *Median*, *Normalize*, *Bilateral*, *Binomial*, *CurvatureFlow*, *LaplacianSharpening*, *DiscreteGaussian*, and *SmoothingRecursiveGaussian* filters independently, and 93 features of image texture were extracted using *First Order Statistics* (FOS), *Gray Level Co-occurrence Matrix* (GLCM), *Neighbouring Gray Tone Difference Matrix* (NGTDM), *Gray Level Dependence Matrix* (GLDM), *Gray Level Run Length Matrix* (GLRLM), and *Gray Level Size Zone Matrix* (GLSZM) approaches. The most informative processing was selected. GLCM and GLRLM return the most favorable features for the quantitative evaluation of radiographic signs of the EOTRH syndrome, which may be supported by filtering by filters improving the edge delimitation.

## 1. Introduction

Dental diseases are known to significantly affect horses’ health [1,2,3,4,5,6], affecting not only the body condition [1] but also the functioning of the whole body [2] and predisposing horses to life-threatening colic [5,6]. Dental diseases are the third most common problem in equine veterinary medicine [7,8]. It is estimated that 24% of young horses, even without clinical signs of oral disease, have oral abnormalities [9]. In Dixon and Tremaine’s study [7], 11% of the examined horses showed disorders in the area of the incisor teeth. In Wilson and Liyou’s study [10], 20% of the examined horses had abnormalities in their incisor teeth. In the Maslauskas et al. study [11], 26% of the Lithuanian dray horses demonstrated signs of incisor tooth disorders. Moreover, the teeth pathology concerned more often the incisor teeth of the maxilla than the mandible, and more than one tooth [7,8]. Since incisor teeth, unlike the cheek teeth, are easier to visually assess due to the rostral position [8], any abnormalities can be diagnosed and corrected at an early stage [11]. However, just in the early stages of the disease, a detailed dental examination, including radiographic imaging, is necessary to make the correct diagnosis [12,13] and to plan and choose the treatment [14], as most of the early changes concern the alveolar part of the teeth [12,13].

The Equine odontoclastic tooth resorption and hypercementosis (EOTRH) syndrome is one of the important diseases affecting the incisor teeth [12,15,16,17,18], whose etiopathogenesis remains not fully understood. The EOTRH syndrome may be diagnosed based on the anamnestic data and careful clinical examination; however, only the radiological signs are determinant, often exclusive and usually conclusive [19]. Therefore, it is widely accepted that the careful evaluation of the radiological images of the incisor teeth provides valuable data on the advancement of the disease [13]. Moreover, the changes in the radiographic image of the incisal processes appear earlier than the clinical symptoms [13,20,21]. As teeth demonstrate high radiodensity, they are extremely radiopaque and radiographically well-defined [19]. Therefore, their radiological images are visually inspected for the presence of signs of tooth resorption and bulbous enlargement of the intra-alveolar part of the teeth [13,18]. Moreover, the radiological images reveal the signs of widening of the periodontal ligament space, root resorption, erosion of the apical part of the root, root and reserve crown enlargement due to hypercementosis, irregular and/or rough surface of an intra-alveolar part, disruption of the lamina dura, osteomyelitis, atrophy or other pathology in the course of a pulp canal, or fractures of the root and the reserve crown [18]. In general, these symptoms are related to the following two ongoing processes of varying intensity: resorption and hypercementosis, which are involved in the pathogenesis of EOTRH. As resorption is sometimes present without hypercementosis, whereas hypercementosis is never present alone, hypercementosis seems to be a reparative or secondary process [22]. Therefore, the pathological features of both processes were included in the radiological classification system designed specifically for the detection of signs of EOTRH [23]. The visual classification system, introduced by Hüls et al. [23], uses radiological criteria (shape, contour, radiodensity, and delineation of the periodontal space) as well as macroscopical criteria (shape, surface structure, contour, and consistency) to classify EOTRH affected teeth as mild, moderate, or severely altered [13]. A more detailed clinical classification of the incisor tooth resorption and hypercementosis processes in horses was introduced by Henry et al. [22] based on the humans’ and canines’ classification methods. Most of the previous studies focus on the description of the disease, whereas the problem arising from the difficulty of EOTRH diagnosis, especially at an early stage, remains unsolved.

Therefore, the statistical decryptions of the radiographic digital image are proposed here, as the large field of medical applications [24,25] and good results for images where the textures are visually easily separable [26], were reported. The first- and second-order descriptive statistics have successfully been used in human medicine to improve the extraction of texture features of ultrasound images [27], thermal images [28], magnetic resonance images [29,30], computed tomography images [31], and radiographic images [32,33]. In equine medicine, they have been recently applied to the detailed characteristics of the thermal images reflected the increase in the metabolic activity in response to the pregnancy [34] as well as single [35] or multiple [36] exercise. The approaches to feature extraction from digital images represent seven major classes: statistical approaches, structural approaches, transform-based approaches, model-based approaches, graph-based approaches, learning-based approaches, and entropy-based approaches [37,38]. The statistical approach enables the description of the image by a set of statistical quantitative features such as intensity histogram-based and texture matrix-based features. The matrix-based features are subdivided into the following classes: Gray Level Co-occurrence Matrix (GLCM), Neighbouring Gray Tone Difference Matrix (NGTDM), Gray Level Dependence Matrix (GLDM), Gray Level Run Length Matrix (GLRLM), and Gray Level Size Zone Matrix (GLSZM) [37], whereas intensity histogram-based features are represented by First Order Statistics (FOS) [37]. When the matrices resulting from the texture analysis are calculated for each direction vector, the average values are returned, and the texture matrices become invariant to rotation and translation [37]. Such matrices can be useful in biomedical applications and their features can be calculated based on original images or filtered images [39,40].

Different approaches of the image feature extraction and image processing steps can affect quantified characteristics of the image [38]. One may observe that the image features can be calculated based on original images or filtered images. However, it should be kept in mind that the values of texture features received after different filtering will not be the same. Therefore, the filtrating algorithms are concerned in terms of a specific objective [38]. For example, in human dentistry, digital radiographic images are routinely filtered and used to enhance brightness, contrast, and edges, carrying the potential for increasing their diagnostic value [41,42]. Minimal filtering, the most commonly used in this type of analysis, was applied to remove outliers (*Median* filter), increase contrast (*Normalization* filter), preserve edges (*Bilateral* filter), highlight regions of rapid intensity change (*Laplacian* filter), and gently smear to remove noise (*DiscreteGaussian* and *Smoothingrecursive Gaussian* filters) [41,42]. In equine dentistry, a similar image filtering has not yet been applied, whereas the processes of tooth resorption and hypercementosis are similar in humans and horses. Moreover, the comparison between different radiological methods for assessment of tooth root resorption [19] and statistical analysis of radiographic textures illustrating teeth [32] was performed in humans, but not in horses. Therefore, we hypothesized the effect of resorption and hypercementosis processes on the appearance of a tooth radiograph may be similar, thus human approaches can be successfully applied to veterinary medicine. However, both image filtering methods and texture analysis approaches have to be tested on the equine specimen in order to demonstrate the effect of digital image processing on the basic features quantification. To the best of our knowledge, this is the first report on equine tooth texture analysis assessing its usefulness in diagnosing equine-specific dental diseases [12,13].

Based on the presented background, we hypothesize that the radiographic signs of the EOTRH syndrome could be successively quantified using the statistical decryptions of the radiographic digital image. Therefore, this study aimed to examine nine filtering algorithms and six texture analysis approaches to indicate the features of the descriptive statistics that change with the EOTRH degree. The identified features are preliminary to introducing digital image processing into the quantification of radiographic signs of the EOTRH syndrome and will be used in future research investigating the application of texture analysis in equine dental veterinary medicine.

## 2. Materials and Methods

### 2.1. Horses

The study was carried out on eighty privately owned horses (*n* = 80) (age mean ± SD: 16.9 ± 7.0; 37 geldings, 43 mares), presented to a dental veterinarian by their owner and underwent a routine dental examination from July 2021 to December 2021. The horses represented predominantly warmblood breeds (*n* = 76) including mostly three Polish warmblood breeds (*n* = 41) in it: Polish Halfbred horse (*n* = 30), Wielkopolska (*n* = 8), and Malopolska (*n* = 3) breeds. Moreover, Arabian horses (*n* = 13), Schlesisches Warmblood horses (*n* = 10), Dutch Warmblood (*n* = 7), Thoroughbred horses (*n* = 5), and Polish coldblooded horses (*n* = 4) were also included. The experimental protocol was approved by the II Local Ethical Committee on Animal Testing in Warsaw on behalf of the National Ethical Committees on Animal Testing (No WAW2/091/2020 approved on 29 July 2020).

### 2.2. Image Collection

Horses were sedated with detomidine, xylazine, or a combination of both, with some cases given additional butorphanol was administered intravenously. The dose and composition of the sedation were determined on the basis of the horse’s body weight and temperament. The Haussmann’s halter retractor was used to open the oral cavity for visual examination and digital palpation. Then, the oral cavity was rinsed with 400 mL of water to remove the remainder of the food. The detailed dental examination was conducted following the previously used standard protocol. The results of the oral cavity examination were documented using an equine dental chart [18]. The intraoral radiographic image was obtained using a bisecting angle technique and the dorsoventral projection for the maxillary teeth [18,20,22], following the standard, previously described protocol and under the same settings of the x-ray tube [18]. 

Based on the dental examination including the radiological signs, each maxillary incisor tooth was classified as normal (0), mild (1), moderate (2), and severe (3) EOTRH affected. The visual classification system, introduced by Hüls et al. [23] with Rehrl et al. modification [13] was used. The radiological criteria included shape, contour, radiodensity and delineation of the periodontal space. The macroscopical criteria included shape, surface structure, contour, and consistency. Based on the findings obtained, the incisor teeth were assigned to the normal group (EOTRH 0; *n* = 105), the mild EOTRH group (EOTRH 1; *n* = 195), the moderate EOTRH group (EOTRH 2; *n* = 111), or the severe EOTRH group (EOTRH 3; *n* = 61). The total number of incisors for all groups was 472; therefore, 8 incisors were excluded due to tooth fractures.

### 2.3. Image Processing

**Figure 1:**
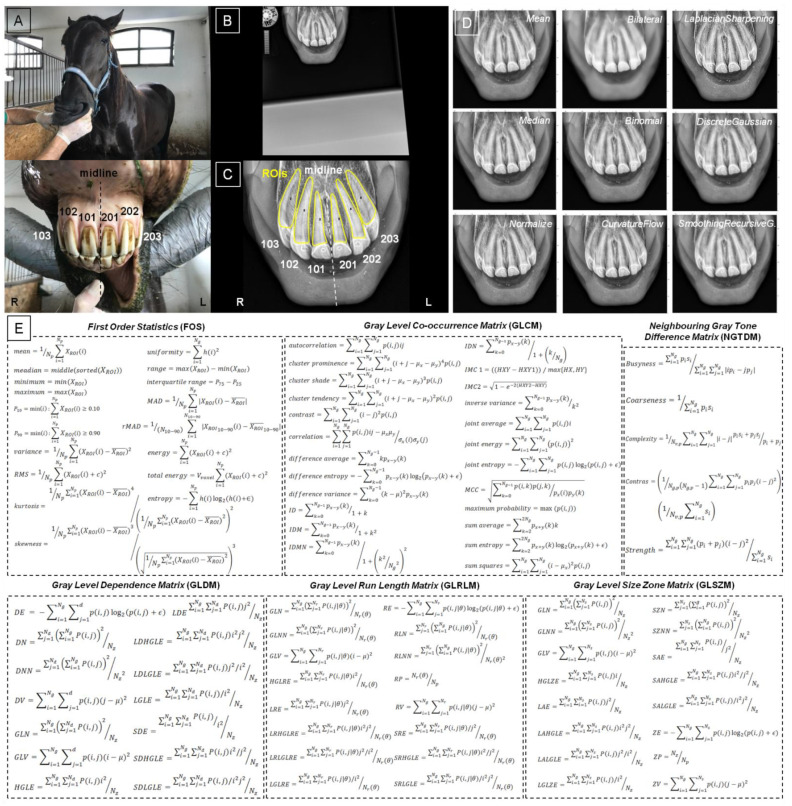
Processing steps for radiographic image texture analysis. Clinical examination (**A**); radiographic image acquisition (**B**); masks annotation and image segmentation with regions of interest (ROIs) marked with yellow lines (**C**); input image filtering (**D**); image texture features extraction from output filtered images (**E**).

Processing steps for radiographic image texture analysis included (i) image acquisition; (ii) masks annotation and image segmentation; (iii) input image filtering using nine filtering algorithms: *Mean*, *Median*, *Normalize*, *Bilateral*, *Binomial*, *CurvatureFlow*, *LaplacianSharpening*, *DiscreteGaussian*, and *SmoothingRecursiveGaussian;* (iv) image texture features extraction from output filtered images using six analytical approaches: *First Order Statistics* (FOS), *Gray Level Co-occurrence Matrix* (GLCM), *Neighbouring Gray Tone Difference Matrix* (NGTDM), *Gray Level Dependence Matrix* (GLDM), *Gray Level Run Length Matrix* (GLRLM), and *Gray Level Size Zone Matrix* (GLSZM) (Figure 1). The image texture analysis, including steps iii and iv, was applied to regions of interest (ROIs) annotated by the masks during image segmentation in step ii. Each ROI was considered separately.

#### 2.3.1. Masks Annotation and Image Segmentation

The masks representing 6 maxillary incisor teeth were manually annotated. The modified Triadan system for equine dental nomenclature was used [43]. In the maxilla, the quadrant was identified with 1 on the right and 2 on the left, and the incisor teeth were numbered consecutively, beginning with 01 at the midline and proceeding distally. Thus, 103, 102, 101, 201, 202 and 203 incisor teeth were annotated separately (Figure 1C). The masks segmented each image into six ROIs representing the consecutive incisor teeth. The masks were annotated from a high radiodensity line representing the edge of the rubbing surface of the incisor toot along the lateral and medial surfaces of the tooth to the tooth root. The masks were individually fitted to the separate teeth and did not include lips, mucous membranes, and the bone of the incisal processes. The masks were annotated using the ImageJ software (version 1.46r, Wayne Rasband, National Institutes of Health, USA).

#### 2.3.2. Filtering

**Figure 2:**
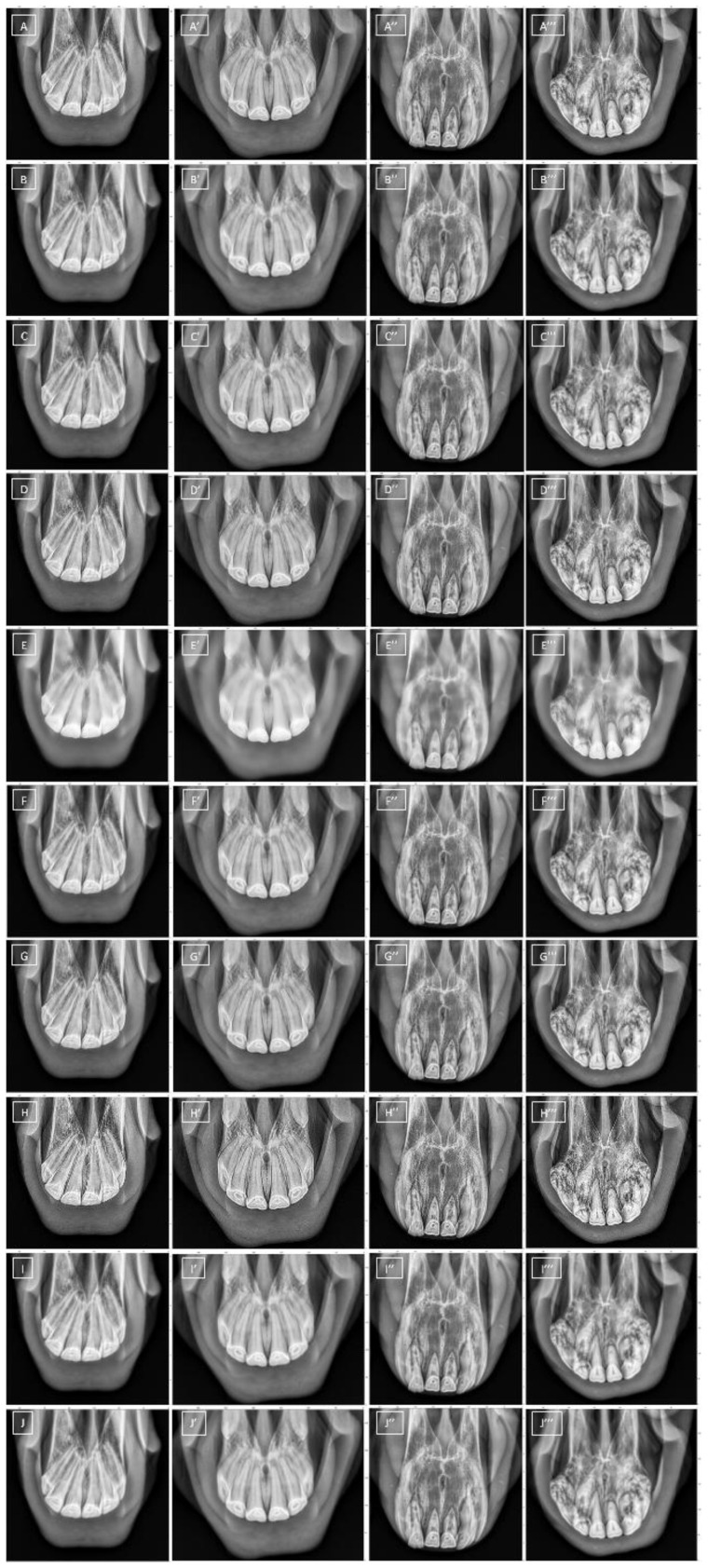
The samples of radiographic images representing normal maxillary incisor teeth (EOTRH 0, **A**–**F**) and mild (EOTRH 1, **A’**–**F’**), moderate (EOTRH 2, **A’’**–**F’’**), and severe (EOTRH 3, **A’’’**–**F’’’**) Equine odontoclastic tooth resorption and hypercementosis (EOTRH) syndrome. The input images (**A**–**A’’’**) and output images filtered by *Mean* (**B**–**B’’’**), *Median* (**C**–**C’’’**), *Normalize* (**D**–**D’’’**), *Bilateral* (**E**–**E’’’**), *Binomial* (**F**–**F’’’**), *CurvatureFlow* (**G**–**G’’’**), *LaplacianSharpening* (**H**–**H’’’**), *DiscreteGaussian* (**I**–**I’’’**), and *SmoothingRecursiveGaussian* (**J**–**J’’’**) filters, respectively.

The following nine different filtering algorithms were used to reduce the noise in the radiographic images (Figure 2). The filtering algorithms were implemented in SimpleITK toolkit in Python language [39,40,44].

(*i*) *Mean filter* is a linear filter. The pixels of the output image are the average values of the pixels in the neighborhood of the input pixel being calculated. The parameter of this filter is the window containing the neighborhood of the calculated pixel (w = 3) [40,45].
(*ii*) *Median filter* is a non-linear filter. The pixels of the output image are the medians of the pixels in the neighborhood of the input pixel being calculated. This filter requires a neighborhood size (w = 3) [45].
(*iii*) *Normalize filter* is a linear filter, when the normalizing image involves setting its mean to zero and its variance to one. The output image is a rescaled image in which the pixels have zero mean and unit variance [45].
(*iv*) *Bilateral filter* is a non-linear filter that consists of a domain filter and a range filter. The output image contains the new pixel value calculated based on the pixels similar to a pixel in the image domain and similar to a pixel in the image range. Two Gaussian kernels are used in both image domain and image range [46]. In these filter settings, the sigma in the image domain was 4.0 and the sigma in the image range was 50.
(*v*) *Binomial filter* is a linear filter, which separable blur on each image dimension. The output image is closer to performing a spline operation with a Gaussian window, after n iterations calculating the average of the nearest neighbors along each direction [47].
(*vi*) *CurvatureFlow filter* is a linear filter that reduces the noise using curvature-based flow. The output image is displayed as a set of brightness levels calculated using curvature-based speed function [48]. In these filter settings, the number of update iterations to perform was 5 and the interval between each update was 0.05.
(*vii*) *LaplacianSharpening filter* is a non-linear filter, that sharpens an image using a Laplacian. The output image is produced after a pixel convolution with a Laplacian operator, which results in change of the regions of rapid intensity and highlights the edges [49].
(*viii*) *DiscreteGaussian filter* is a linear filter that calculates derivatives using discrete Gaussian derivative operator (kernel). The output image contains a disjoint spline of the input image with a kernel where the variance and standard deviation (sigma) are evaluated as physical units [50].
(*ix*) *SmoothingRecursiveGaussian filter* is a linear that uses Gaussian kernels implemented as IIR filter. The output image is produced after convolution of the input image with Gaussian kernels. In these filter settings, the sigma parameter was 3 [51].

#### 2.3.3. Extraction of Image Texture Features

The following six texture analysis approaches [37] were used to extract 93 texture features of the radiographic image from each of the six segmented ROIs, separately (Figure 1E). Texture features were calculated independently for individual filtrated output images using PyRadiomics, an open-source python package for the extraction of features from radiographic images [52].

(*i*) *First Order Statistics* (FOS) describe the distribution of pixel intensity using the first-order histogram statistics of images. FOS returns the following 18 features: mean, median, minimum, maximum, 10th percentile, 90th percentile, variance, root mean squared (RMS), kurtosis, skewness, uniformity, range, interquartile range, mean absolute deviation (MAD), robust mean absolute deviation (rMAD), energy, total energy, and entropy [37].
(*ii*) *Gray Level Co-occurence Matrix* (GLCM) describes the second-order joint probability function of the image defined as P(*i,j*). Each element of this matrix represents the mutual spatial relationship between pairs of pixels with specific intensity levels in different directions along angle θ and at different distances δ of pixel pairs [53]. GLCM returns the following 24 features: autocorrelation, cluster prominence, cluster shade, cluster tendency, contrast, correlation, difference average, difference entropy, difference variance, inverse difference (ID), inverse difference moment (IDM), inverse difference moment normalized (IDMN), inverse difference normalized (IDN), informational measure of correlation 1 (IMC 1), informational measure of correlation 2 (IMC 2), inverse variance, joint average, joint energy, joint entropy, maximal correlation coefficient (MCC), maximum probability, sum average, sum entropy, and sum of squares [53].
(*iii*) *Neighbouring Gray Tone Difference Matrix* (NGTDM) describes the difference between a gray value and the average gray value of its neighbors within a Chebyshew distance δ using the second-order statistic. Matrix contains the sum of the absolute differences in gray level [54]. NGTDM returns the following 5 features: busyness, coarseness, complexity, contrast, and strength [54].
(*iv*) *Gray Level Dependence Matrix* (GLDM) describes dependencies of gray level in the image using the second-order statistic. A gray level dependency is defined as the number of connected pixels within distance δ, which are dependent on the center pixel. Each element of the gray level dependence matrix P(*i,j*) describes the number of times appearance a pixel with gray level *i* with *j* dependent pixels in its neighbourhood [55]. GLDM returns the following 14 features: dependence entropy (DE), dependence non-uniformity (DN), dependence non-uniformity normalized (DNN), dependence variance (DV), gray level non-uniformity (GLN), gray level variance (GLV), high gray level emphasis (HGLE), large dependence emphasis (LDE), large dependence high gray level emphasis (LDHGLE), large dependence low gray level emphasis (LDLGLE), low gray level emphasis (LGLE), small dependence emphasis (SDE), small dependence high gray level emphasis (SDHGLE), and small dependence low gray level emphasis (SDLGLE) [55].
(*v*) *Gray Level Run Length Matrix* (GLRLM) describes gray level runs in the image using the second-order statistic. A gray level run is defined as the length of the number of consecutive pixels with the same gray level values. Each element in the gray level run length matrix P(*i,j*) describes the number of runs with the gray level *i* and the length *j* in different directions along angle θ [56,57]. GLRLM returns the following 16 features: gray level non-uniformity (GLN), gray level non-uniformity normalized (GLNN), gray level variance (GLV), high gray level run emphasis (HGLRE), long run emphasis (LRE), long run high gray level emphasis (LRHGLE), long run low gray level emphasis (LRLGLE), low gray level run emphasis (LGLRE), run entropy (RE), run length non-uniformity (RLN), run length non-uniformity normalized (RLNN), run percentage (RP), run variance (RV), short run emphasis (SRE), short run high gray level emphasis (SRHGLE), and short run low gray level emphasis (SRLGLE) [56,57].
(*vi*) *Gray Level Size Zone Matrix* (GLSZM) describes gray-level zones in the image using the second-order statistic. A gray-level zone is defined as the number of connected pixels with the same gray-level intensity. Each element in the gray level size zone matrix P(*i,j*) describes the number of zones with gray level *i* and size *j* [58]. GLSZM returns the following 16 features: gray level non-uniformity (GLN), gray level non-uniformity normalized (GLNN), gray level variance (GLV), high gray level zone emphasis (HGLZE), large area emphasis (LAE), large area high gray level emphasis (LAHGLE), large area low gray level emphasis (LALGLE), low gray level zone emphasis (LGLZE), size-zone non-uniformity (SZN), size-zone non-uniformity normalized (SZNN), small area emphasis (SAE), small area high gray level emphasis (SAHGLE), small area low gray level emphasis (SALGLE), zone entropy (ZE), zone percentage (ZP), and zone variance (ZV) [58].

### 2.4. Statistical Analysis

Statistical analysis was performed using GraphPad Prism software, version 6, (GraphPad Software Inc., San Diego, CA, USA). Data were presented as data series for each filtering independently, where each incisor tooth represented one realization. The numerical data in Supplementary Tables S1–S54 were presented as mean ± standard deviation (SD). Data series were tested independently for univariate distributions using a Shapiro–Wilk normality test. Data analysis was performed in the following three steps: (i) testing the differences between data series of the EOTRH classes; (ii) testing the increase or decrease with the EOTRH; (iii) calculating the coefficient of variation for the consecutive features in each of each texture analysis approach including all filters used.

The comparisons between (i) data series representing EOTRH classes were assessed using the ordinary one-way ANOVA for Gaussian data and the Kruskal–Wallis for non-Gaussian data. The alpha value was established as α = 0.05. On the corresponding summarizing plot, when a feature differed between EOTRH classes the cell was marked with gray and the number of features that differed between classes was given in each row. Statistical analysis was performed using GraphPad Prism6 software (GraphPad Software Inc., San Diego, CA, USA).

The comparisons between (ii) the mean rank of each EOTRH class with the mean rank of every other EOTRH class were assessed using the Ordinary one-way ANOVA followed by Tukey’s multiple comparisons test for Gaussian data and the Kruskal–Wallis test followed by the Dunn’s multiple comparisons test for non-Gaussian data. The alpha value was established as α = 0.05. On the corresponding summarizing plot, when a feature was found to significantly increase or decrease with the degree of the EOTRH, the cell was marked with red or blue, respectively. The number in the cell indicates the degree of EOTRH from which the increase or decrease in the values of the features begins. The selected filtering and texture analysis approaches were presented on scatter plots with bars using mean ± SD and dots representing each realization. Statistical analysis was performed using GraphPad Prism6 software (GraphPad Software Inc., San Diego, CA, USA).

The coefficient of variation (iii) was calculated for the consecutive features of FOS, GLCM, NGTDM, GLDM, GLRLM, and GLSZM using data set containing all filtrated output images. The coefficient of variation was presented on plots independently for consecutive texture analysis approaches, where the red dashed line indicates the mean value of the feature for the total data set of consecutive texture analysis approaches. The features that differed between EOTRH degrees regardless of the filtering used were marked with an asterisk and the percentage of these features was shown in the right lower corner of each plot. 

## 3. Results

**Figure 3:**
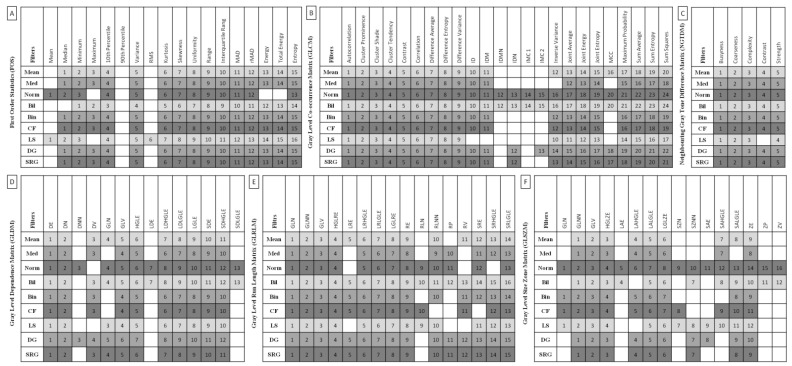
Features of First Order Statistics (FOS, **A**), Gray Level Co-occurrence Matrix (GLCM, **B**), Neighbouring Gray Tone Difference Matrix (NGTDM, **C**), Gray Level Dependence Matrix (GLDM, **D**), Gray Level Run Length Matrix (GLRLM, **E**), and Gray Level Size Zone Matrix (GLSZM, **F**) extracted from examined output images, filtered by Mean, Median (Med), Normalize (Norm), Bilateral (Bil), Binomial (Bin), CurvatureFlow (CF), LaplacianSharpening (LS), DiscreteGaussian (DG), and SmoothingRecursiveGaussian (SRG) filters, found to be significantly different between the Equine odontoclastic tooth resorption and hypercementosis classes (EOTRH 0–3). The number in the cell indicates the number of features in the row that differ between classes.

Among 837 returned combinations of filtering (*n* = 9) and image texture features (*n* = 93, including FOS *n* = 18, GLCM *n* = 24, NGTDM *n* = 5, GLDM *n* = 14, GLRLM *n* = 16, and GLSZM *n* = 16), considering all filters used at least 13 features of FOS, 17 features of GLCM, 4 features of NGTDM, 10 features of GLDM, 13 features of GLRLM, and 8 features differed between the EOTRH classes. These differences were summarized in Figure 3 and considered for further analysis. For FOS, the most features differed after filtering by *LaplacianSharpening* filter (*n* = 16) and the least after filtering by the *Normalize* filter (*n* = 13). For GLCM, the most features differed after filtering by *Normalize* and *Bilateral* filters (*n* = 24) and the least after filtering by the *LaplacianSharpening* filter (*n* = 17). For NGTDM, only after filtering by the *LaplacianSharpening* filter, not all features (*n* = 4) differed between the EOTRH classes. For GLDM *n* = 14, the most features differed after filtering by *Normalize* and *Bilateral* filters (*n* = 13) and the least after filtering by *Median*, *Binomial*, *CurvatureFlow*, and *LaplacianSharpening* filters (*n* = 10). For GLRLM, the most features differed after filtering by a *Bilateral* filter (*n* = 16) and the least after filtering by *Median*, *Normalize*, *CurvatureFlow*, and *LaplacianSharpening* filters (*n* = 13). For GLSZM, the most features differed after filtering by the *Normalize* filter (*n* = 16) and the least after filtering by the *Median* filter (*n* = 8). The mean ± SD values of extracted features were calculated and presented in Supplementary Tables S1–S9 for FOS, Tables S10–S18 for GLCM, Tables S19–S27 for NGTDM, Tables S28–S36 for GLDM, Tables S37–S45 for GLRLM, and Tables S46–S54 for GLSZM, available online. 

**Figure 4:**
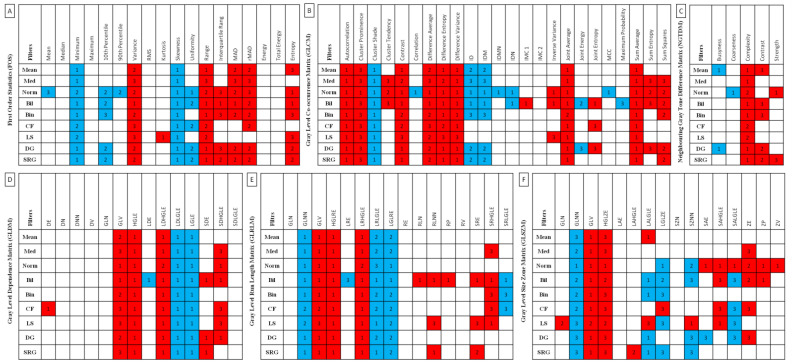
Features of First Order Statistics (FOS, **A**), Gray Level Co-occurrence Matrix (GLCM, **B**), Neighbouring Gray Tone Difference Matrix (NGTDM, **C**), Gray Level Dependence Matrix (GLDM, **D**), Gray Level Run Length Matrix (GLRLM, **E**), and Gray Level Size Zone Matrix (GLSZM, **F**) extracted from examined output images, filtered by Mean, Median (Med), Normalize (Norm), Bilateral (Bil), Binomial (Bin), CurvatureFlow (CF), LaplacianSharpening (LS), DiscreteGaussian (DG), and SmoothingRecursiveGaussian (SRG) filters, found to significantly increase (red cell) or decrease (blue cell) with the degree of the Equine odontoclastic tooth resorption and hypercementosis syndrome (EOTRH 0–3). The number in the cell indicates the degree of EOTRH from which the increase or decrease in the values of the features begins.

Among 683 returned combinations that basically differed between the EOTRH classes, 410 features increased or decreased with the degree of the EOTRH (including FOS *n* = 76, GLCM *n* = 123, NGTDM *n* = 19, GLDM *n* = 56, GLRLM *n* = 71, and GLSZM *n* = 65) (Figure 4). Then two criteria were used to indicate these features, which clearly correlate with the following EOTRH degrees: (i) the features that increase or decrease from class 1 of the EOTRH for at least one filter and (ii) the features that increase or decrease repeatable in each output images data set, regardless of the applied filtering. For FOS, the first criterion (an increase from class 1 of the EOTRH) passed: Variance, Kurtosis, Range, Interquartile range, MAD, and Entropy; as well as (a decrease from class 1 of the EOTRH): Minimum, Skewness, and Uniformity. For GLCM, the first criterion (an increase from class 1 of the EOTRH) was passed for the following: Autocorrelation, Cluster Prominence, Contrast, Difference Average, Difference Entropy, Difference Variance, IMC1, Inverse Variance, Joint Average, Joint Energy, and Sum Average; as well as (a decrease from class 1 of the EOTRH): Cluster Shade, Correlation, ID, IDM, IDMN, IDN, and MCC. For NGTDM, the first criterion (an increase from class 1 of the EOTRH) was passed for the following: Complexity and Strength; as well as (a decrease from class 1 of the EOTRH): Busyness and Coarseness. For GLDM, the first criterion (an increase from class 1 of the EOTRH) was passed for the following: DE, GLV, HGLE, LDHGLE, SDE, and SDHGLE; as well as (a decrease from class 1 of the EOTRH): LDE, LDLGLE, and LGLE. For GLRLM, the first criterion (an increase from class 1 of the EOTRH) was passed for the following: GLV, HGLR, LRHGLR, RLN, RLNN, RP, SRE, and SRHGLE; as well as (a decrease from class 1 of the EOTRH): GLNN, LRLGL, LGLRE, and SRLGLE. Finally, for GLSZM, the first criterion (an increase from class 1 of the EOTRH) was passed for the following: GLV, HGLZE, LALGLE, SZNN, SAE, SAHGLE, SALGLE, ZP, and ZV; as well as (a decrease from class 1 of the EOTRH): GLNN, LALGLE, and LGLZE.

One may observe that the part of the listed features passed the second criterion (increase or decrease repeatable in each output image data set, regardless of the applied filtering) and thus were considered to clearly correlate with the EOTRH degree. For FOS, the second criterion passed four of the following nine listed features: Variance, Range, Maximum, and Skewness. For GLCM, the second criterion passed 9 of the following 18 listed features: Autocorrelation, Cluster Prominence, Contrast, Difference Average, Difference Entropy, Difference Variance, Joint Average, Sum Average, and Cluster Shade. For NGTDM, the second criterion passed one of the following four listed features: Complexity. For GLDM, the second criterion passed five of the following nine listed features: GLV, HGLE, LDHGLE, LDLGLE, and LGLE. From GLRLM, the second criterion passed 6 of the following 12 listed features: GLV, HGLRE, LRHGLE, GLNN, LRLGLE, and LGLRE. Finally, for GLSZM, the second criterion passed 3 of the following 12 listed features increase: GLV, HGLZE, and GLNN.

**Figure 5:**
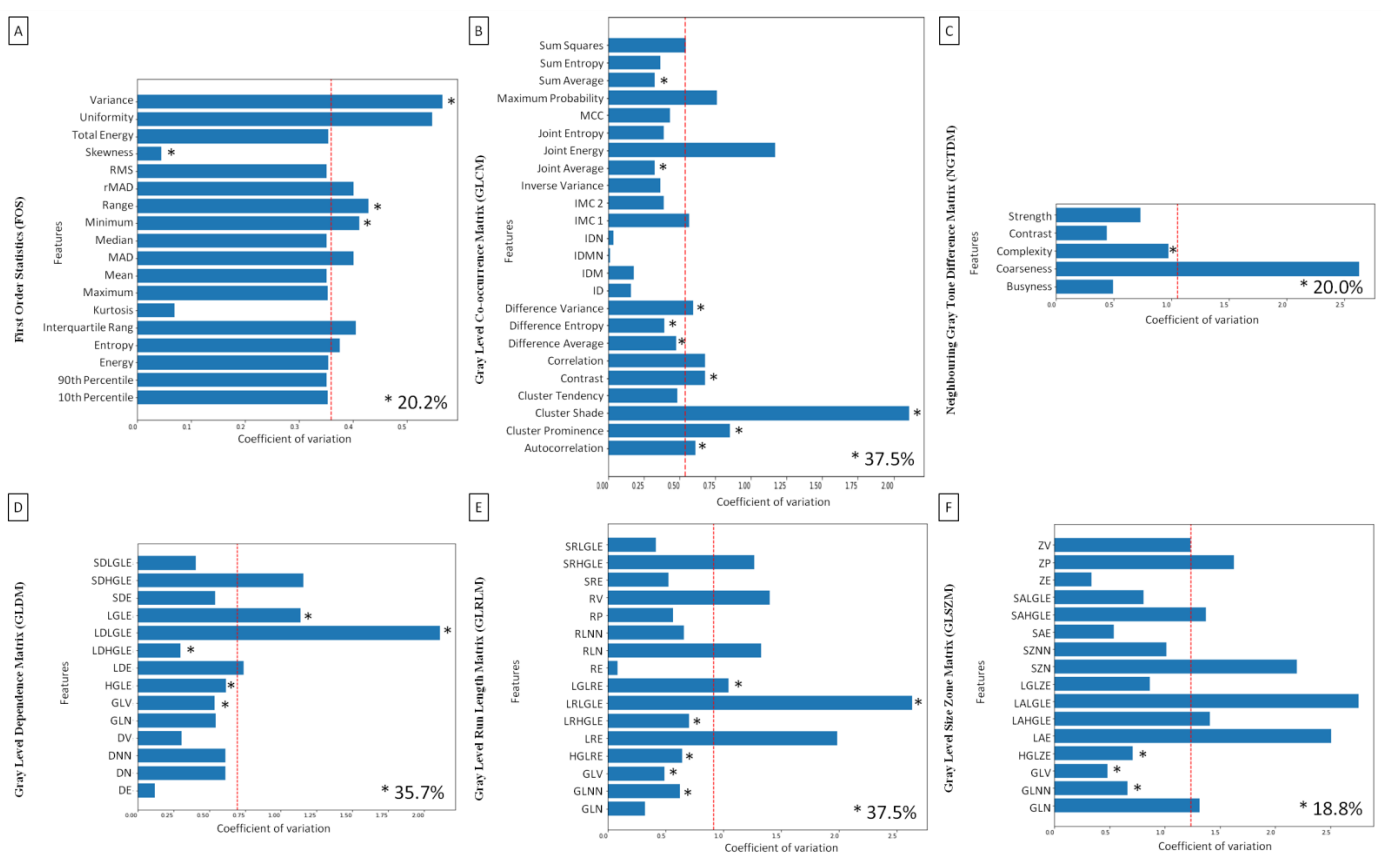
Coefficient of variation for the consecutive features of First Order Statistics (FOS, **A**), Gray Level Co-occurrence Matrix (GLCM, **B**), Neighbouring Gray Tone Difference Matrix (NGTDM, **C**), Gray Level Dependence Matrix (GLDM, **D**), Gray Level Run Length Matrix (GLRLM, **E**), and Gray Level Size Zone Matrix (GLSZM, **F**) including nine filters used. The red dashed line indicates the mean value of the feature for the total data set of consecutive texture analysis approaches. The asterisk indicates the features that differed between Equine odontoclastic tooth resorption and hypercementosis (EOTRH) degree regardless of the filtering used. The percentage of features marked with an asterisk is shown in the right low corner of each plot.

Then, the coefficient of variation was used to indicate which understudied texture analysis approaches could be most favorable used in further research for the quantification of radiographic signs of the EOTRH syndrome. The texture features that passed both criteria indicating the EOTRH degree (FOS *n* = 4, GLCM *n* = 9, NGTDM *n* = 1, GLDM *n* = 5, GLRLM *n* = 6, and GLSZM *n* = 3) were marked with the asterisks on the plots in Figure 5. One may observe that the coefficient of variation of the selected features was partially lower and partially higher than the mean value of the feature for the total data set of consecutive texture analysis approaches, marked with the red dashed. For FOS, one feature was lower and three features were higher than the mean coefficient of variation of FOS, and repetitively variable features accounted for 20.2% of all extracted FOS features. For GLCM, four features were lower, and five features were higher than the mean coefficient of variation of GLCM, and repetitively variable features accounted for 37.5% of all extracted GLCM features. For NGTDM, the only one feature was lower than the mean coefficient of variation of NGTDM and accounted for 20.0% of all extracted NGTDM features. For GLDM, three features were lower, and two features were higher than the mean coefficient of variation of GLDM, and repetitively variable features accounted for 35.7% of all extracted GLDM features. For GLRLM, four features were lower and five features were higher than the mean coefficient of variation of GLRLM, and repetitively variable features accounted for 37.5% of all extracted GLRLM features. Finally, for GLSZM, three features were lower than the mean coefficient of variation of GLSZM, and repetitively variable features accounted for 18.8% of all extracted GLSZM features. One may observe that GLCM and GLRLM return the most favorable features for the quantitative evaluation of radiographic signs of the EOTRH syndrome, and thus could be used in further research on the advanced radiographic diagnosis of equine dental diseases.

**Figure 6:**
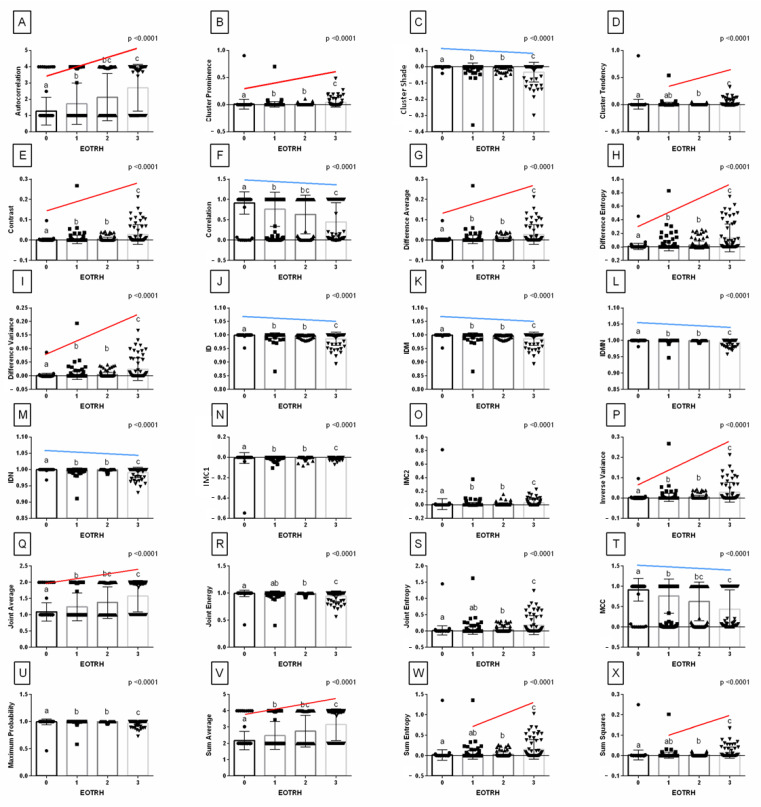
Features (mean ± SD) of *Gray Level Co-occurrence Matrix* (GLCM) extracted from examined output images, filtered by *Normalize* filter compared between the degrees of the Equine odontoclastic tooth resorption and hypercementosis syndrome (EOTRH 0–3). Autocorrelation (**A**), Cluster Prominence (**B**), Cluster Shade (**C**), Cluster Tendency (**D**), Contrast (**E**), Correlation (**F**), Difference Average (**G**), Difference Entropy (**H**), Difference Variance (**I**), Inverse difference (ID, **J**), Inverse Difference Moment (IDM, **K**), Inverse Difference Moment Normalized (IDMN, **L**), Inverse Difference Normalized (IDN, **M**), Informational Measure of Correlation 1 (IMC 1, **N**), Informational Measure of Correlation 2 (IMC 2, **O**), Inverse Variance (**P**), Joint Average (**Q**), Joint Energy (**R**), Joint Entropy (**S**), Maximal Correlation Coefficient (MCC, **T**), Maximum Probability (**U**), Sum Average (**V**), Sum Entropy (**W**), and Sum of Squares (**X**). Lower case letters (a–c) indicate differences between classes for *p* < 0.05 independently for each feature. The significant increase or decrease with the degree of the EOTRH 0–3 is marked with red and blue lines respectively. Single realizations are marked with dots.

**Figure 7:**
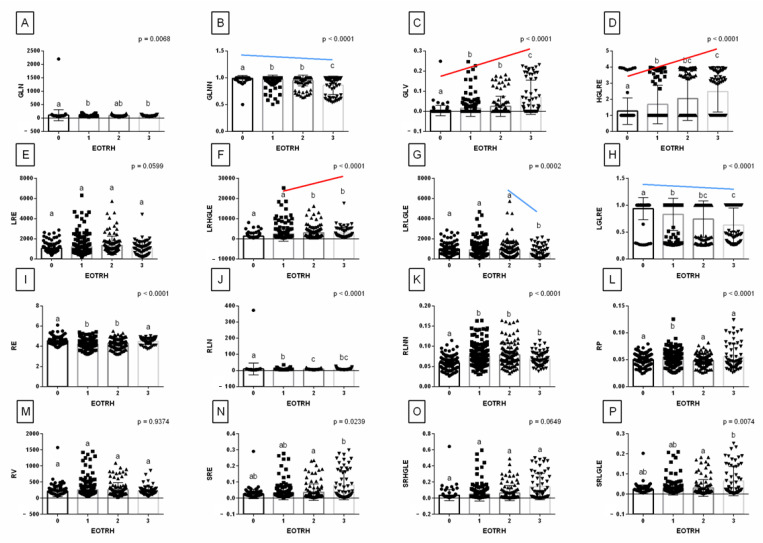
Features (mean ± SD) of *Gray Level Run Length Matrix (GLRLM)* extracted from examined output images, filtered by *Normalize* filter compared between the degrees of the Equine odontoclastic tooth resorption and hypercementosis syndrome (EOTRH 0–3). Gray Level Non-uniformity (GLN, **A**), Gray Level Non-uniformity Normalized (GLNN, **B**), Gray Level Variance (GLV, **C**), High Gray Level Run Emphasis (HGLRE, **D**), Long Run Emphasis (LRE, **E**), Long Run High Gray Level Emphasis (LRHGLE, **F**), Long Run Low Gray Level Emphasis (LRLGLE, **G**), Low Gray Level Run Emphasis (LGLRE, **H**); Run Entropy (RE, **I**), Run Length Non-uniformity (RLN, **J**), Run Length Non-uniformity Normalized (RLNN, **K**), Run Percentage (RP, **L**), Run Variance (RV, **M**), Short Run Emphasis (SRE, **N**), Short Run High Gray Level Emphasis (SRHGLE, **O**), Short Run Low Gray Level Emphasis (SRLGLE, **P**). Lower case letters (a–c) indicate differences between classes for *p* < 0.05 independently for each feature. The significant increase or decrease with the degree of the EOTRH 0–3 is marked with red and blue lines respectively. Single realizations are marked with dots.

Synthetically summarizing the above results, one may observe that features of GLCM and GLRLM extracted from the output images filtered by the *Normalize* filter are considered to be the best for imaging radiological symptoms of EOTRH. Therefore, the detailed comparisons of these features (Figure 6 and Figure 7) between EOTRH degrees have been presented as an example that can be used in future research.

Among the 24 features of GLCM extracted from the output images filtered by the *Normalize* filter, 16 features passed the first criterion of correlation with the EOTRH degree and increased or decreased from class 1 of the EOTRH: Autocorrelation, Cluster Prominence, Cluster Shade, Contrast, Correlation, Difference Average, Difference Entropy, Difference Variance, ID, IDM, IDMN, IDN, Inverse Variance, Joint Average, MCC, and Sum Average (Figure 6). These GLCM features were considered better than others to quantify the radiographic signs of the EOTRH syndrome.

**Figure 8:**
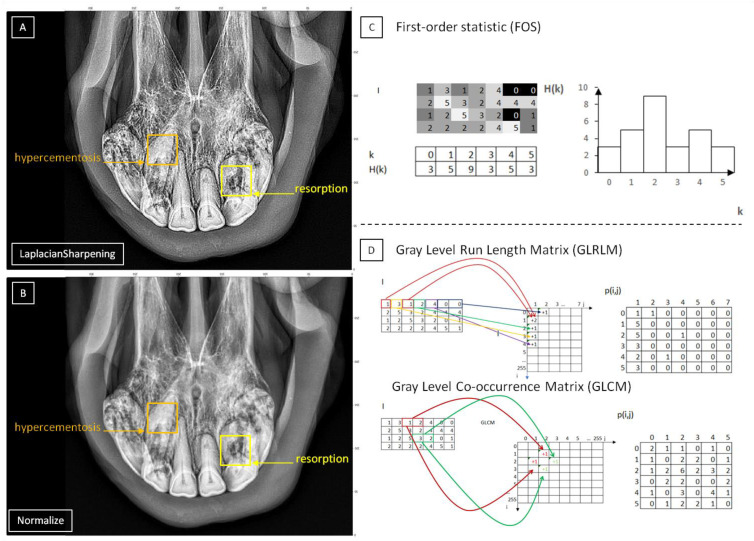
The samples of radiographic images representing severe Equine odontoclastic tooth resorption and hypercementosis (EOTRH 3) syndrome. The output images filtered by *LaplacianSharpening* (**A**) and *Normalize* (**B**) filters with marked signs of resorption (yellow square) and hypercementosis (orange square). The filtered images compiled with diagrams of optimal approaches of texture analysis, the first-order statistic (FOS) for *LaplacianSharpening* filter (**C**) and the second-order statistic for *Normalize* filter (**D**).

Among 16 features of GLRLM extracted from the output images filtered by the *Normalize* filter, the following 4 features passed the first criterion of correlation with the EOTRH degree and increased or decreased from class 1 of the EOTRH: GLNN, GLV, HGLRE, and LGLRE (Figure 8). These GLRLM features were considered better than others to quantify the radiographic signs of the EOTRH syndrome.

## 4. Discussion

In both studies, the previous [13,16,18,22] and current resorptive lesions of variable degree and mild to severe hypercementosis were radiographically recognized. The impact of the resorption and hypercementosis processes on the texture of the radiological images is visually easily separable [13,18,22]. The tooth resorption process involves the cementum, enamel, dentine, and occasionally the pulp cavity [15], which is visible on the radiological images as a loss of the radiodensity of dental tissue and alveolar bone. Radiographically, the widening of the periodontal ligament space is visible as a radiolucent line and the alveolar bone appears as a delicate radiodense line, which is typical [22]. The hypercementosis reflects the cement accumulation, which often appears as radiopaque bulbous enlargements of the apex of the tooth [59]. In the cases of cement accumulation, the extensive bulbs are created within an increase in density in this place, so the radiological images show mottled radiolucent areas [60]. Thus, we hypothesize that the radiographic signs of the EOTRH syndrome could be successively quantified as the statistical decryptions of the medical digital image give very good results in a large field of applications [24], especially when the textures are visually easily separable [26].

Most features of the second-order statistic (GLCM, NGTDM, GLDM, GLRLM, and GLSZM) differed between the EOTRH classes after filtering by the *Normalize* and *Bilateral* filters (GLCM, GLDM) or the *Normalize* filter (GLSZM) or the *Bilateral* filter (GLRLM), contrary to the features of first-order statistic (FOS) where they differed least after filtering by *Normalize* filter. On the other hand, the least features of the second-order statistic differed between the EOTRH classes after filtering by the *LaplacianSharpening* filter (GLCM, NGTDM, GLDM, and GLRLM), which was the most differential filter, was just the *LaplacianSharpening* filter. As the *LaplacianSharpening* filter returns output images sharper than the input one [61], this filter appears to be more suitable for the first-order statistic than the second-order one. Interestingly, moreover, the *Normalize* filter increases the contrast of the image [62] and *Bilateral* filter smooths the input image in the homogeneous areas while preserving the edges [63], which seems to be favorable for the second-order statistic (Figure 8). 

This other differentiation of the incisor teeth after filtering by various filters may be caused by the characteristics of particular approaches to texture analysis. The FOS examines the pixels that are present in the image, while the GLCM and the other second-order matrices examine the spatial distribution of the pixels [64]. Therefore, when the filter returns the output images after sharpening or improving the contrast, the feature differences will be greater [65,66]. On the contrary, other filters tested in the current study, which returns the output images after noise reduction (*Mean*, *Median,* and *CurvatureFlow* filters), blurring (*Mean* and *DiscreteGaussian* filters), smoothing (*Median, CurvatureFlow*, *DiscreteGaussian,* and *SmoothingRecursiveGaussian* filters), or blur separation (*Binomial* filter) [67]. This means that if the second-order statistical approaches are chosen for the texture analysis of the radiographic image, the use of one or both filters that improve the edge delimitation is advisable, as the other examined filters appear to be less desirable in the quantification of radiographic signs of the EOTRH syndrome.

Concerning the texture features that increase or decrease repeatability regardless of the applied filtering, each applied texture analysis approach returns the specific feature profile. Beginning from the lowest percentage of selected features, GLSZM (18.8%), NGTDM (20.0%), FOS (20.2%), GLDM (35.7%), and equally, GLRLM (37.5%) and GLCM (37.5%) were ranked. For GLSZM, the increase in GLV and HGLZE, as well as the decrease in GLNN with the increase in the EOTRH degree, were noted. Although the GLV indicates the increase in the variance in gray level intensities for the zones [37], the role of the other two features requires further study. HGLZE counts the distribution of lower/higher gray-level size zones together with LGLZE, whereas GLNN measures the variability of gray-level intensity values together with GLN [37]. As LGLZE and GLN did not change repetitively, GLSZM should not be the recommended approach for the quantitative EOTRH evaluation. For NGTDM, only Complexity, the measure of the occurrence of many primitive components in the image [37], increased repetitively. Thus, the NGTDM approach is also difficult to recommend. For FOS, with the increase in the EOTRH degree, the increase in Variance, which is the mean of squared distances between each pixel and the mean value of pixels [37], and the increase in Range, which is the difference between the maximum and minimum values of pixels [37], were noted. Moreover, with the increase in the EOTRH degree, a decrease in the Minimum and Skewness was observed. As the Minimum describes the specific intensity values in the image [37], with its decrease, the increase in Range is justified, especially since no similar differences were found for Maximum, 10th percentile, and 90th percentile, the other indicators of specific intensity. Likewise, the increase in Skewness, which is an indicator of the asymmetry of the distribution of values from the mean value [38], may be related to the Variance increase, as these two features are negatively correlated [37]. Therefore, recommending the FOS approach for the quantitative EOTRH evaluation is also questionable. The pattern of GLDM features appears to be much more reproducible, as the related features increase (HGLE and LDHGLE) and decrease (LGLE and LDLGLE) with the EOTRH degree, respectively. It should be noted that LGLE and HGLE are measures of the distribution of low/high gray level values [38], whereas LDLGLE and LDHGLE are measures the joint distribution of large dependences with low/high gray-level values [37]. Moreover, GLV measuring variance in gray level [37] also increased with the EOTRH degree, which may suggest the diversity of grayscale distribution in the radiographic images [68] typical for tooth resorption and hypercementosis [13,22,23], which requires further study. A similar, though broader, pattern was represented by GLRLM features, where the related features increased (HGLRE and LRHGLE) and decreased (LGLRE and LRLGLE) with the EOTRH degree, as was shown in the *Normalize* filtered sample of detailed comparisons. It should be noted that LGLRE and HGLRE are measures of the distribution of low/high gray level values [37], whereas LRLGLE and LRHGLE are measures of the joint distribution of long run lengths with low/high gray level values [37]. Moreover, similarly to GLDM, the increase in GLV, measuring of the variance in gray level intensity for the runs [38], and additionally, the decrease in GLNN, measuring the similarity of gray-level intensity values in the image [37], were noted. One may conclude that such a repetition, in GLDM and GLRLM, of similar measures the diversity of grayscale distribution in the radiographic images may be an important direction in the quantification of radiographic signs of the EOTRH syndrome [13,22,23]. For GLCM, with the increase in the EOTRH degree, the increase in Autocorrelation, Cluster Prominence, Contrast, Difference Average, Difference Entropy, Difference Variance, Joint Average, and Sum Average was noted. The most selected features, such as Contrast showing the local intensity variation, Difference Average indicating the relationship between the occurrence of pairs with similar/differing intensity values, Difference Entropy, showing the randomness in neighbourhood intensity value differences, Joint Average, reporting the mean level intensity of the *i* distribution, and Sum Average, measuring the relation between pairs with lower/higher intensity values [37], reflect the intensity of the pixels of the radiographic images. One may observe that not only the intensity measures but also the coarseness (Autocorrelation) and heterogeneity (Difference Variance) measures [37] increased with the EOTRH degree, which may be a promising indicator of changes in the radiographic image caused by tooth resorption and hypercementosis [13,22,23]. However, the role of the skewness/asymmetry/uniformity of the GLCM, reflected by Cluster Prominence and Cluster Shade [37,38], requires further research. 

Given the presented volatility of the coefficient of variation and the percentage of the repetitively variable features within the consecutive approaches and their specific patterns, one may state that the selection of the filtering algorithms and texture analysis approaches for the quantification of radiographic signs of the EOTRH syndrome should include GLCM and GLRLM analysis of the output images received after filtering by the *Normalize* or *Bilateral* filters. However, further research is needed to evaluate the usefulness of the texture analysis of radiographic images of equine incisor teeth concerning clinical symptoms such as varying degrees of oral pain, periodontitis, gingivitis, gingival hyperplasia or recession, fistulas, often in combination with a focal subepithelial swelling (referred to as parulis or gum boils), bulbous enlargement of dental structures, tooth mobility, tooth fractures, and missing teeth [13,69,70]. Moreover, the optimal filtering and texture analysis approaches should be established for the detection of other equine tooth diseases and malocclusions such as sharp edges on the cheek teeth, hooks, excessively protruding clinical crowns, excessive transverse ridges, wave mouth, step mouth, diastemata, persistent milk teeth, loose teeth, tooth fractures, angled teeth, periodontitis, caries, oligodontia, polydontia, calculus, tooth deformities, underbite, overbite, erosions or ulcers on the cheek or tongue mucosa, curvature of the incisors, unerupted canines, and wolf teeth [7,10]. However, the computed tomography imaging of a horse’s head is preferred to limit the radiological superimposition arising from the anatomical structure of the horse’s skull [20], since the diagnostic accuracy of computed tomography of equine cheek teeth is much higher compared with radiography for this disorder [71]. Thus, one may concern with the current two-dimensional imaging and analysis as the preliminary before the further three-dimensional study, as the incisor teeth, unlike the cheek teeth, are easier to visually and radiography assess due to the rostral position [8].

## 5. Conclusions

The radiographic images of the equine maxillary incisor teeth may be successfully digitally processed using different filtering algorithms and then analyzed by texture features extraction based on the first- and second-order statistics. Within six studied texture analysis approaches, GLCM and GLRLM return the most favorable features for the quantitative evaluation of radiographic signs of the EOTRH syndrome. Based on the GLCM analysis, one may suggest that the measures of the radiographic image intensity, coarseness, and heterogeneity increase with the EOTRH degree. Moreover, based on the GLRLM analysis, one may observe that the diversity of grayscale distribution reflected by the opposing texture features may reveal the advancement of both the tooth resorption and hypercementosis processes visible on the radiographic images. It is worth noting that the application of GLCM and GLRLM as the most advisable for the quantification of radiographic signs of the EOTRH syndrome may be supported by filtering by filters improving the edge delimitation, such as *Normalize* or *Bilateral* filters.

## Data Availability

The data presented in this study are available on request from the corresponding author.

## Supplementary Materials

The following are available online at https://www.mdpi.com/article/10.3390/s22082920/s1. Table S1: The values of features of *First Order Statistics* (FOS) of output images, filtrated by *Mean* filter between four classes of the EOTRH syndrome, Table S2: The values of features of *First Order Statistics* (FOS) of output images, filtrated by *Median* filter between four classes of the EOTRH syndrome, Table S3: The values of features of *First Order Statistics* (FOS) of output images, filtrated by *Normalize* filter between four classes of the EOTRH syndrome, Table S4: The values of features of *First Order Statistics* (FOS) of output images, filtrated by *Bilateral* filter between four classes of the EOTRH syndrome, Table S5: The values of features of *First Order Statistics* (FOS) of output images, filtrated by *Binomial* filter between four classes of the EOTRH syndrome, Table S6: The values of features of *First Order Statistics* (FOS) of output images, filtrated by *CurvatureFlow* filter between four classes of the EOTRH syndrome, Table S7: The values of features of *First Order Statistics* (FOS) of output images, filtrated by *LaplacianSharpening* filter between four classes of the EOTRH syndrome, Table S8: The values of features of *First Order Statistics* (FOS) of output images, filtrated by *DiscreteGaussian* filter between four classes of the EOTRH syndrome, Table S9: The values of features of *First Order Statistics* (FOS) of output images, filtrated by *SmoothingRecursiveGaussian* filter between four classes of the EOTRH syndrome, Table S10: The values of features of *Gray Level Co-occurence Matrix* (GLCM) of output images, filtrated by *Mean* filter between four classes of the EOTRH syndrome, Table S11: The values of features of *Gray Level Co-occurence Matrix* (GLCM) of output images, filtrated by *Median* filter between four classes of the EOTRH syndrome, Table S12: The values of features of *Gray Level Co-occurence Matrix* (GLCM) of output images, filtrated by *Normalize* filter between four classes of the EOTRH syndrome, Table S13: The values of features of *Gray Level Co-occurence Matrix* (GLCM) of output images, filtrated by *Bilateral* filter between four classes of the EOTRH syndrome, Table S14: The values of features of *Gray Level Co-occurence Matrix* (GLCM) of output images, filtrated by *Binomial* filter between four classes of the EOTRH syndrome, Table S15: The values of features of *Gray Level Co-occurence Matrix* (GLCM) of output images, filtrated by *CurvatureFlow* filter between four classes of the EOTRH syndrome, Table S16: The values of features of *Gray Level Co-occurence Matrix* (GLCM) of output images, filtrated by *LaplacianSharpening* filter between four classes of the EOTRH syndrome, Table S17: The values of features of *Gray Level Co-occurence Matrix* (GLCM) of output images, filtrated by *DiscreteGaussian* filter between four classes of the EOTRH syndrome, Table S18: The values of features of *Gray Level Co-occurence Matrix* (GLCM) of output images, filtrated by *SmoothingRecursiveGaussian* filter between four classes of the EOTRH syndrome, Table S19: The values of features of *Neighbouring Gray Tone Difference Matrix* (NGTDM) of output images, filtrated by *Mean* filter between four classes of the EOTRH syndrome, Table S20: The values of features of *Neighbouring Gray Tone Difference Matrix* (NGTDM) of output images, filtrated by *Median* filter between four classes of the EOTRH syndrome, Table S21: The values of features of *Neighbouring Gray Tone Difference Matrix* (NGTDM) of output images, filtrated by *Normalize* filter between four classes of the EOTRH syndrome, Table S22: The values of features of *Neighbouring Gray Tone Difference Matrix* (NGTDM) of output images, filtrated by *Bilateral* filter between four classes of the EOTRH syndrome, Table S23: The values of features of *Neighbouring Gray Tone Difference Matrix* (NGTDM) of output images, filtrated by *Binomial* filter between four classes of the EOTRH syndrome, Table S24: The values of features of *Neighbouring Gray Tone Difference Matrix* (NGTDM) of output images, filtrated by *CurvatureFlow* filter between four classes of the EOTRH syndrome, Table S25: The values of features of *Neighbouring Gray Tone Difference Matrix* (NGTDM) of output images, filtrated by *LaplacianSharpening* filter between four classes of the EOTRH syndrome, Table S26: The values of features of *Neighbouring Gray Tone Difference Matrix* (NGTDM) of output images, filtrated by *DiscreteGaussian* filter between four classes of the EOTRH syndrome, Table S27: The values of features of *Neighbouring Gray Tone Difference Matrix* (NGTDM) of output images, filtrated by *SmoothingRecursiveGaussian* filter between four classes of the EOTRH syndrome, Table S28: The values of features of *Gray Level Dependence Matrix* (GLDM) of output images, filtrated by *Mean* filter between four classes of the EOTRH syndrome, Table S29: The values of features of *Gray Level Dependence Matrix* (GLDM) of output images, filtrated by *Median* filter between four classes of the EOTRH syndrome, Table S30: The values of features of *Gray Level Dependence Matrix* (GLDM) of output images, filtrated by *Normalize* filter between four classes of the EOTRH syndrome, Table S31: The values of features of *Gray Level Dependence Matrix* (GLDM) of output images, filtrated by *Bilateral* filter between four classes of the EOTRH syndrome, Table S32: The values of features of *Gray Level Dependence Matrix* (GLDM) of output images, filtrated by *Binomial* filter between four classes of the EOTRH syndrome, Table S33: The values of features of *Gray Level Dependence Matrix* (GLDM) of output images, filtrated by *CurvatureFlow* filter between four classes of the EOTRH syndrome, Table S34: The values of features of *Gray Level Dependence Matrix* (GLDM) of output images, filtrated by *LaplacianSharpening* filter between four classes of the EOTRH syndrome, Table S35: The values of features of *Gray Level Dependence Matrix* (GLDM) of output images, filtrated by *DiscreteGaussian* filter between four classes of the EOTRH syndrome, Table S36: The values of features of *Gray Level Dependence Matrix* (GLDM) of output images, filtrated by *SmoothingRecursiveGaussian* filter between four classes of the EOTRH syndrome, Table S37: The values of features of *Gray Level Run Length Matrix* (GLRLM) of output images, filtrated by *Mean* filter between four classes of the EOTRH syndrome, Table S38: The values of features of *Gray Level Run Length Matrix* (GLRLM) of output images, filtrated by *Median* filter between four classes of the EOTRH syndrome, Table S39: The values of features of *Gray Level Run Length Matrix* (GLRLM) of output images, filtrated by *Normalize* filter between four classes of the EOTRH syndrome, Table S40: The values of features of *Gray Level Run Length Matrix* (GLRLM) of output images, filtrated by *Bilateral* filter between four classes of the EOTRH syndrome, Table S41: The values of features of *Gray Level Run Length Matrix* (GLRLM) of output images, filtrated by *Binomial* filter between four classes of the EOTRH syndrome, Table S42: The values of features of *Gray Level Run Length Matrix* (GLRLM) of output images, filtrated by *CurvatureFlow* filter between four classes of the EOTRH syndrome, Table S43: The values of features of *Gray Level Run Length Matrix* (GLRLM) of output images, filtrated by *LaplacianSharpening* filter between four classes of the EOTRH syndrome, Table S44: The values of features of *Gray Level Run Length Matrix* (GLRLM) of output images, filtrated by *DiscreteGaussian* filter between four classes of the EOTRH syndrome, Table S45: The values of features of *Gray Level Run Length Matrix* (GLRLM) of output images, filtrated by *SmoothingRecursiveGaussian* filter between four classes of the EOTRH syndrome, Table S46: The values of features of *Gray Level Size Zone Matrix* (GLSZM) of output images, filtrated by *Mean* filter between four classes of the EOTRH syndrome, Table S47: The values of features of *Gray Level Size Zone Matrix* (GLSZM) of output images, filtrated by *Median* filter between four classes of the EOTRH syndrome, Table S48: The values of features of *Gray Level Size Zone Matrix* (GLSZM) of output images, filtrated by *Normalize* filter between four classes of the EOTRH syndrome, Table S49: The values of features of *Gray Level Size Zone Matrix* (GLSZM) of output images, filtrated by *Bilateral* filter between four classes of the EOTRH syndrome, Table S50: The values of features of *Gray Level Size Zone Matrix* (GLSZM) of output images, filtrated by *Binomial* filter between four classes of the EOTRH syndrome, Table S51: The values of features of *Gray Level Size Zone Matrix* (GLSZM) of output images, filtrated by *CurvatureFlow* filter between four classes of the EOTRH syndrome, Table S52: The values of features of *Gray Level Size Zone Matrix* (GLSZM) of output images, filtrated by *LaplacianSharpening* filter between four classes of the EOTRH syndrome, Table S53: The values of features of *Gray Level Size Zone Matrix* (GLSZM) of output images, filtrated by *DiscreteGaussian* filter between four classes of the EOTRH syndrome, Table S54: The values of features of *Gray Level Size Zone Matrix* (GLSZM) of output images, filtrated by *SmoothingRecursiveGaussian* filter between four classes of the EOTRH syndrome.

## References

[1] Dixon P.M., Dacre I. (2005). A review of equine dental disorders. Vet. J..

[2] Knottenbelt D.C., Baker G.J., Easley J. (1999). The systemic effects of dental disease. Equine Dentistry.

[3] Kirkland K.D., Maretta S.M., Inoue O.J., Baker G.J. Survey of equine dental disease and associated oral pathology. Proceedings of the 40th Annual Convention of the American Association of Equine Practitioners.

[4] Lowder M.Q., Mueller P.O.E. (1998). Dental disease in geriatric horses. Vet. Clin. North Amer. Equine Pract..

[5] Peters J.W.E., de Boer B., Broeze-ten G.B.M., Broeze J., Wiemer P., Sterk T., Spoormakers T.J.P. (2006). Survey of Common Dental Abnormalities in 483 Horses in the Netherlands, Proceedings of the American Association of Equine Practitioners-Equine Dentistry Focus Meeting, Indianapolis, IN, USA, 1 August 2006.

[6] Pimentel L.F.R.O., Zopa A., Alves G.E.S., Amaral R.F. (2007). Equine dental disorders: Review of 607 cases. Pesqui. Vet. Bras..

[7] Dixon P.M., Tremaine W.H., Pickles K., Kuhns L., Hawe C., McCann J., McGorum B., Railton D.I., Brammer S. (1999). Equine dental disease Part 1: A longterm study of 400 cases: Disorders of incisor, canine and first premolar teeth. Equine Vet. J..

[8] Dixon P.M., Tremaine W.H., Pickles K., Kuhns L., Hawe C., McCmn J., McGorum B.C., Railton D.I., Brammer S. (1999). Equine dental disease Part 3: A long term study of 400 case: Disorders of wear, traumatic and idiopathic fractures, tumours and miscellaneous disorders of the cheek teeth. Equine Vet. J..

[9] Uhlinger C. Survey of selected dental abnormalities in 233 horses. Proceedings of the 33rd Annual Meeting of the Association of Equine Practitioners.

[10] Wilson G.J., Liyou O.J. (2005). Examination of dental charts of horses presented for routine dentistry over a 12 month period. Austr. Equine Vet..

[11] Maslauskas K., Tulamo R.M., McGowan T., Kučinskas A. (2008). A descriptive study of the dentition of Lithuanian heavy-drought horses. Vet. Ir Zootech..

[12] Hole S.L., Staszyk C. (2018). Equine odontoclastic tooth resorption and hypercementosis. Equine Vet. Educ..

[13] Rehrl S., Schröder W., Müller C., Staszyk C., Lischer C. (2018). Radiological prevalence of equine odontoclastic tooth resorption and hypercementosis. Equine Vet. J..

[14] Rawlinson J., Carmalt J.L. (2014). Extraction techniques for equine incisor and canine teeth. Equine Vet. Educ..

[15] Staszyk C., Bienert A., Kreutzer R., Wohlsein P., Simhofer H. (2008). Equine odontoclastic tooth resorption and hypercementosis. Vet. J..

[16] Sykora S., Pieber K., Simhofer H., Hackl V., Brodesser D., Brandt S. (2014). Isolation of Treponema and Tannerella spp. from equine odontoclastic tooth resorption and hypercementosis related periodontal disease. Equine Vet. J..

[17] Rahmani V.H., Häyinen L., Kareinen I., Ruohoniemi M. (2019). History, clinical findings and outcome of horses with radiographical signs of equine odontoclastic tooth resorption and hypercementosis. Vet. Rec..

[18] Górski K., Tremaine H., Obrochta B., Buczkowska R., Turek B., Bereznowski A., Rakowska A., Polkowska I. (2021). EOTRH syndrome in polish half-bred horses-two clinical cases. J. Equine Vet. Sci..

[19] Saccomanno S., Passarelli P.C.B., Oliva B., Grippaudo C. (2018). Comparison between two radiological methods for assessment of tooth root resorption: An in vitro study. Biomed Res. Int..

[20] Barrett M.F., Easley J.T. (2013). Acquisition and interpretation of radiographs of the equine skull. Equine Vet. Educ..

[21] Moore N.T., Schroeder W., Staszyk C. (2016). Equine odontoclastic tooth resorption and hypercementosis affecting all cheek teeth in two horses: Clinical and histopathological findings. Equine Vet. Educ..

[22] Henry T.J., Puchalski S.M., Arzi B., Kass P.H., Verstraete F.J.M. (2016). Radiographic evaluation in clinical practice of the types and stage of incisor tooth resorption and hypercementosis in horses. Equine Vet. J..

[23] Hüls I., Bienert A., Staszyk C. Equine odontoclastic tooth resorption and hyper-cementosis (EOTRH): Röntgenologische und makroskopisch-anatomische Befunde. Proceedings of the 10. Jahrestagung der Internationalen Gesellschaft zur Funktionsverbesserung der Pferdezähne.

[24] Mohanaiah P., Sathyanarayana P., GuruKumar L. (2013). Image texture feature extraction using GLCM approach. Int. J. Sci. Res..

[25] Wazarkar S., Keshavamurthy B.N. (2018). A survey on image data analysis through clustering techniques for real world applications. J. Vis. Commun. Image Represent..

[26] Maillard P. (2003). Comparing texture analysis methods through classification. Photogramm. Eng. Remote Sens..

[27] Sohail A.S.M., Bhattacharya P., Mudur S.P., Krishnamurthy S. Local relative GLRLM-based texture feature extraction for classifying ultrasound medical images. Proceedings of the 2011 24th Canadian Conference on Electrical and Computer Engineering (CCECE, IEEE).

[28] Abdel-Nasser M., Moreno A., Puig D. (2019). Breast cancer detection in thermal infrared images using representation learning and texture analysis methods. Electronics.

[29] Bębas E., Borowska M., Derlatka M., Oczeretko E., Hładuński M., Szumowski P., Mojsak M. (2021). Machine-learning-based classification of the histological subtype of non-small-cell lung cancer using MRI texture analysis. Biomed. Signal. Process. Control..

[30] Zhang H., Hung C.L., Min G., Guo J.P., Liu M., Hu X. (2019). GPU-accelerated GLRLM algorithm for feature extraction of MRI. Sci. Rep..

[31] Raja J.V., Khan M., Ramachandra V.K., Al-Kadi O. (2012). Texture analysis of CT images in the characterization of oral cancers involving buccal mucosa. Dentomaxillofac. Radiol..

[32] Girejko G., Borowska M., Szarmach J. Statistical analysis of radiographic textures illustrating healing process after the guided bone regeneration surgery. Proceedings of the International Conference on Information Technologies in Biomedicine, Springer (ITIB’2018).

[33] Sangeetha M., Kumar K., Aljabr A.A. (2021). Image processing techniques in periapical dental X-ray image detection and classification. Webology.

[34] Domino M., Borowska M., Kozłowska N., Zdrojkowski Ł., Jasiński T., Smyth G., Maśko M. (2022). Advances in thermal image analysis for the detection of pregnancy in horses using infrared thermography. Sensors.

[35] Masko M., Borowska M., Domino M., Jasinski T., Zdrojkowski L., Gajewski Z. (2021). A novel approach to thermographic images analysis of equine thoracolumbar region: The effect of effort and rider’s body weight on structural image complexity. BMC Vet. Res..

[36] Domino M., Borowska M., Kozłowska N., Trojakowska A., Zdrojkowski Ł., Jasiński T., Smyth G., Maśko M. (2022). Selection of image texture analysis and color model in the advanced image processing of thermal images of horses following exercise. Animals.

[37] Zwanenburg A., Leger S., Vallieres M., Lock S. (2016). Image biomarker standardisation initiative for image biomarker standardisation initiative. arXiv.

[38] Humeau-Heurtier A. (2019). Texture feature extraction methods: A survey. IEEE Access.

[39] Lowekamp B.C., Chen D.T., Ibáñez L., Blezek D. (2013). The design of SimpleITK. Front. Neuroinform..

[40] Yaniv Z., Lowekamp B.C., Johnson H.J., Beare R. (2018). SimpleITK image-analysis notebooks: A collaborative environment for education and reproducible research. J. Digit. Imaging.

[41] Belém M.D.F., Ambrosano G.M.B., Tabchoury C.P.M., Ferreira-Santos R.I., Haiter-Neto F. (2013). Performance of digital radiography with enhancement filters for the diagnosis of proximal caries. Braz. Oral Res..

[42] Geetha V., Aprameya K.S. (2019). Textural analysis based classification of digital X-ray images for dental caries diagnosis. Int. J. Eng. Manuf..

[43] Floyd M.R. (1991). The modified Triadan system: Nomenclature for veterinary dentistry. J. Vet. Dent..

[44] Beare R., Lowekamp B., Yaniv Z. (2018). Image segmentation, registration and characterization in R with SimpleITK. J. Stat. Soft..

[45] Lim J.S. (1990). Two-Dimensional Signal and Image Processing.

[46] Tomasi C., Manduchi R. Bilateral filtering for gray and color images. Proceedings of the Sixth International Conference on Computer Vision (IEEE Cat. No. 98CH36271).

[47] Aubury M., Luk W. (1996). Binomial filters. J. VLSI Signal Process. Syst. Signal Image Video Technol..

[48] Sethian J.A. (1999). Level Set Methods and Fast Marching Methods: Evolving Interfaces in Computational Geometry, Fluid Mechanics, Computer Vision, and Materials Science.

[49] Gonzalez R.C., Eddins S.L., Woods R.E. (2004). Digital Image Publishing Using MATLAB.

[50] Lindeberg T. (1991). Discrete Scale-Space Theory and the Scale-Space Primal Sketch. Ph.D. Thesis.

[51] Deriche R. Recursively implementating the gaussian and its derivatives. Proceedings of the IEEE International Conference on Image Processing (ICIP).

[52] van Griethuysen J.J.M., Fedorov A., Parmar C., Hosny A., Aucoin N., Narayan V., Beets-Tan R.G.H., Fillon-Robin J.C., Pieper S., Aerts H.J.W.L. (2017). Computational radiomics system to decode the radiographic phenotype. Cancer Res..

[53] Haralick R., Shanmugan K., Dinstein I. (1973). Textural features for image classification. IEEE Trans. Syst. Man Cybern..

[54] Amadasun M., King R. (1989). Textural features corresponding to textural properties. IEEE Trans. Syst. Man Cybern..

[55] Sun C., Wee W.G. (1983). Neighboring gray level dependence matrix for texture classification. Comput. Vis. Graph. Image Process..

[56] Galloway M.M. (1975). Texture analysis using gray level run lengths. Comput. Gr. Image Process..

[57] Chu A., Sehgal C.M., Greenleaf J.F. (1990). Use of gray value distribution of run length for texture analysis. Pattern Recognit. Lett..

[58] Thibault G., Fertil B., Navarro C., Pereira S., Cau P., Levy N., Sequeira J., Mari J.L. Texture indexes and gray level size zone matrix. application to cell nuclei classification. Proceedings of the 10th International Conference on Pattern Recognition and Information Processing, PRIP 2009.

[59] Smedley R.C., Earley E.T., Galloway S.S., Baratt R.M., Rawlinson J.E. (2015). Equine odon-toclastic tooth resorption and hypercementosis: Histopathologic features. Vet. Pathol..

[60] Baratt R. (2013). Advances in equine dental radiology. Vet. Clin. North Am. Equine Pract..

[61] Al-Ameen Z., Sulong G., Gapar M.D., Johar M.D. (2012). Reducing the Gaussian blur artifact from CT medical images by employing a combination of sharpening filters and iterative deblurring algorithms. J. Theor. Appl. Inf. Technol..

[62] Heidari M., Mirniaharikandehei S., Khuzani A.Z., Danala G., Qiu Y., Zheng B. (2020). Improving the performance of CNN to predict the likelihood of COVID-19 using chest X-ray images with preprocessing algorithms. Int. J. Med. Inform..

[63] Yang X., Sechopoulos I., Fei B. (2011). Automatic tissue classification for high-resolution breast CT images based on bilateral filtering. Proc. SPIE.

[64] Jusman Y., Tamarena R.I., Puspita S., Saleh E., Kanafiah S.N.A.M. Analysis of features extraction performance to differentiate of dental caries types using gray level co-occurrence matrix algorithm. Proceedings of the 2020 10th IEEE International Conference on Control System, Computing and Engineering (ICCSCE).

[65] Nagarajan M.B., Coan P., Huber M.B., Diemoz P.C., Glaser C., Wismüller A. (2014). Computer-aided diagnosis for phase-contrast X-ray computed tomography: Quantitative characterization of human patellar cartilage with high-dimensional geometric features. J. Digit. Imaging.

[66] Kociołek M., Strzelecki M., Obuchowicz R. (2020). Does image normalization and intensity resolution impact texture classification?. Comput. Med. Imaging Graph..

[67] Chandra T.B., Verma K. (2020). Analysis of quantum noise-reducing filters on chest X-ray images: A review. Measurement.

[68] Alzubaidi M.A., Otoom M. (2020). A comprehensive study on feature types for osteoporosis classification in dental panoramic radiographs. Comput. Methods Programs Biomed..

[69] Lorello O., Foster D.L., Levine D.G., Boyle A., Engiles J., Orsini J.A. (2016). Clinical treatment and prognosis of equine odontoclastic tooth resorption and hypercementosis. Equine Vet. J..

[70] Earley E., Rawlinson J.T. (2013). A new understanding of oral and dental disorders of the equine incisor and canine teeth. Vet. Clin. North Am. Equine Pract..

[71] Liuti T., Smith S., Dixon P.M. (2018). Radiographic, computed tomographic, gross pathological and histological findings with suspected apical infection in 32 equine maxillary cheek teeth (2012–2015). Equine Vet. J..

