# Supplementary material for: Selection of Filtering and Image Texture Analysis in the Radiographic Images Processing of Horses’ Incisor Teeth Affected by the EOTRH Syndrome
Source: Sensors (Basel). 2022 Apr 11;22(8):2920. doi: 10.3390/s22082920 (PMC9030967; doi:10.3390/s22082920)
Supplement: Supplementary file 1 [file sensors-22-02920-s001.zip › sensors-1639735-supplementary.pdf]

Supplementary Data for

Selection of Filtration and Image Texture Analysis in the Radiographic Images Processing of Horses' Incisor Teeth Affected by the EOTRH Syndrome

Kamil Górski [1], Marta Borowska [2], Elżbieta Stefanik [1], Izabela Polkowska [3], Bernard Turek [1], Andrzej Bereznowski [4] and Małgorzata Domino [1,*]

[1] Department of Large Animal Diseases and Clinic, Institute of Veterinary Medicine, Warsaw University of Life Sciences, 02-787 Warsaw, Poland; kamil_gorski@sggw.edu.pl (K.G.)
[2] Institute of Biomedical Engineering, Faculty of Mechanical Engineering, Białystok University of Technology, 15-351 Bialystok, Poland; m.borowska@pb.edu.pl (M.B.)
[3] Department and Clinic of Animal Surgery, Faculty of Veterinary Medicine, University of Life Sciences in Lublin, Poland (I.P.)
[4] Department of Veterinary Epidemiology and Economics, Faculty of Veterinary Medicine, Warsaw University of Life Sciences, Poland (A.B.)
* Correspondence: malgorzata_domino@sggw.edu.pl (M.D.)

Table S1. The values (mean ±SD) of features of *First Order Statistics* (FOS) of output images, filtrated by *Mean* filter, compared between four classes (0-3) of the Equine Odontoclastic Tooth Resorption and Hypercementosis (EOTRH) syndrome. When features differed significantly (p<0.05).

Feature		EOTRH	0	1	2	3
**Mean**		Mean	183.8	187.4	183.3	183.4
		SD	16.5	14.4	14.4	15.8
	p	0.0691	a	a	a	a
**Median**		Mean	183.6	189.8	185.9	186.7
		SD	16.2	15.0	15.3	16.7
	p	0.0134	a	b	ab	ab
**Minimum**		Mean	126.8	120.9	115.2	104.3
		SD	20.2	18.1	17.0	22.5
	p	< 0.0001	a	b	bc	c
**Maximum**		Mean	238.5	237.5	232.8	235.7
		SD	14.3	12.0	13.2	12.2
	p	0.0011	a	a	b	ab
**10th Percentile**		Mean	157.5	158.9	152.8	147.6
		SD	22.2	16.6	15.4	21.7
	p	0.0004	ab	a	bc	c
**90th Percentile**		Mean	209.6	211.3	208.9	213.5
		SD	13.7	13.9	14.2	14.0
	p	0.283	a	a	a	a
**Variance**		Mean	459.8	446.2	488.8	689.2
		SD	488.4	179.6	170.6	387.6
	p	< 0.0001	a	ab	b	c
**RMS**		Mean	185.1	188.6	184.6	185.3
		SD	15.2	14.2	14.3	15.2
	p	0.0544	a	a	a	a
**Kurtosis**		Mean	2.93	3.33	2.96	2.98
		SD	0.60	0.73	0.61	0.59
	p	< 0.0001	a	b	a	a
**Skewness**		Mean	0.02	-0.42	-0.43	-0.54
		SD	0.33	0.44	0.39	0.30
	p	< 0.0001	a	b	b	b
**Uniformity**		Mean	0.34	0.35	0.32	0.29
		SD	0.06	0.06	0.05	0.07
	p	< 0.0001	ab	a	bc	c
**Range**		Mean	111.7	116.6	117.6	131.5
		SD	21.3	16.7	17.5	25.2
	p	< 0.0001	a	b	b	c
**Interquartile Rang**		Mean	28.9	26.5	30.0	35.6
		SD	13.3	7.1	6.8	11.6
	p	< 0.0001	ab	a	bc	c
**MAD**		Mean	16.6	16.4	17.6	20.6
		SD	5.6	3.3	3.2	5.9
	p	< 0.0001	a	a	b	b
**rMAD**		Mean	12.0	11.3	12.7	15.0
		SD	4.9	2.8	2.7	4.8
	p	< 0.0001	a	a	b	b
**Energy**		Mean	81770000	65610000	76920000	70650000
		SD	126100000	40780000	50430000	33470000
	p	0.0029	a	b	a	a
**Total Energy**		Mean	81770000	65610000	76920000	70650000
		SD	126100000	40780000	50430000	33470000
	p	0.0029	a	b	a	a
**Entropy**		Mean	1.78	1.79	1.86	2.02
		SD	0.25	0.22	0.21	0.32
	p	< 0.0001	a	a	ab	b

RMS - root mean squared; MAD - mean absolute deviation; rMAD - robust mean absolute deviation. Lower case letters (a–c) indicate differences between classes for p < 0.05 independently for each feature.

Table S2. The values (mean ±SD) of features of *First Order Statistics* (FOS) of output images, filtrated by *Median* filter, compared between four classes (0-3) of the Equine Odontoclastic Tooth Resorption and Hypercementosis (EOTRH) syndrome. When features differed significantly (p<0.05).

Feature		EOTRH	0	1	2	3
**Mean**		Mean	184.4	188.3	184.1	184.1
		SD	16.5	14.5	14.5	15.9
	p	0.0507	a	a	a	a
**Median**		Mean	184.3	190.7	186.7	187.7
		SD	16.3	15.1	15.4	16.7
	p	0.0113	a	b	ab	ab
**Minimum**		Mean	125.8	120.8	114.9	104.2
		SD	20.3	18.4	16.9	23.2
	p	< 0.0001	a	b	bc	c
**Maximum**		Mean	241.2	239.7	235.6	238.8
		SD	14.6	12.1	13.5	12.5
	p	0.0018	a	ab	b	ab
**10th Percentile**		Mean	157.3	159.5	153.5	147.7
		SD	22.3	17.0	15.9	22.4
	p	0.0006	ab	a	bc	c
**90th Percentile**		Mean	210.9	212.3	210.1	214.6
		SD	13.5	13.9	14.4	14.1
	p	0.3422				
**Variance**		Mean	489.3	459.5	504.2	718.5
		SD	493.8	188.8	176.1	412.9
	p	< 0.0001	a	a	a	b
**RMS**		Mean	185.9	189.5	185.5	186.1
		SD	15.1	14.3	14.4	15.2
	p	0.0548				
**Kurtosis**		Mean	2.94	3.39	3.02	3.04
		SD	0.64	0.83	0.67	0.63
	p	< 0.0001	a	b	a	a
**Skewness**		Mean	0.03	-0.41	-0.41	-0.55
		SD	0.34	0.47	0.41	0.31
	p	< 0.0001	a	b	bc	c
**Uniformity**		Mean	0.33	0.35	0.32	0.29
		SD	0.06	0.06	0.05	0.07
	p	< 0.0001	ab	a	bc	c
**Range**		Mean	115.4	118.9	120.6	134.6
		SD	21.9	17.3	17.5	27.0
	p	< 0.0001	a	a	a	b
**Interquartile Rang**		Mean	30.0	26.8	30.5	36.1
		SD	13.4	7.3	7.1	12.5
	p	< 0.0001	ab	a	bc	c
**MAD**		Mean	17.2	16.6	17.9	21.0
		SD	5.6	3.4	3.3	6.2
	p	< 0.0001	ab	a	bc	c
**rMAD**		Mean	12.5	11.5	12.8	15.3
		SD	4.9	2.9	2.8	5.0
	p	< 0.0001	ab	a	bc	c
**Energy**		Mean	82490000	66230000	77530000	71240000
		SD	128000000	41010000	50650000	33670000
	p	0.0034	ab	a	b	b
**Total Energy**		Mean	82490000	66230000	77530000	71240000
		SD	128000000	41010000	50650000	33670000
	p	0.0034	ab	a	b	b
**Entropy**		Mean	1.82	1.81	1.88	2.04
		SD	0.25	0.22	0.21	0.33
	p	< 0.0001	ab	a	bc	c

RMS - root mean squared; MAD - mean absolute deviation; rMAD - robust mean absolute deviation. Lower case letters (a–c) indicate differences between classes for p < 0.05 independently for each feature.

Table S3. The values (mean ±SD) of features of *First Order Statistics* (FOS) of output images, filtrated by *Normalize* filter, compared between four classes (0-3) of the Equine Odontoclastic Tooth Resorption and Hypercementosis (EOTRH) syndrome. When features differed significantly (p<0.05).

Feature		EOTRH	0	1	2	3
**Mean**		Mean	1.28	1.31	1.25	1.21
		SD	0.18	0.20	0.17	0.19
	p	< 0.0001	ab	a	bc	c
**Median**		Mean	1.28	1.35	1.29	1.26
		SD	0.18	0.20	0.18	0.19
	p	< 0.0001	a	b	a	a
**Minimum**		Mean	0.27	0.19	0.06	-0.16
		SD	0.26	0.30	0.30	0.42
	p	< 0.0001	a	a	b	c
**Maximum**		Mean	2.21	2.20	2.17	2.14
		SD	0.13	0.18	0.17	0.20
	p	0.1612	a	a	a	a
**10th Percentile**		Mean	0.84	0.86	0.76	0.64
		SD	0.28	0.25	0.21	0.32
	p	< 0.0001	a	a	b	b
**90th Percentile**		Mean	1.70	1.70	1.67	1.69
		SD	0.11	0.18	0.14	0.16
	p	0.0764	a	a	a	a
**Variance**		Mean	0.12	0.12	0.13	0.18
		SD	0.09	0.05	0.04	0.11
	p	< 0.0001	a	a	b	b
**RMS**		Mean	1.33	1.36	1.30	1.28
		SD	0.12	0.18	0.15	0.16
	p	0.0622	a	a	a	a
**Kurtosis**		Mean	2.96	3.40	3.11	3.10
		SD	0.57	0.74	0.61	0.58
	p	< 0.0001	a	b	a	a
**Skewness**		Mean	0.01	-0.37	-0.38	-0.53
		SD	0.29	0.43	0.39	0.28
	p	< 0.0001	a	b	b	c
**Uniformity**		Mean	1.00	1.00	1.00	0.97
		SD	0.05	0.03	0.01	0.05
	p	< 0.0001	a	b	b	c
**Range**		Mean	1.94	2.01	2.11	2.30
		SD	0.31	0.30	0.30	0.49
	p	< 0.0001	a	ab	b	b
**Interquartile Rang**		Mean	0.47	0.43	0.49	0.57
		SD	0.18	0.11	0.10	0.20
	p	< 0.0001	ab	a	bc	c
**MAD**		Mean	0.27	0.26	0.29	0.33
		SD	0.08	0.05	0.05	0.10
	p	< 0.0001	a	a	b	b
**rMAD**		Mean	0.20	0.18	0.20	0.24
		SD	0.07	0.04	0.04	0.08
	p	< 0.0001	a	a	ab	b
**Energy**		Mean	4427	3267	3662	3330
		SD	9343	1781	2006	1417
	p	0.0599				
**Total Energy**		Mean	4427	3267	3662	3330
		SD	9343	1781	2006	1417
	p	0.0599	a	a	a	a
**Entropy**		Mean	0.01	0.02	0.02	0.09
		SD	0.10	0.07	0.04	0.14
	p	< 0.0001	a	b	b	c

RMS - root mean squared; MAD - mean absolute deviation; rMAD - robust mean absolute deviation. Lower case letters (a–c) indicate differences between classes for p < 0.05 independently for each feature.

Table S4. The values (mean ±SD) of features of *First Order Statistics* (FOS) of output images, filtrated by *Bilateral* filter, compared between four classes (0-3) of the Equine Odontoclastic Tooth Resorption and Hypercementosis (EOTRH) syndrome. When features differed significantly (p<0.05).

Feature		EOTRH	0	1	2	3
**Mean**		Mean	182.1	184.2	180.7	181.5
		SD	13.9	14.2	14.3	15.7
	p	0.1226	a	a	a	a
**Median**		Mean	181.3	186.0	182.9	184.3
		SD	14.2	14.8	15.0	16.9
	p	0.0578	a	a	a	a
**Minimum**		Mean	141.6	130.8	125.4	117.1
		SD	14.56	16.19	15.5	19.06
	p	< 0.0001	a	b	bc	c
**Maximum**		Mean	218.4	218.3	214.8	219.0
		SD	14.9	12.6	13.4	12.6
	p	0.0386	a	ab	b	ab
**10th Percentile**		Mean	163.8	161.7	156.8	152.1
		SD	14.9	16.2	15.0	20.0
	p	0.0001	a	ab	bc	c
**90th Percentile**		Mean	202.9	204.4	201.5	206.1
		SD	14.3	14.3	14.9	14.2
	p	0.2658	a	a	a	a
**Variance**		Mean	231.7	285.6	308.4	464.2
		SD	103.7	135.3	130.8	279.6
	p	< 0.0001	a	b	b	c
**RMS**		Mean	182.8	185.0	181.6	182.8
		SD	13.8	14.1	14.3	15.3
	p	0.1445	a	a	a	a
**Kurtosis**		Mean	3.04	3.44	3.13	3.12
		SD	0.69	0.92	0.83	0.87
	p	< 0.0001	a	b	a	a
**Skewness**		Mean	0.15	-0.45	-0.47	-0.60
		SD	0.44	0.55	0.48	0.44
	p	< 0.0001	a	b	b	b
**Uniformity**		Mean	0.44	0.42	0.40	0.36
		SD	0.09	0.08	0.07	0.08
	p	< 0.0001	a	ab	b	c
**Range**		Mean	76.8	87.5	89.4	101.8
		SD	14.4	15.2	16.2	18.8
	p	< 0.0001	a	b	b	c
**Interquartile Rang**		Mean	19.2	20.1	23.0	29.2
		SD	6.6	6.7	6.7	10.9
	p	< 0.0001	a	b	c	d
**MAD**		Mean	11.8	12.9	13.8	16.9
		SD	2.9	3.1	3.1	5.2
	p	< 0.0001	a	b	b	c
**rMAD**		Mean	8.32	8.78	9.88	12.30
		SD	2.61	2.62	2.59	4.37
	p	< 0.0001	a	a	b	c
**Energy**		Mean	68010000	63340000	74660000	68780000
		SD	38490000	40020000	49660000	32860000
	p	0.0022	a	b	a	a
**Total Energy**		Mean	68010000	63340000	74660000	68780000
		SD	38490000	40020000	49660000	32860000
	p	0.0022	a	b	a	a
**Entropy**		Mean	1.41	1.51	1.55	1.73
		SD	0.24	0.25	0.24	0.32
	p	< 0.0001	a	b	b	c

RMS - root mean squared; MAD - mean absolute deviation; rMAD - robust mean absolute deviation. Lower case letters (a–c) indicate differences between classes for p < 0.05 independently for each feature.

Table S5. The values (mean ±SD) of features of *First Order Statistics* (FOS) of output images, filtrated by *Binomial* filter, compared between four classes (0-3) of the Equine Odontoclastic Tooth Resorption and Hypercementosis (EOTRH) syndrome. When features differed significantly (p<0.05).

Feature		EOTRH	0	1	2	3
**Mean**		Mean	184.7	187.5	183.4	183.5
		SD	13.73	14.39	14.42	15.8
	p	0.0576	a	a	a	a
**Median**		Mean	184.6	189.9	186.1	186.9
		SD	13.58	14.92	15.33	16.56
	p	0.0175	a	b	ab	ab
**Minimum**		Mean	126.5	120.3	114	102.7
		SD	16.23	18.22	17.5	24.16
	p	< 0.0001	a	b	bc	c
**Maximum**		Mean	239.9	239	234.5	237.3
		SD	13.8	11.68	13	12.1
	p	0.0014	a	a	b	ab
**10th Percentile**		Mean	158.6	158.7	152.7	147.3
		SD	16.49	16.67	15.54	21.97
	p	0.0003	ab	a	bc	c
**90th Percentile**		Mean	210.3	211.6	209.3	213.9
		SD	13.6	13.88	14.2	13.96
	p	0.3007	a	a	a	a
**Variance**		Mean	430.6	456.1	500.7	709.2
		SD	161.5	183.6	173	402.6
	p	< 0.0001	a	ab	b	c
**RMS**		Mean	185.9	188.7	184.8	185.4
		SD	13.53	14.2	14.29	15.17
	p	0.054	a	a	a	a
**Kurtosis**		Mean	2.94	3.326	2.974	2.993
		SD	0.5889	0.7297	0.597	0.5913
	p	< 0.0001	a	b	a	a
**Skewness**		Mean	0.0197	-0.4008	-0.4153	-0.538
		SD	0.323	0.4414	0.3854	0.297
	p	< 0.0001	a	b	bc	c
**Uniformity**		Mean	0.3366	0.346	0.3185	0.29
		SD	0.05577	0.06035	0.05026	0.06667
	p	< 0.0001	ab	a	bc	c
**Range**		Mean	113.4	118.7	120.5	134.6
		SD	16.54	16.84	17.97	27.22
	p	< 0.0001	a	b	b	c
**Interquartile Rang**		Mean	28.4	26.82	30.34	36.09
		SD	7.355	7.161	6.73	12.03
	p	< 0.0001	ab	a	bc	c
**MAD**		Mean	16.47	16.57	17.83	20.91
		SD	3.377	3.351	3.213	6.056
	p	< 0.0001	a	a	b	b
**rMAD**		Mean	11.86	11.46	12.8	15.19
		SD	2.917	2.835	2.688	4.909
	p	< 0.0001	a	a	b	b
**Energy**		Mean	70140000	65710000	77000000	70740000
		SD	38980000	40800000	50420000	33470000
	p	0.0035	ab	a	b	b
**Total Energy**		Mean	70140000	65710000	77000000	70740000
		SD	38980000	40800000	50420000	33470000
	p	0.0035	ab	a	bc	c
**Entropy**		Mean	1.797	1.804	1.879	2.033
		SD	0.2208	0.2201	0.2094	0.3277
	p	< 0.0001	ab	a	bc	c

RMS - root mean squared; MAD - mean absolute deviation; rMAD - robust mean absolute deviation. Lower case letters (a–c) indicate differences between classes for p < 0.05 independently for each feature.

Table S6. The values (mean ±SD) of features of *First Order Statistics* (FOS) of output images, filtrated by *CurvatureFlow* filter, compared between four classes (0-3) of the Equine Odontoclastic Tooth Resorption and Hypercementosis (EOTRH) syndrome. When features differed significantly (p<0.05).

Feature		EOTRH	0	1	2	3
**Mean**		Mean	185.5	188.4	184.2	184.2
		SD	13.7	14.5	14.5	15.8
	p	0.0713	a	a	a	a
**Median**		Mean	185.4	190.7	186.8	187.9
		SD	13.6	15.1	15.4	16.5
	p	0.0191	a	b	ab	ab
**Minimum**		Mean	121.9	116.8	109.9	97.8
		SD	16.5	18.3	18.6	26.0
	p	< 0.0001	a	a	b	b
**Maximum**		Mean	245.3	244.7	241.7	244.1
		SD	12.6	10.7	13.0	11.7
	p	0.0367	a	ab	b	ab
**10th Percentile**		Mean	157.7	158.7	152.6	146.8
		SD	16.7	17.0	15.8	22.6
	p	0.0006	ab	a	bc	c
**90th Percentile**		Mean	212.5	213.4	211.1	215.7
		SD	13.3	14.0	14.3	13.9
	p	0.3104	a	a	a	a
**Variance**		Mean	482.9	493.9	543.9	771.9
		SD	176.7	195.7	181.9	446.6
	p	< 0.0001	a	a	ab	b
**RMS**		Mean	186.8	189.7	185.7	186.4
		SD	13.5	14.3	14.4	15.1
	p	0.0685				
**Kurtosis**		Mean	2.95	3.39	3.08	3.07
		SD	0.59	0.78	0.64	0.61
	p	< 0.0001	a	b	a	a
**Skewness**		Mean	0.02	-0.38	-0.38	-0.53
		SD	0.31	0.45	0.40	0.30
	p	< 0.0001	a	b	b	c
**Uniformity**		Mean	0.32	0.33	0.31	0.28
		SD	0.05	0.06	0.05	0.07
	p	< 0.0001	a	a	b	b
**Range**		Mean	123.4	128.0	131.8	146.3
		SD	16.7	17.9	19.3	29.7
	p	< 0.0001	a	ab	bc	c
**Interquartile Rang**		Mean	30.2	27.9	31.5	37.4
		SD	7.5	7.2	6.8	12.9
	p	< 0.0001	ab	a	bc	c
**MAD**		Mean	17.5	17.2	18.5	21.7
		SD	3.5	3.4	3.2	6.5
	p	< 0.0001	ab	a	bc	c
**rMAD**		Mean	12.4	11.8	13.1	15.6
		SD	3.0	2.9	2.7	5.2
	p	< 0.0001	a	a	b	b
**Energy**		Mean	70730000	66360000	77690000	71370000
		SD	39140000	41070000	50720000	33620000
	p	0.0037	ab	a	b	b
**Total Energy**		Mean	70730000	66360000	77690000	71370000
		SD	39140000	41070000	50720000	33620000
	p	0.0037	ab	a	b	b
**Entropy**		Mean	1.87	1.86	1.94	2.09
		SD	0.22	0.22	0.20	0.34
	p	< 0.0001	ab	a	bc	c

RMS - root mean squared; MAD - mean absolute deviation; rMAD - robust mean absolute deviation. Lower case letters (a–c) indicate differences between classes for p < 0.05 independently for each feature.

Table S7. The values (mean ±SD) of features of *First Order Statistics* (FOS) of output images, filtrated by *LaplacianSharpening* filter, compared between four classes (0-3) of the Equine Odontoclastic Tooth Resorption and Hypercementosis (EOTRH) syndrome. When features differed significantly (p<0.05).

Feature		EOTRH	0	1	2	3
**Mean**		Mean	185.3	188.8	184.3	184.0
		SD	13.5	14.5	14.5	15.8
	p	0.0542	a	a	a	a
**Median**		Mean	185.7	191.0	186.9	188.3
		SD	13.6	15.3	15.5	16.1
	p	0.0166	a	b	ab	ab
**Minimum**		Mean	72.3	65.6	56.9	39.5
		SD	22.6	27.5	25.7	33.2
	p	< 0.0001	a	ab	b	c
**Maximum**		Mean	254.4	254.6	254.5	254.9
		SD	2.1	2.6	2.2	0.9
	p	0.1799	a	a	a	a
**10th Percentile**		Mean	133.2	148.5	141.3	133.2
		SD	27.3	17.8	17.3	27.3
	p	< 0.0001	a	b	a	a
**90th Percentile**		Mean	225.6	226.6	223.6	228.7
		SD	13.0	14.0	13.8	14.3
	p	0.156	a	a	a	a
**Variance**		Mean	992.6	979.5	1073.0	1496.0
		SD	316.6	304.7	311.4	870.2
	p	< 0.0001	a	a	ab	b
**RMS**		Mean	188.0	191.4	187.2	188.1
		SD	12.9	14.1	14.1	14.4
	p	0.0331	ab	a	b	b
**Kurtosis**		Mean	3.05	3.53	3.38	3.39
		SD	0.40	0.77	0.70	0.66
	p	< 0.0001	a	b	b	b
**Skewness**		Mean	-0.15	-0.44	-0.43	-0.56
		SD	0.22	0.35	0.34	0.27
	p	< 0.0001	a	b	b	c
**Uniformity**		Mean	0.23	0.23	0.22	0.20
		SD	0.04	0.03	0.03	0.05
	p	0.0001	ab	a	b	b
**Range**		Mean	182.1	189.0	197.6	215.3
		SD	22.2	27.5	25.9	33.4
	p	< 0.0001	a	ab	b	c
**Interquartile Rang**		Mean	42.0	39.7	42.6	50.2
		SD	8.3	7.6	7.4	17.0
	p	< 0.0001	ab	a	b	b
**MAD**		Mean	24.9	24.3	25.7	29.8
		SD	4.2	3.9	3.9	8.8
	p	< 0.0001	ab	a	bc	c
**rMAD**		Mean	17.6	16.8	17.9	21.1
		SD	3.3	3.2	3.0	6.8
	p	< 0.0001	ab	b	bc	a
**Energy**		Mean	71520000	67450000	78860000	72530000
		SD	39140000	41520000	51080000	33550000
	p	0.0036	ab	a	b	b
**Total Energy**		Mean	71520000	67450000	78860000	72530000
		SD	39140000	41520000	51080000	33550000
	p	0.0036	ab	a	b	b
**Entropy**		Mean	2.38	2.35	2.42	2.56
		SD	0.21	0.19	0.19	0.34
	p	0.0004	a	a	ab	b

RMS - root mean squared; MAD - mean absolute deviation; rMAD - robust mean absolute deviation. Lower case letters (a–c) indicate differences between classes for p < 0.05 independently for each feature.

Table S8. The values (mean ±SD) of features of *First Order Statistics* (FOS) of output images, filtrated by *DiscreteGaussian* filter, compared between four classes (0-3) of the Equine Odontoclastic Tooth Resorption and Hypercementosis (EOTRH) syndrome. When features differed significantly (p<0.05).

Feature		EOTRH	0	1	2	3
**Mean**		Mean	183.9	186.6	182.5	182.7
		SD	13.8	14.3	14.4	15.8
	p	0.0531	a	a	a	a
**Median**		Mean	183.7	189.0	185.2	186.0
		SD	13.6	14.9	15.3	16.8
	p	0.0173	a	b	ab	ab
**Minimum**		Mean	128.6	121.6	116.0	105.1
		SD	15.9	17.7	17.0	22.1
	p	< 0.0001	a	b	bc	c
**Maximum**		Mean	236.2	235.3	230.6	233.6
		SD	14.4	12.0	12.9	12.4
	p	0.0011	ab	a	b	ab
**10th Percentile**		Mean	158.8	158.3	152.4	147.3
		SD	16.3	16.5	15.4	21.5
	p	0.0002	a	a	b	b
**90th Percentile**		Mean	208.6	210.2	207.9	212.4
		SD	13.8	13.8	14.3	14.0
	p	0.3083	a	a	a	a
**Variance**		Mean	398.4	435.6	477.2	670.8
		SD	151.5	175.3	167.8	372.6
	p	< 0.0001	a	ab	b	c
**RMS**		Mean	185.0	187.8	183.8	184.6
		SD	13.6	14.2	14.2	15.2
	p	0.0595	a	a	a	a
**Kurtosis**		Mean	2.95	3.31	2.93	2.97
		SD	0.60	0.71	0.59	0.60
	p	< 0.0001	a	b	a	a
**Skewness**		Mean	0.02	-0.42	-0.43	-0.54
		SD	0.33	0.44	0.38	0.31
	p	< 0.0001	a	b	b	b
**Uniformity**		Mean	0.35	0.35	0.33	0.30
		SD	0.06	0.06	0.05	0.07
	p	< 0.0001	a	a	b	b
**Range**		Mean	107.6	113.7	114.6	128.5
		SD	16.2	16.1	16.9	24.4
	p	< 0.0001	a	b	b	c
**Interquartile Rang**		Mean	27.1	26.1	29.8	35.2
		SD	7.3	7.1	6.7	11.6
	p	< 0.0001	a	a	b	b
**MAD**		Mean	15.8	16.2	17.4	20.4
		SD	3.3	3.3	3.2	5.8
	p	< 0.0001	a	a	b	b
**rMAD**		Mean	11.4	11.2	12.6	14.8
		SD	2.9	2.8	2.7	4.7
	p	< 0.0001	a	a	b	b
**Energy**		Mean	69490000	65050000	76290000	70100000
		SD	38700000	40490000	50070000	33300000
	p	0.0034	ab	a	b	b
**Total Energy**		Mean	69490000	65050000	76290000	70100000
		SD	38700000	40490000	50070000	33300000
	p	0.0034	ab	a	b	b
**Entropy**		Mean	1.75	1.77	1.84	2.00
		SD	0.22	0.22	0.21	0.32
	p	< 0.0001	a	ab	bc	c

RMS - root mean squared; MAD - mean absolute deviation; rMAD - robust mean absolute deviation. Lower case letters (a–c) indicate differences between classes for p < 0.05 independently for each feature.

Table S9. The values (mean ±SD) of features of *First Order Statistics* (FOS) of output images, filtrated by *SmoothingRecursiveGaussian* filter, compared between four classes (0-3) of the Equine Odontoclastic Tooth Resorption and Hypercementosis (EOTRH) syndrome. When features differed significantly (p<0.05).

Feature		EOTRH	0	1	2	3
**Mean**		Mean	184.9	187.6	183.5	183.7
		SD	13.8	14.3	14.4	15.8
	p	0.0637	a	a	a	a
**Median**		Mean	184.8	190.0	186.2	187.0
		SD	13.6	14.9	15.3	16.9
	p	0.0184	a	b	ab	ab
**Minimum**		Mean	129.8	122.7	117.3	106.6
		SD	15.8	17.7	16.9	21.7
	p	< 0.0001	a	b	b	c
**Maximum**		Mean	236.8	235.9	231.1	234.3
		SD	14.6	12.1	13.0	12.5
	p	0.0012	a	a	b	ab
**10th Percentile**		Mean	159.9	159.3	153.4	148.4
		SD	16.2	16.5	15.3	21.5
	p	0.0002	a	a	b	b
**90th Percentile**		Mean	209.5	211.2	208.8	213.4
		SD	13.9	13.9	14.3	14.0
	p	0.2983	a	a	a	a
**Variance**		Mean	396.0	434.4	475.6	667.7
		SD	151.1	175.0	167.8	370.5
	p	< 0.0001	a	ab	b	c
**RMS**		Mean	186.0	188.7	184.8	185.5
		SD	13.6	14.2	14.2	15.2
	p	0.0596	a	a	a	a
**Kurtosis**		Mean	2.94	3.31	2.93	2.97
		SD	0.60	0.71	0.60	0.60
	p	< 0.0001	a	b	a	a
**Skewness**		Mean	0.02	-0.43	-0.43	-0.54
		SD	0.34	0.44	0.39	0.31
	p	< 0.0001	a	b	b	b
**Uniformity**		Mean	0.35	0.35	0.33	0.30
		SD	0.06	0.06	0.05	0.07
	p	< 0.0001	a	a	b	b
**Range**		Mean	106.9	113.3	113.8	127.7
		SD	16.2	16.2	16.9	23.9
	p	< 0.0001	a	b	b	c
**Interquartile Rang**		Mean	27.1	26.1	29.8	35.1
		SD	7.3	7.0	6.8	11.4
	p	< 0.0001	a	a	b	b
**MAD**		Mean	15.8	16.2	17.4	20.4
		SD	3.3	3.3	3.2	5.7
	p	< 0.0001	a	a	b	b
**rMAD**		Mean	11.2	11.0	12.4	14.6
		SD	2.8	2.8	2.7	4.7
	p	< 0.0001	a	a	b	b
**Energy**		Mean	70230000	65750000	77100000	70820000
		SD	39090000	40950000	50590000	33560000
	p	0.0032	ab	a	b	b
**Total Energy**		Mean	70230000	65750000	77090000	70820000
		SD	39090000	40950000	50560000	33560000
	p	0.0032	ab	a	b	b
**Entropy**		Mean	1.74	1.77	1.84	2.00
		SD	0.22	0.22	0.21	0.32
	p	< 0.0001	a	ab	bc	c

RMS - root mean squared; MAD - mean absolute deviation; rMAD - robust mean absolute deviation. Lower case letters (a–c) indicate differences between classes for p < 0.05 independently for each feature.

Table S10. The values (mean ±SD) of features of *Gray Level Co-occurence Matrix (GLCM)* of output images, filtrated by *Mean* filter, compared between four classes (0-3) of the Equine Odontoclastic Tooth Resorption and Hypercementosis (EOTRH) syndrome. When features differed significantly (p<0.05).

Feature		EOTRH	0	1	2	3	Feature		EOTRH	0	1	2	3
**Autocorrelation**		Mean	11.4	14.2	15.3	18.7	**IDN**		Mean	0.90	0.90	0.90	0.90
		SD	3.9	4.4	4.8	6.1			SD	0.02	0.01	0.02	0.02
	p	< 0.0001	a	b	b	c		p	0.0575	a	a	a	a
**Cluster Prominence**		Mean	26.9	13.3	15.9	38.1	**IMC 1**		Mean	-0.11	-0.11	-0.11	-0.11
		SD	146.8	17.4	12.8	47.6			SD	0.05	0.03	0.04	0.04
	p	< 0.0001	a	a	a	b		p	0.5883	a	a	a	a
**Cluster Shade**		Mean	0.77	-0.59	-1.06	-2.27	**IMC 2**		Mean	0.53	0.50	0.52	0.54
		SD	2.98	1.20	1.39	2.73			SD	0.10	0.09	0.10	0.10
	p	< 0.0001	a	b	c	d		p	0.0584	a	a	a	a
**Cluster Tendency**		Mean	2.23	1.87	2.20	3.26	**Inverse Variance**		Mean	0.47	0.46	0.48	0.47
		SD	2.82	0.93	0.90	2.10			SD	0.05	0.04	0.04	0.04
	p	< 0.0001	ab	a	b	c		p	0.0126	ab	a	b	ab
**Contrast**		Mean	0.90	1.00	1.06	1.35	**Joint Average**		Mean	3.28	3.70	3.83	4.22
		SD	0.28	0.32	0.34	0.71			SD	0.54	0.58	0.61	0.68
	p	< 0.0001	a	b	bc	c		p	< 0.0001	a	b	b	c
**Correlation**		Mean	0.36	0.28	0.33	0.39	**Joint Energy**		Mean	0.15	0.16	0.13	0.12
		SD	0.12	0.14	0.15	0.13			SD	0.05	0.06	0.04	0.06
	p	< 0.0001	a	b	a	a		p	< 0.0001	ab	a	bc	c
**Difference Average**		Mean	0.66	0.69	0.72	0.81	**Joint Entropy**		Mean	3.28	3.23	3.40	3.73
		SD	0.13	0.13	0.13	0.25			SD	0.45	0.42	0.39	0.69
	p	0.0002	a	ab	bc	c		p	< 0.0001	ab	a	bc	c
**Difference Entropy**		Mean	1.35	1.41	1.43	1.53	**MCC**		Mean	0.46	0.42	0.43	0.45
		SD	0.14	0.14	0.15	0.26			SD	0.11	0.10	0.10	0.11
	p	< 0.0001	a	b	b	b		p	0.0155	a	b	ab	ab
**Difference Variance**		Mean	0.43	0.49	0.49	0.60	**Maximum Probability**		Mean	0.25	0.29	0.24	0.22
		SD	0.10	0.12	0.13	0.25			SD	0.08	0.10	0.07	0.10
	p	< 0.0001	a	b	b	b		p	< 0.0001	a	b	a	a
**ID**		Mean	0.71	0.70	0.69	0.67	**Sum Average**		Mean	6.56	7.40	7.66	8.44
		SD	0.04	0.04	0.04	0.07			SD	1.08	1.15	1.21	1.37
	p	0.0003	a	ab	bc	c		p	< 0.0001	a	b	b	c
**IDM**		Mean	0.70	0.69	0.67	0.65	**Sum Entropy**		Mean	2.40	2.34	2.47	2.67
		SD	0.05	0.05	0.05	0.08			SD	0.31	0.28	0.27	0.39
	p	0.0003	a	ab	bc	c		p	< 0.0001	ab	a	bc	c
**IDMN**		Mean	0.97	0.97	0.97	0.97	**Sum Squares**		Mean	0.78	0.72	0.81	1.15
		SD	0.01	0.01	0.01	0.01			SD	0.73	0.29	0.27	0.68
	p	0.3501	a	a	a	a		p	< 0.0001	a	a	b	b

ID - inverse difference; IDM - inverse difference moment; IDMN - inverse difference moment normalized; IDN - inverse difference normalized; IMC 1 - informational measure of correlation 1; IMC 2 - informational measure of correlation 2; MCC - maximal correlation coefficient. Lower case letters (a–d) indicate differences between classes for p < 0.05 independently for each feature.

Table S11. The values (mean ±SD) of features of *Gray Level Co-occurence Matrix (GLCM)* of output images, filtrated by *Median* filter, compared between four classes (0-3) of the Equine Odontoclastic Tooth Resorption and Hypercementosis (EOTRH) syndrome. When features differed significantly (p<0.05).

Feature		EOTRH	0	1	2	3	Feature		EOTRH	0	1	2	3
**Autocorrelation**		Mean	11.5	14.7	15.5	19.1	**IDN**		Mean	0.90	0.90	0.90	0.90
		SD	3.6	4.4	4.8	6.8			SD	0.02	0.01	0.02	0.02
	p	< 0.0001	a	b	b	c		p	0.1643	a	a	a	a
**Cluster Prominence**		Mean	14.0	14.2	16.8	41.1	**IMC 1**		Mean	-0.10	-0.10	-0.10	-0.10
		SD	11.1	19.2	13.9	52.0			SD	0.03	0.03	0.04	0.04
	p	< 0.0001	a	a	a	b		p	0.8765	a	a	a	a
**Cluster Shade**		Mean	0.55	-0.56	-1.05	-2.47	**IMC 2**		Mean	0.51	0.50	0.51	0.53
		SD	1.07	1.27	1.41	2.86			SD	0.09	0.09	0.10	0.10
	p	< 0.0001	a	b	c	d		p	0.0839	a	a	a	a
**Cluster Tendency**		Mean	2.06	1.93	2.24	3.36	**Inverse Variance**		Mean	0.48	0.47	0.48	0.47
		SD	0.89	0.98	0.93	2.19			SD	0.04	0.04	0.04	0.04
	p	< 0.0001	a	a	a	b		p	0.0682	a	a	a	a
**Contrast**		Mean	0.99	1.05	1.11	1.43	**Joint Average**		Mean	3.32	3.76	3.85	4.25
		SD	0.33	0.34	0.35	0.80			SD	0.52	0.56	0.62	0.74
	p	0.0009	a	ab	b	b		p	< 0.0001	a	b	b	c
**Correlation**		Mean	0.33	0.27	0.31	0.38	**Joint Energy**		Mean	0.14	0.16	0.13	0.11
		SD	0.11	0.14	0.15	0.12			SD	0.05	0.05	0.04	0.05
	p	< 0.0001	ac	b	a	c		p	< 0.0001	a	b	ac	bc
**Difference Average**		Mean	0.69	0.71	0.74	0.84	**Joint Entropy**		Mean	3.35	3.28	3.46	3.78
		SD	0.14	0.14	0.14	0.27			SD	0.45	0.43	0.40	0.71
	p	0.0037	a	a	ab	b		p	< 0.0001	ab	a	bc	c
**Difference Entropy**		Mean	1.39	1.43	1.46	1.56	**MCC**		Mean	0.44	0.41	0.42	0.44
		SD	0.15	0.15	0.15	0.27			SD	0.09	0.09	0.10	0.11
	p	0.0002	a	ab	b	b		p	0.061	a	a	a	a
**Difference Variance**		Mean	0.46	0.51	0.52	0.64	**Maximum Probability**		Mean	0.25	0.28	0.24	0.21
		SD	0.12	0.13	0.13	0.28			SD	0.08	0.09	0.07	0.10
	p	< 0.0001	a	b	b	b		p	< 0.0001	a	b	a	a
**ID**		Mean	0.70	0.70	0.68	0.66	**Sum Average**		Mean	6.64	7.52	7.70	8.49
		SD	0.05	0.04	0.04	0.07			SD	1.04	1.13	1.23	1.48
	p	0.0041	a	a	ab	b		p	< 0.0001	a	b	b	c
**IDM**		Mean	0.68	0.68	0.67	0.64	**Sum Entropy**		Mean	2.42	2.36	2.49	2.69
		SD	0.05	0.05	0.05	0.09			SD	0.29	0.28	0.27	0.40
	p	0.0044	a	a	ab	b		p	< 0.0001	ab	a	bc	c
**IDMN**		Mean	0.97	0.97	0.97	0.97	**Sum Squares**		Mean	0.76	0.74	0.84	1.20
		SD	0.01	0.01	0.01	0.01			SD	0.29	0.31	0.28	0.72
	p	0.5074	a	a	a	a		p	< 0.0001	ab	a	bc	c

ID - inverse difference; IDM - inverse difference moment; IDMN - inverse difference moment normalized; IDN - inverse difference normalized; IMC 1 - informational measure of correlation 1; IMC 2 - informational measure of correlation 2; MCC - maximal correlation coefficient. Lower case letters (a–d) indicate differences between classes for p < 0.05 independently for each feature.

Table S12. The values (mean ±SD) of features of *Gray Level Co-occurence Matrix (GLCM)* of output images, filtrated by *Normalize* filter, compared between four classes (0-3) of the Equine Odontoclastic Tooth Resorption and Hypercementosis (EOTRH) syndrome. When features differed significantly (p<0.05).

Feature		EOTRH	0	1	2	3	Feature		EOTRH	0	1	2	3
**Autocorrelation**		Mean	1.27	1.73	2.13	2.71	**IDN**		Mean	1.00	1.00	1.00	0.99
		SD	0.85	1.28	1.45	1.44			SD	0.00	0.01	0.00	0.02
	p	< 0.0001	a	b	bc	c		p	< 0.0001	a	b	b	c
**Cluster Prominence**		Mean	0.01	0.01	0.01	0.05	**IMC 1**		Mean	-0.01	0.00	0.00	-0.01
		SD	0.02	0.05	0.09	0.09			SD	0.05	0.01	0.01	0.01
	p	< 0.0001	a	b	b	c		p	< 0.0001	a	b	b	c
**Cluster Shade**		Mean	0.00	0.00	0.00	-0.03	**IMC 2**		Mean	0.01	0.01	0.01	0.03
		SD	0.00	0.03	0.01	0.06			SD	0.08	0.03	0.02	0.05
	p	< 0.0001	a	b	b	c		p	< 0.0001	a	b	b	c
**Cluster Tendency**		Mean	0.01	0.01	0.00	0.03	**Inverse Variance**		Mean	0.00	0.00	0.00	0.03
		SD	0.09	0.04	0.01	0.06			SD	0.01	0.02	0.01	0.05
	p	< 0.0001	a	ab	b	c		p	< 0.0001	a	b	b	c
**Contrast**		Mean	0.00	0.00	0.00	0.03	**Joint Average**		Mean	1.09	1.24	1.38	1.57
		SD	0.01	0.02	0.01	0.05			SD	0.28	0.43	0.48	0.48
	p	< 0.0001	a	b	b	c		p	< 0.0001	a	b	bc	c
**Correlation**		Mean	0.91	0.76	0.63	0.45	**Joint Energy**		Mean	0.99	0.99	0.99	0.94
		SD	0.28	0.42	0.48	0.47			SD	0.06	0.05	0.02	0.10
	p	< 0.0001	a	b	bc	c		p	< 0.0001	a	ab	b	c
**Difference Average**		Mean	0.00	0.00	0.00	0.03	**Joint Entropy**		Mean	0.02	0.03	0.03	0.18
		SD	0.01	0.02	0.01	0.05			SD	0.14	0.13	0.08	0.29
	p	< 0.0001	a	b	b	c		p	< 0.0001	a	ab	b	c
**Difference Entropy**		Mean	0.01	0.02	0.03	0.13	**MCC**		Mean	0.91	0.76	0.63	0.44
		SD	0.04	0.07	0.06	0.20			SD	0.28	0.42	0.48	0.47
	p	< 0.0001	a	b	b	c		p	< 0.0001	a	b	bc	c
**Difference Variance**		Mean	0.00	0.00	0.00	0.02	**Maximum Probability**		Mean	0.99	1.00	1.00	0.97
		SD	0.01	0.02	0.01	0.04			SD	0.05	0.03	0.01	0.06
	p	< 0.0001	a	b	b	c		p	< 0.0001	a	b	b	c
**ID**		Mean	1.00	1.00	1.00	0.99	**Sum Average**		Mean	2.18	2.49	2.75	3.15
		SD	0.00	0.01	0.00	0.02			SD	0.57	0.86	0.97	0.97
	p	< 0.0001	a	b	b	c		p	< 0.0001	a	b	bc	c
**IDM**		Mean	1.00	1.00	1.00	0.99	**Sum Entropy**		Mean	0.02	0.02	0.03	0.15
		SD	0.00	0.01	0.00	0.02			SD	0.13	0.11	0.07	0.24
	p	< 0.0001	a	b	b	c		p	< 0.0001	a	ab	b	c
**IDMN**		Mean	1.00	1.00	1.00	0.99	**Sum Squares**		Mean	0.00	0.00	0.00	0.02
		SD	0.00	0.00	0.00	0.01			SD	0.02	0.01	0.01	0.03
	p	< 0.0001	a	b	b	c		p	< 0.0001	a	ab	b	c

ID - inverse difference; IDM - inverse difference moment; IDMN - inverse difference moment normalized; IDN - inverse difference normalized; IMC 1 - informational measure of correlation 1; IMC 2 - informational measure of correlation 2; MCC - maximal correlation coefficient. Lower case letters (a–c) indicate differences between classes for p < 0.05 independently for each feature.

Table S13. The values (mean ±SD) of features of *Gray Level Co-occurence Matrix (GLCM)* of output images, filtrated by *Bilateral* filter, compared between four classes (0-3) of the Equine Odontoclastic Tooth Resorption and Hypercementosis (EOTRH) syndrome. When features differed significantly (p<0.05).

Feature		EOTRH	0	1	2	3	Feature		EOTRH	0	1	2	3
**Autocorrelation**		Mean	7.35	10.69	11.40	14.19	**IDN**		Mean	0.93	0.92	0.92	0.92
		SD	2.71	3.61	4.25	4.61			SD	0.02	0.02	0.02	0.02
	p	0.0001	a	b	b	c		p	< 0.0001	a	b	b	b
**Cluster Prominence**		Mean	5.99	7.57	8.04	21.05	**IMC 1**		Mean	-0.22	-0.19	-0.19	-0.18
		SD	5.27	9.61	7.40	28.98			SD	0.06	0.07	0.07	0.06
	p	0.0001	a	a	a	b		p	< 0.0001	a	b	b	b
**Cluster Shade**		Mean	0.42	-0.31	-0.59	-1.31	**IMC 2**		Mean	0.64	0.60	0.60	0.63
		SD	0.76	1.00	0.89	2.26			SD	0.10	0.12	0.12	0.11
	p	< 0.0001	a	b	b	c		p	0.0027	a	b	b	ab
**Cluster Tendency**		Mean	1.35	1.42	1.54	2.40	**Inverse Variance**		Mean	0.33	0.36	0.38	0.40
		SD	0.63	0.74	0.67	1.63			SD	0.07	0.07	0.07	0.06
	p	< 0.0001	a	a	a	b		p	< 0.0001	a	b	bc	c
**Contrast**		Mean	0.38	0.52	0.55	0.70	**Joint Average**		Mean	2.62	3.19	3.28	3.66
		SD	0.12	0.19	0.21	0.35			SD	0.48	0.57	0.62	0.60
	p	< 0.0001	a	b	b	c		p	< 0.0001	a	b	b	c
**Correlation**		Mean	0.54	0.43	0.45	0.50	**Joint Energy**		Mean	0.28	0.26	0.23	0.19
		SD	0.10	0.15	0.16	0.13			SD	0.10	0.09	0.07	0.08
	p	< 0.0001	a	b	bc	c		p	< 0.0001	a	ab	b	c
**Difference Average**		Mean	0.35	0.43	0.45	0.53	**Joint Entropy**		Mean	2.40	2.56	2.66	3.02
		SD	0.09	0.11	0.12	0.17			SD	0.44	0.44	0.41	0.65
	p	< 0.0001	a	b	bc	c		p	< 0.0001	a	b	b	c
**Difference Entropy**		Mean	0.95	1.10	1.11	1.22	**MCC**		Mean	0.60	0.55	0.54	0.55
		SD	0.13	0.15	0.16	0.22			SD	0.10	0.13	0.13	0.11
	p	< 0.0001	a	b	b	c		p	0.0005	a	b	b	ab
**Difference Variance**		Mean	0.24	0.31	0.31	0.38	**Maximum Probability**		Mean	0.43	0.43	0.38	0.33
		SD	0.05	0.08	0.09	0.13			SD	0.13	0.12	0.10	0.13
	p	< 0.0001	a	b	b	c		p	< 0.0001	a	a	ab	b
**ID**		Mean	0.83	0.80	0.79	0.76	**Sum Average**		Mean	5.25	6.37	6.57	7.33
		SD	0.04	0.05	0.05	0.06			SD	0.95	1.14	1.24	1.20
	p	< 0.0001	a	b	bc	c		p	< 0.0001	a	b	b	c
**IDM**		Mean	0.83	0.79	0.78	0.75	**Sum Entropy**		Mean	2.00	2.04	2.13	2.37
		SD	0.04	0.05	0.05	0.07			SD	0.34	0.33	0.30	0.42
	p	< 0.0001	a	b	bc	c		p	< 0.0001	a	a	a	b
**IDMN**		Mean	0.98	0.98	0.98	0.98	**Sum Squares**		Mean	0.43	0.48	0.52	0.78
		SD	0.01	0.01	0.01	0.01			SD	0.18	0.21	0.20	0.48
	p	0.042	a	b	ab	ab		p	< 0.0001	a	a	b	c

ID - inverse difference; IDM - inverse difference moment; IDMN - inverse difference moment normalized; IDN - inverse difference normalized; IMC 1 - informational measure of correlation 1; IMC 2 - informational measure of correlation 2; MCC - maximal correlation coefficient. Lower case letters (a–c) indicate differences between classes for p < 0.05 independently for each feature.

Table S14. The values (mean ±SD) of features of *Gray Level Co-occurence Matrix (GLCM)* of output images, filtrated by *Binomial* filter, compared between four classes (0-3) of the Equine Odontoclastic Tooth Resorption and Hypercementosis (EOTRH) syndrome. When features differed significantly (p<0.05).

Feature	EOTRH	0	1	2	3	Feature	EOTRH	0	1	2	3
**Autocorrelation**	Mean	11.4	14.6	15.4	19.0	**IDN**	Mean	0.90	0.90	0.90	0.90
	SD	3.6	4.4	4.9	6.1		SD	0.02	0.01	0.02	0.02
p	< 0.0001	a	b	b	c	p	0.0591	a	a	a	a
**Cluster Prominence**	Mean	13.2	13.8	16.4	40.0	**IMC 1**	Mean	-0.10	-0.10	-0.10	-0.10
	SD	10.4	18.3	13.2	50.1		SD	0.03	0.03	0.04	0.04
p	< 0.0001	a	a	a	b	p	0.8716	a	a	a	a
**Cluster Shade**	Mean	0.50	-0.58	-1.05	-2.40	**IMC 2**	Mean	0.51	0.50	0.51	0.53
	SD	0.98	1.21	1.41	2.78		SD	0.09	0.09	0.10	0.10
p	< 0.0001	a	b	c	d	p	0.1289	a	a	a	a
**Cluster Tendency**	Mean	2.01	1.91	2.23	3.33	**Inverse Variance**	Mean	0.48	0.47	0.48	0.47
	SD	0.86	0.95	0.91	2.15		SD	0.04	0.04	0.04	0.04
p	< 0.0001	ab	a	b	c	p	0.008	ab	a	b	ab
**Contrast**	Mean	0.95	1.04	1.10	1.41	**Joint Average**	Mean	3.29	3.75	3.84	4.25
	SD	0.30	0.34	0.34	0.77		SD	0.52	0.56	0.62	0.68
p	0.0001	a	ab	b	b	p	< 0.0001	a	a	b	c
**Correlation**	Mean	0.34	0.27	0.32	0.38	**Joint Energy**	Mean	0.14	0.16	0.13	0.11
	SD	0.11	0.14	0.15	0.13		SD	0.05	0.05	0.04	0.05
p	< 0.0001	ac	b	a	c	p	< 0.0001	ab	a	bc	c
**Difference Average**	Mean	0.68	0.71	0.74	0.83	**Joint Entropy**	Mean	3.32	3.28	3.45	3.77
	SD	0.13	0.13	0.13	0.27		SD	0.44	0.42	0.39	0.71
p	0.0006	a	ab	bc	c	p	< 0.0001	ab	a	bc	c
**Difference Entropy**	Mean	1.37	1.43	1.45	1.55	**MCC**	Mean	0.44	0.42	0.42	0.44
	SD	0.15	0.14	0.15	0.27		SD	0.09	0.09	0.10	0.11
p	< 0.0001	a	b	b	b	p	0.0594	a	a	a	a
**Difference Variance**	Mean	0.45	0.50	0.51	0.63	**Maximum Probability**	Mean	0.25	0.28	0.24	0.22
	SD	0.10	0.12	0.13	0.27		SD	0.08	0.09	0.07	0.10
p	< 0.0001	a	b	b	b	p	< 0.0001	a	b	a	a
**ID**	Mean	0.70	0.70	0.68	0.66	**Sum Average**	Mean	6.59	7.50	7.68	8.51
	SD	0.04	0.04	0.04	0.07		SD	1.04	1.12	1.23	1.35
p	0.0014	a	a	ab	b	p	< 0.0001	a	b	b	c
**IDM**	Mean	0.69	0.68	0.67	0.64	**Sum Entropy**	Mean	2.41	2.36	2.49	2.68
	SD	0.05	0.05	0.05	0.09		SD	0.28	0.28	0.27	0.39
p	0.0013	a	ab	bc	c	p	< 0.0001	ab	a	bc	c
**IDMN**	Mean	0.97	0.97	0.97	0.97	**Sum Squares**	Mean	0.74	0.74	0.83	1.18
	SD	0.01	0.01	0.01	0.01		SD	0.28	0.30	0.28	0.70
p	0.2595	a	a	a	a	p	< 0.0001	a	a	b	b

ID - inverse difference; IDM - inverse difference moment; IDMN - inverse difference moment normalized; IDN - inverse difference normalized; IMC 1 - informational measure of correlation 1; IMC 2 - informational measure of correlation 2; MCC - maximal correlation coefficient. Lower case letters (a–d) indicate differences between classes for p < 0.05 independently for each feature.

Table S15. The values (mean ±SD) of features of *Gray Level Co-occurence Matrix (GLCM)* of output images, filtrated by *CurvatureFlow* filter, compared between four classes (0-3) of the Equine Odontoclastic Tooth Resorption and Hypercementosis (EOTRH) syndrome. When features differed significantly (p<0.05).

Feature		EOTRH	0	1	2	3	Feature		EOTRH	0	1	2	3
**Autocorrelation**		Mean	12.8	15.9	16.6	21.0	**IDN**		Mean	0.90	0.90	0.90	0.90
		SD	3.6	4.6	5.3	7.6			SD	0.02	0.01	0.02	0.02
	p	< 0.0001	a	b	b	c		p	0.5741	a	a	a	a
**Cluster Prominence**		Mean	15.5	15.8	18.6	45.8	**IMC 1**		Mean	-0.09	-0.09	-0.09	-0.09
		SD	11.8	20.6	14.8	55.9			SD	0.03	0.03	0.03	0.04
	p	< 0.0001	a	a	a	b		p	0.9815	a	a	a	a
**Cluster Shade**		Mean	0.55	-0.54	-1.08	-2.73	**IMC 2**		Mean	0.49	0.47	0.48	0.50
		SD	1.06	1.30	1.48	2.99			SD	0.09	0.09	0.10	0.10
	p	< 0.0001	a	b	c	d		p	0.1828	a	a	a	a
**Cluster Tendency**		Mean	2.17	2.04	2.36	3.53	**Inverse Variance**		Mean	0.49	0.48	0.49	0.47
		SD	0.91	1.00	0.94	2.29			SD	0.03	0.04	0.03	0.03
	p	< 0.0001	ab	a	b	c		p	0.0005	a	ab	a	b
**Contrast**		Mean	1.11	1.16	1.23	1.61	**Joint Average**		Mean	3.50	3.91	4.00	4.46
		SD	0.36	0.38	0.37	0.94			SD	0.49	0.57	0.62	0.80
	p	0.0021	a	ab	b	b		p	< 0.0001	a	b	b	c
**Correlation**		Mean	0.31	0.25	0.29	0.36	**Joint Energy**		Mean	0.13	0.14	0.12	0.11
		SD	0.11	0.14	0.15	0.12			SD	0.04	0.05	0.04	0.05
	p	< 0.0001	ac	b	a	c		p	< 0.0001	a	b	a	a
**Difference Average**		Mean	0.75	0.76	0.79	0.89	**Joint Entropy**		Mean	3.49	3.42	3.59	3.91
		SD	0.15	0.14	0.14	0.30			SD	0.45	0.42	0.39	0.74
	p	0.0084	a	ab	ab	b		p	< 0.0001	ab	a	bc	c
**Difference Entropy**		Mean	1.45	1.49	1.52	1.63	**MCC**		Mean	0.41	0.39	0.40	0.42
		SD	0.16	0.15	0.15	0.29			SD	0.09	0.09	0.10	0.10
	p	0.0006	a	ab	b	b		p	0.1047	a	a	a	a
**Difference Variance**		Mean	0.51	0.55	0.56	0.71	**Maximum Probability**		Mean	0.23	0.26	0.22	0.20
		SD	0.13	0.14	0.14	0.33			SD	0.08	0.09	0.07	0.09
	p	< 0.0001	a	b	b	b		p	< 0.0001	a	b	a	a
**ID**		Mean	0.68	0.68	0.67	0.65	**Sum Average**		Mean	7.01	7.83	8.00	8.92
		SD	0.04	0.04	0.04	0.07			SD	0.97	1.14	1.24	1.60
	p	0.0116	ab	a	ab	b		p	< 0.0001	a	b	b	c
**IDM**		Mean	0.66	0.66	0.65	0.62	**Sum Entropy**		Mean	2.48	2.42	2.54	2.74
		SD	0.05	0.05	0.05	0.09			SD	0.28	0.27	0.26	0.40
	p	0.0112	ab	a	ab	b		p	< 0.0001	ab	a	bc	c
**IDMN**		Mean	0.97	0.97	0.97	0.97	**Sum Squares**		Mean	0.82	0.80	0.90	1.29
		SD	0.01	0.01	0.01	0.01			SD	0.30	0.32	0.29	0.78
	p	0.8714	a	a	a	a		p	< 0.0001	ab	a	bc	c

ID - inverse difference; IDM - inverse difference moment; IDMN - inverse difference moment normalized; IDN - inverse difference normalized; IMC 1 - informational measure of correlation 1; IMC 2 - informational measure of correlation 2; MCC - maximal correlation coefficient. Lower case letters (a–d) indicate differences between classes for p < 0.05 independently for each feature.

Table S16. The values (mean ±SD) of features of *Gray Level Co-occurence Matrix (GLCM)* of output images, filtrated by *LaplacianSharpening* filter, compared between four classes (0-3) of the Equine Odontoclastic Tooth Resorption and Hypercementosis (EOTRH) syndrome. When features differed significantly (p<0.05).

Feature		EOTRH	0	1	2	3	Feature		EOTRH	0	1	2	3
**Autocorrelation**		Mean	31.3	36.6	38.5	47.1	**IDN**		Mean	0.88	0.89	0.89	0.88
		SD	8.9	11.9	11.8	13.8			SD	0.01	0.01	0.01	0.02
	p	< 0.0001	a	b	b	c		p	0.3321	a	a	a	a
**Cluster Prominence**		Mean	45.3	44.6	54.3	134.6	**IMC 1**		Mean	-0.03	-0.03	-0.03	-0.03
		SD	29.7	37.5	35.1	150.4			SD	0.01	0.01	0.01	0.01
	p	< 0.0001	a	a	a	b		p	0.6204	a	a	a	a
**Cluster Shade**		Mean	0.32	-1.38	-2.32	-6.71	**IMC 2**		Mean	0.33	0.32	0.32	0.35
		SD	1.37	2.09	2.81	6.46			SD	0.06	0.05	0.07	0.08
	p	< 0.0001	a	b	b	c		p	0.1247	a	a	a	a
**Cluster Tendency**		Mean	3.71	3.52	3.96	5.84	**Inverse Variance**		Mean	0.47	0.47	0.47	0.44
		SD	1.30	1.31	1.31	3.63			SD	0.02	0.02	0.02	0.05
	p	< 0.0001	ab	a	bc	c		p	0.002	a	a	ab	b
**Contrast**		Mean	2.69	2.70	2.87	3.97	**Joint Average**		Mean	5.52	5.95	6.11	6.75
		SD	0.83	0.81	0.83	2.49			SD	0.78	0.97	0.96	1.05
	p	0.0098	a	a	ab	b		p	< 0.0001	a	b	b	c
**Correlation**		Mean	0.15	0.12	0.15	0.19	**Joint Energy**		Mean	0.06	0.06	0.06	0.05
		SD	0.08	0.08	0.09	0.09			SD	0.02	0.02	0.02	0.02
	p	< 0.0001	ac	b	a	c		p	0.0006	ab	a	b	b
**Difference Average**		Mean	1.23	1.23	1.27	1.45	**Joint Entropy**		Mean	4.60	4.53	4.66	4.97
		SD	0.20	0.19	0.19	0.47			SD	0.43	0.40	0.41	0.75
	p	0.029	a	a	b	b		p	0.0005	ab	a	b	b
**Difference Entropy**		Mean	1.97	1.98	2.02	2.15	**MCC**		Mean	0.24	0.23	0.23	0.25
		SD	0.17	0.16	0.16	0.34			SD	0.05	0.05	0.06	0.07
	p	0.0062	a	a	ab	b		p	0.0542	a	a	a	a
**Difference Variance**		Mean	1.11	1.15	1.21	1.65	**Maximum Probability**		Mean	0.11	0.13	0.12	0.11
		SD	0.31	0.31	0.32	0.93			SD	0.04	0.04	0.04	0.05
	p	0.0009	a	a	ab	b		p	0.0008	a	b	a	a
**ID**		Mean	0.56	0.57	0.56	0.54	**Sum Average**		Mean	11.04	11.90	12.22	13.50
		SD	0.04	0.03	0.04	0.07			SD	1.57	1.94	1.92	2.09
	p	0.0673	a	a	a	a		p	< 0.0001	a	b	b	c
**IDM**		Mean	0.52	0.52	0.51	0.49	**Sum Entropy**		Mean	2.93	2.88	2.97	3.15
		SD	0.05	0.04	0.04	0.09			SD	0.24	0.23	0.23	0.39
	p	0.0623	a	a	a	a		p	< 0.0001	ab	a	bc	c
**IDMN**		Mean	0.97	0.97	0.97	0.97	**Sum Squares**		Mean	1.60	1.56	1.71	2.45
		SD	0.01	0.01	0.01	0.01			SD	0.52	0.51	0.50	1.51
	p	0.4064	a	a	a	a		p	< 0.0001	ab	a	bc	c

ID - inverse difference; IDM - inverse difference moment; IDMN - inverse difference moment normalized; IDN - inverse difference normalized; IMC 1 - informational measure of correlation 1; IMC 2 - informational measure of correlation 2; MCC - maximal correlation coefficient. Lower case letters (a–c) indicate differences between classes for p < 0.05 independently for each feature.

Table S17. The values (mean ±SD) of features of *Gray Level Co-occurence Matrix (GLCM)* of output images, filtrated by *DiscreteGaussian* filter, compared between four classes (0-3) of the Equine Odontoclastic Tooth Resorption and Hypercementosis (EOTRH) syndrome. When features differed significantly (p<0.05).

Feature	EOTRH	0	1	2	3	Feature	EOTRH	0	1	2	3
**Autocorrelation**	Mean	10.8	13.8	14.5	18.3	**IDN**	Mean	0.90	0.90	0.90	0.90
	SD	3.6	4.1	4.5	6.0		SD	0.02	0.02	0.02	0.02
p	< 0.0001	a	b	b	c	p	0.0245	ab	a	b	b
**Cluster Prominence**	Mean	11.9	12.8	15.2	36.5	**IMC 1**	Mean	-0.12	-0.11	-0.12	-0.11
	SD	9.6	16.8	12.3	45.7		SD	0.04	0.04	0.04	0.04
p	< 0.0001	a	a	a	b	p	0.5215	a	a	a	a
**Cluster Shade**	Mean	0.47	-0.59	-1.01	-2.19	**IMC 2**	Mean	0.53	0.51	0.53	0.55
	SD	0.92	1.17	1.35	2.73		SD	0.10	0.09	0.10	0.10
p	< 0.0001	a	b	bc	c	p	0.0394	ab	a	b	b
**Cluster Tendency**	Mean	1.92	1.84	2.16	3.20	**Inverse Variance**	Mean	0.47	0.46	0.48	0.47
	SD	0.83	0.92	0.88	2.05		SD	0.04	0.05	0.04	0.04
p	< 0.0001	ab	a	b	c	p	0.0063	a	b	a	ab
**Contrast**	Mean	0.85	0.96	1.02	1.28	**Joint Average**	Mean	3.20	3.64	3.73	4.16
	SD	0.26	0.31	0.32	0.66		SD	0.53	0.55	0.59	0.70
p	< 0.0001	a	b	bc	c	p	< 0.0001	a	b	b	c
**Correlation**	Mean	0.37	0.29	0.34	0.40	**Joint Energy**	Mean	0.15	0.17	0.14	0.12
	SD	0.11	0.14	0.16	0.13		SD	0.05	0.06	0.04	0.06
p	< 0.0001	ac	b	a	c	p	< 0.0001	a	a	ab	b
**Difference Average**	Mean	0.64	0.67	0.71	0.79	**Joint Entropy**	Mean	3.21	3.18	3.36	3.68
	SD	0.12	0.13	0.13	0.24		SD	0.43	0.42	0.39	0.68
p	< 0.0001	a	ab	bc	c	p	< 0.0001	a	a	a	b
**Difference Entropy**	Mean	1.31	1.39	1.40	1.51	**MCC**	Mean	0.46	0.43	0.44	0.46
	SD	0.14	0.14	0.15	0.25		SD	0.09	0.10	0.10	0.11
p	< 0.0001	a	b	b	b	p	0.0114	a	b	ab	ab
**Difference Variance**	Mean	0.41	0.47	0.48	0.58	**Maximum Probability**	Mean	0.26	0.30	0.25	0.23
	SD	0.09	0.11	0.12	0.23		SD	0.09	0.10	0.07	0.10
p	< 0.0001	a	b	b	b	p	< 0.0001	a	b	a	a
**ID**	Mean	0.71	0.71	0.69	0.67	**Sum Average**	Mean	6.39	7.28	7.46	8.32
	SD	0.04	0.04	0.04	0.07		SD	1.06	1.09	1.18	1.40
p	0.0001	a	ab	bc	c	p	< 0.0001	a	b	b	c
**IDM**	Mean	0.70	0.69	0.68	0.65	**Sum Entropy**	Mean	2.37	2.32	2.46	2.65
	SD	0.05	0.05	0.05	0.08		SD	0.28	0.29	0.27	0.39
p	< 0.0001	a	ab	bc	c	p	< 0.0001	a	a	ab	b
**IDMN**	Mean	0.97	0.97	0.97	0.97	**Sum Squares**	Mean	0.69	0.70	0.79	1.12
	SD	0.01	0.01	0.01	0.01		SD	0.26	0.28	0.26	0.65
p	0.2025	a	a	a	a	p	< 0.0001	a	a	b	b

ID - inverse difference; IDM - inverse difference moment; IDMN - inverse difference moment normalized; IDN - inverse difference normalized; IMC 1 - informational measure of correlation 1; IMC 2 - informational measure of correlation 2; MCC - maximal correlation coefficient. Lower case letters (a–d) indicate differences between classes for p < 0.05 independently for each feature.

Table S18. The values (mean ±SD) of features of *Gray Level Co-occurence Matrix (GLCM)* of output images, filtrated by *SmoothingRecursiveGaussian* filter, compared between four classes (0-3) of the Equine Odontoclastic Tooth Resorption and Hypercementosis (EOTRH) syndrome. When features differed significantly (p<0.05).

Feature		EOTRH	0	1	2	3	Feature		EOTRH	0	1	2	3
**Autocorrelation**		Mean	10.6	13.7	14.5	18.2	**IDN**		Mean	0.90	0.90	0.90	0.90
		SD	3.9	4.0	4.8	6.2			SD	0.02	0.02	0.02	0.02
	p	< 0.0001	a	b	b	c		p	0.012	ab	a	b	b
**Cluster Prominence**		Mean	11.9	12.8	15.2	36.3	**IMC 1**		Mean	-0.12	-0.11	-0.12	-0.11
		SD	9.5	16.6	12.3	45.4			SD	0.04	0.04	0.04	0.04
	p	< 0.0001	a	a	a	b		p	0.4625	a	a	a	a
**Cluster Shade**		Mean	0.47	-0.59	-1.02	-2.17	**IMC 2**		Mean	0.54	0.51	0.53	0.55
		SD	0.93	1.18	1.36	2.71			SD	0.10	0.09	0.10	0.10
	p	< 0.0001	a	b	b	c		p	0.0735	a	a	a	a
**Cluster Tendency**		Mean	1.91	1.84	2.15	3.19	**Inverse Variance**		Mean	0.47	0.46	0.47	0.47
		SD	0.82	0.92	0.88	2.04			SD	0.04	0.05	0.04	0.04
	p	< 0.0001	ab	a	b	c		p	0.0051	ab	a	b	ab
**Contrast**		Mean	0.84	0.96	1.02	1.27	**Joint Average**		Mean	3.17	3.63	3.71	4.15
		SD	0.25	0.31	0.32	0.65			SD	0.57	0.53	0.64	0.71
	p	< 0.0001	a	b	bc	c		p	< 0.0001	a	b	b	c
**Correlation**		Mean	0.37	0.29	0.34	0.40	**Joint Energy**		Mean	0.16	0.17	0.14	0.12
		SD	0.11	0.14	0.16	0.13			SD	0.05	0.06	0.04	0.06
	p	< 0.0001	ac	b	a	c		p	< 0.0001	ab	a	bc	c
**Difference Average**		Mean	0.63	0.67	0.70	0.79	**Joint Entropy**		Mean	3.20	3.18	3.35	3.67
		SD	0.12	0.13	0.13	0.24			SD	0.43	0.42	0.39	0.68
	p	< 0.0001	a	ab	bc	b		p	< 0.0001	ab	a	bc	c
**Difference Entropy**		Mean	1.31	1.39	1.40	1.50	**MCC**		Mean	0.47	0.43	0.44	0.46
		SD	0.14	0.14	0.15	0.25			SD	0.10	0.10	0.10	0.11
	p	< 0.0001	a	b	b	b		p	0.0104	a	b	ab	ab
**Difference Variance**		Mean	0.41	0.47	0.48	0.57	**Maximum Probability**		Mean	0.26	0.30	0.25	0.23
		SD	0.09	0.11	0.12	0.23			SD	0.09	0.10	0.07	0.10
	p	< 0.0001	a	b	b	b		p	< 0.0001	a	b	a	a
**ID**		Mean	0.72	0.71	0.70	0.67	**Sum Average**		Mean	6.34	7.26	7.42	8.29
		SD	0.04	0.04	0.04	0.07			SD	1.14	1.07	1.27	1.42
	p	< 0.0001	a	ab	bc	c		p	< 0.0001	a	b	b	c
**IDM**		Mean	0.70	0.69	0.68	0.65	**Sum Entropy**		Mean	2.37	2.32	2.45	2.65
		SD	0.05	0.05	0.05	0.08			SD	0.28	0.29	0.27	0.39
	p	< 0.0001	a	ab	bc	c		p	< 0.0001	ab	a	bc	c
**IDMN**		Mean	0.97	0.97	0.97	0.97	**Sum Squares**		Mean	0.69	0.70	0.79	1.12
		SD	0.01	0.01	0.01	0.01			SD	0.26	0.28	0.26	0.65
	p	0.1191	a	a	a	a		p	< 0.0001	a	a	b	b

ID - inverse difference; IDM - inverse difference moment; IDMN - inverse difference moment normalized; IDN - inverse difference normalized; IMC 1 - informational measure of correlation 1; IMC 2 - informational measure of correlation 2; MCC - maximal correlation coefficient. Lower case letters (a–c) indicate differences between classes for p < 0.05 independently for each feature.

Table S19. The values (mean ±SD) of features of *Neighbouring Gray Tone Difference Matrix (NGTDM)* of output images, filtrated by *Mean* filter, compared between four classes (0-3) of the Equine Odontoclastic Tooth Resorption and Hypercementosis (EOTRH) syndrome. When features differed significantly (p<0.05).

**Feature**		**EOTRH**	**0**	**1**	**2**	**3**
**Busyness**		Mean	18.4	13.1	15.1	11.9
		SD	12.9	8.3	8.9	7.0
	p	< 0.0001	a	b	b	b
**Coarseness**		Mean	0.00	0.00	0.00	0.00
		SD	0.00	0.00	0.00	0.00
	p	0.0003	a	b	a	a
**Complexity**		Mean	6.75	7.64	7.86	10.38
		SD	2.93	3.10	2.87	6.23
	p	0.0002	a	b	b	b
**Contrast**		Mean	0.03	0.03	0.04	0.05
		SD	0.01	0.01	0.02	0.02
	p	0.0006	a	a	ab	b
**Strength**		Mean	0.04	0.05	0.04	0.06
		SD	0.02	0.02	0.02	0.04
	p	0.0001	a	b	ab	b

Lower case letters (a–c) indicate differences between classes for p < 0.05 independently for each feature.

Table S20. The values (mean ±SD) of features of *Neighbouring Gray Tone Difference Matrix (NGTDM)* of output images, filtrated by *Median* filter, compared between four classes (0-3) of the Equine Odontoclastic Tooth Resorption and Hypercementosis (EOTRH) syndrome. When features differed significantly (p<0.05).

**Feature**		**EOTRH**	**0**	**1**	**2**	**3**
**Busyness**		Mean	17.1	12.1	14.7	11.4
		SD	8.7	6.7	8.8	6.9
	p	< 0.0001	a	bc	b	c
**Coarseness**		Mean	0.004	0.004	0.004	0.004
		SD	0.001	0.002	0.002	0.002
	p	0.0002	a	b	a	ab
**Complexity**		Mean	7.40	8.29	8.53	11.41
		SD	3.17	3.31	3.14	6.88
	p	0.0004	a	b	b	b
**Contrast**		Mean	0.04	0.03	0.04	0.05
		SD	0.02	0.01	0.01	0.02
	p	0.0029	ab	a	ab	b
**Strength**		Mean	0.04	0.05	0.05	0.06
		SD	0.02	0.02	0.02	0.04
	p	< 0.0001	a	bc	ab	c

Lower case letters (a–c) indicate differences between classes for p < 0.05 independently for each feature.

Table S21. The values (mean ±SD) of features of *Neighbouring Gray Tone Difference Matrix (NGTDM)* of output images, filtrated by *Normalize* filter, compared between four classes (0-3) of the Equine Odontoclastic Tooth Resorption and Hypercementosis (EOTRH) syndrome. When features differed significantly (p<0.05).

Feature		EOTRH	0	1	2	3
**Busyness**		Mean	43.0	1.5	1.4	6.2
		SD	439.6	11.6	3.7	10.4
	p	< 0.0001	a	b	bc	c
**Coarseness**		Mean	904762	753846	621622	409836
		SD	294951	431878	487182	495885
	p	< 0.0001	a	b	bc	c
**Complexity**		Mean	0.00	0.00	0.00	0.03
		SD	0.01	0.02	0.01	0.05
	p	< 0.0001	a	ab	b	c
**Contrast**		Mean	0.0002	0.0003	0.0001	0.0015
		SD	0.0023	0.0036	0.0002	0.0037
	p	< 0.0001	a	b	bc	c
**Strength**		Mean	0.05	0.10	0.17	0.14
		SD	0.20	0.26	0.36	0.31
	p	< 0.0001	a	b	bc	c

Lower case letters (a–c) indicate differences between classes for p < 0.05 independently for each feature.

Table S22. The values (mean ±SD) of features of *Neighbouring Gray Tone Difference Matrix (NGTDM)* of output images, filtrated by *Bilateral* filter, compared between four classes (0-3) of the Equine Odontoclastic Tooth Resorption and Hypercementosis (EOTRH) syndrome. When features differed significantly (p<0.05).

Feature		EOTRH	0	1	2	3
**Busyness**		Mean	24.4	16.7	20.2	14.3
		SD	14.2	10.7	14.7	8.2
	p	< 0.0001	a	bc	b	c
**Coarseness**		Mean	0.006	0.006	0.005	0.005
		SD	0.002	0.002	0.002	0.002
	p	< 0.0001	ac	a	bc	c
**Complexity**		Mean	2.05	3.10	3.43	4.65
		SD	1.11	1.41	1.77	2.44
	p	< 0.0001	a	b	b	c
**Contrast**		Mean	0.024	0.027	0.028	0.034
		SD	0.011	0.011	0.012	0.017
	p	0.0003	a	ab	ab	b
**Strength**		Mean	0.029	0.039	0.037	0.049
		SD	0.014	0.020	0.025	0.031
	p	< 0.0001	a	bc	ab	c

Lower case letters (a–c) indicate differences between classes for p < 0.05 independently for each feature.

Table S23. The values (mean ±SD) of features of *Neighbouring Gray Tone Difference Matrix (NGTDM)* of output images, filtrated by *Binomial* filter, compared between four classes (0-3) of the Equine Odontoclastic Tooth Resorption and Hypercementosis (EOTRH) syndrome. When features differed significantly (p<0.05).

**Feature**		**EOTRH**	**0**	**1**	**2**	**3**
**Busyness**		Mean	17.4	12.4	14.8	11.6
		SD	7.5	7.7	8.5	6.9
	p	< 0.0001	a	bc	c	bc
**Coarseness**		Mean	0.004	0.004	0.004	0.004
		SD	0.001	0.002	0.002	0.002
	p	0.0003	a	b	a	a
**Complexity**		Mean	6.97	8.11	8.25	11.11
		SD	2.95	3.16	3.09	6.99
	p	< 0.0001	a	b	b	b
**Contrast**		Mean	0.036	0.034	0.038	0.046
		SD	0.013	0.011	0.015	0.021
	p	0.0003	a	a	ab	b
**Strength**		Mean	0.038	0.050	0.044	0.059
		SD	0.016	0.022	0.024	0.036
	p	< 0.0001	a	b	a	b

Lower case letters (a–c) indicate differences between classes for p < 0.05 independently for each feature.

Table S24. The values (mean ±SD) of features of *Neighbouring Gray Tone Difference Matrix (NGTDM)* of output images. filtrated by *CurvatureFlow* filter. compared between four classes (0-3) of the Equine Odontoclastic Tooth Resorption and Hypercementosis (EOTRH) syndrome. When features differed significantly (p<0.05).

**Feature**		**EOTRH**	**0**	**1**	**2**	**3**
**Busyness**		Mean	14.7	11.0	12.7	10.2
		SD	6.7	6.0	6.5	7.2
	p	< 0.0001	a	b	a	b
**Coarseness**		Mean	0.004	0.004	0.004	0.004
		SD	0.001	0.002	0.002	0.002
	p	0.0002	a	b	a	a
**Complexity**		Mean	9.17	9.84	10.37	14.59
		SD	3.78	3.79	3.98	9.44
	p	0.0005	a	ab	bc	c
**Contrast**		Mean	0.035	0.034	0.037	0.044
		SD	0.013	0.012	0.014	0.022
	p	0.0125	ab	a	ab	b
**Strength**		Mean	0.045	0.056	0.051	0.069
		SD	0.019	0.025	0.028	0.043
	p	< 0.0001	a	bc	ab	c

Lower case letters (a–c) indicate differences between classes for p < 0.05 independently for each feature.

Table S25. The values (mean ±SD) of features of *Neighbouring Gray Tone Difference Matrix (NGTDM)* of output images. filtrated by *LaplacianSharpening* filter. compared between four classes (0-3) of the Equine Odontoclastic Tooth Resorption and Hypercementosis (EOTRH) syndrome. When features differed significantly (p<0.05).

Feature		EOTRH	0	1	2	3
**Busyness**		Mean	6.58	5.28	5.76	4.84
		SD	3.47	3.28	3.00	3.34
	p	< 0.0001	a	bc	ab	c
**Coarseness**		Mean	0.004	0.004	0.004	0.004
		SD	0.001	0.002	0.002	0.002
	p	< 0.0001	a	b	a	a
**Complexity**		Mean	30.0	31.8	35.1	46.1
		SD	10.7	11.5	11.8	20.3
	p	< 0.0001	a	ab	b	c
**Contrast**		Mean	0.051	0.048	0.049	0.066
		SD	0.017	0.017	0.015	0.047
	p	0.3212	a	a	a	a
**Strength**		Mean	0.089	0.116	0.106	0.138
		SD	0.035	0.057	0.057	0.086
	p	< 0.0001	a	bc	ab	c

Lower case letters (a–c) indicate differences between classes for p < 0.05 independently for each feature.

Table S26. The values (mean ±SD) of features of *Neighbouring Gray Tone Difference Matrix (NGTDM)* of output images. filtrated by *DiscreteGaussian* filter. compared between four classes (0-3) of the Equine Odontoclastic Tooth Resorption and Hypercementosis (EOTRH) syndrome. When features differed significantly (p<0.05).

Feature		EOTRH	0	1	2	3
**Busyness**		Mean	18.1	13.5	16.3	12.4
		SD	7.5	8.3	9.8	7.9
	p	< 0.0001	a	b	b	c
**Coarseness**		Mean	0.004	0.005	0.004	0.004
		SD	0.001	0.002	0.002	0.002
	p	0.0003	a	b	a	a
**Complexity**		Mean	6.06	7.11	7.16	9.73
		SD	2.27	2.86	2.60	5.72
	p	< 0.0001	a	b	b	b
**Contrast**		Mean	0.034	0.034	0.039	0.045
		SD	0.014	0.013	0.015	0.021
	p	< 0.0001	a	ab	bc	c
**Strength**		Mean	0.036	0.047	0.041	0.057
		SD	0.015	0.022	0.021	0.037
	p	< 0.0001	a	bc	ab	c

Lower case letters (a–c) indicate differences between classes for p < 0.05 independently for each feature.

Table S27. The values (mean ±SD) of features of *Neighbouring Gray Tone Difference Matrix (NGTDM)* of output images. filtrated by *SmoothingRecursiveGaussian* filter. compared between four classes (0-3) of the Equine Odontoclastic Tooth Resorption and Hypercementosis (EOTRH) syndrome. When features differed significantly (p<0.05).

**Feature**		**EOTRH**	**0**	**1**	**2**	**3**
**Busyness**		Mean	18.9	13.5	16.9	12.7
		SD	9.3	8.3	10.6	8.0
	p	< 0.0001	a	b	a	b
**Coarseness**		Mean	0.004	0.005	0.004	0.004
		SD	0.001	0.002	0.002	0.002
	p	0.0003	a	b	a	a
**Complexity**		Mean	5.97	7.04	7.06	9.61
		SD	2.44	2.55	2.70	5.75
	p	< 0.0001	a	b	b	b
**Contrast**		Mean	0.034	0.035	0.039	0.045
		SD	0.015	0.013	0.015	0.020
	p	< 0.0001	a	a	b	b
**Strength**		Mean	0.036	0.047	0.057	0.057
		SD	0.015	0.022	0.038	0.038
	p	< 0.0001	a	b	b	b

Lower case letters (a–c) indicate differences between classes for p < 0.05 independently for each feature.

Table S28. The values (mean ±SD) of features of *Gray Level Dependence Matrix (GLDM)* of output images, filtrated by *Mean* filter, compared between four classes (0-3) of the Equine Odontoclastic Tooth Resorption and Hypercementosis (EOTRH) syndrome. When features differed significantly (p<0.05).

Feature		EOTRH	0	1	2	3
**DE**		Mean	6.25	6.08	6.23	6.34
		SD	0.28	0.35	0.36	0.26
	p	< 0.0001	a	b	a	a
**DN**		Mean	154.9	69.9	84.4	80.6
		SD	826.0	38.3	44.9	27.8
	p	< 0.0001	a	b	a	a
**DNN**		Mean	0.04	0.04	0.04	0.04
		SD	0.01	0.01	0.01	0.01
	p	0.9466	a	ab	a	a
**DV**		Mean	64.4	73.4	66.4	65.9
		SD	25.9	25.1	19.8	32.3
	p	0.0031	a	b	ab	ab
**GLN**		Mean	820.4	635.0	715.6	607.9
		SD	1560.0	368.7	432.3	307.0
	p	0.0268	a	b	ab	ab
**GLV**		Mean	0.82	0.79	0.87	1.19
		SD	0.73	0.29	0.28	0.62
	p	< 0.0001	a	ab	b	c
**HGLE**		Mean	12.0	14.4	15.4	19.0
		SD	4.0	4.3	4.6	6.4
	p	< 0.0001	a	b	b	c
**LDE**		Mean	302.2	292.5	283.8	275.8
		SD	107.3	88.8	83.3	128.4
	p	0.4497	a	ab	a	a
**LDHGLE**		Mean	3436	4433	4712	5466
		SD	1607	1902	2080	2479
	p	< 0.0001	a	b	bc	c
**LDLGLE**		Mean	40.0	24.7	22.6	18.4
		SD	46.2	14.5	11.3	11.0
	p	< 0.0001	a	b	bc	c
**LGLE**		Mean	0.14	0.11	0.10	0.09
		SD	0.06	0.04	0.04	0.03
	p	< 0.0001	a	b	bc	c
**SDE**		Mean	0.03	0.04	0.04	0.04
		SD	0.01	0.01	0.01	0.02
	p	< 0.0001	a	b	ab	b
**SDHGLE**		Mean	0.42	0.50	0.47	0.71
		SD	0.20	0.25	0.24	0.50
	p	< 0.0001	a	bc	ab	c
**SDLGLE**		Mean	0.01	0.01	0.01	0.01
		SD	0.00	0.00	0.00	0.00
	p	0.1662	a	ab	a	a

DE - dependence entropy; DN - dependence non-uniformity; DNN - dependence non-uniformity normalized; DV - dependence variance; GLN - gray level non-uniformity; GLV - gray level variance; HGLE - high gray level emphasis; LDE - large dependence emphasis; LDHGLE - large dependence high gray level emphasis; LDLGLE - large dependence low gray level emphasis; LGLE - low gray level emphasis; SDE - small dependence emphasis; SDHGLE - small dependence high gray level emphasis; SDLGLE - small dependence low gray level emphasis. Lower case letters (a–c) indicate differences between classes for p < 0.05 independently for each feature.

Table S29. The values (mean ±SD) of features of *Gray Level Dependence Matrix (GLDM)* of output images, filtrated by *Median* filter, compared between four classes (0-3) of the Equine Odontoclastic Tooth Resorption and Hypercementosis (EOTRH) syndrome. When features differed significantly (p<0.05).

Feature		EOTRH	0	1	2	3
**DE**		Mean	6.26	6.11	6.25	6.36
		SD	0.27	0.34	0.35	0.24
	p	< 0.0001	a	b	a	a
**DN**		Mean	75.3	69.9	84.4	81.1
		SD	27.5	38.3	45.2	28.6
	p	< 0.0001	a	b	a	a
**DNN**		Mean	0.04	0.04	0.04	0.04
		SD	0.01	0.01	0.01	0.01
	p	0.9214	a	a	a	a
**DV**		Mean	62.3	70.8	64.8	65.1
		SD	23.9	24.8	19.6	31.3
	p	0.0135	a	b	ab	ab
**GLN**		Mean	657.1	628.5	705.1	600.7
		SD	311.3	363.3	426.3	304.8
	p	0.0517	a	a	a	a
**GLV**		Mean	0.80	0.82	0.89	1.23
		SD	0.28	0.31	0.29	0.66
	p	< 0.0001	a	a	a	b
**HGLE**		Mean	12.3	14.9	15.6	19.4
		SD	3.7	4.3	4.7	7.1
	p	< 0.0001	a	b	b	c
**LDE**		Mean	284.2	282.7	274.9	270.3
		SD	97.0	86.4	82.3	126.3
	p	0.6995	a	a	a	a
**LDHGLE**		Mean	3282	4404	4620	5457
		SD	1454	1888	2053	2666
	p	< 0.0001	a	b	b	b
**LDLGLE**		Mean	33.7	23.0	21.9	18.1
		SD	18.2	12.9	11.8	11.1
	p	< 0.0001	a	b	b	b
**LGLE**		Mean	0.13	0.11	0.10	0.09
		SD	0.06	0.04	0.04	0.03
	p	< 0.0001	a	b	b	b
**SDE**		Mean	0.04	0.04	0.04	0.05
		SD	0.01	0.01	0.01	0.02
	p	0.0018	a	b	ab	b
**SDHGLE**		Mean	0.52	0.58	0.57	0.86
		SD	0.28	0.28	0.27	0.62
	p	0.0103	a	ab	ab	b
**SDLGLE**		Mean	0.01	0.01	0.01	0.01
		SD	0.00	0.00	0.00	0.01
	p	0.7173	a	a	a	a

DE - dependence entropy; DN - dependence non-uniformity; DNN - dependence non-uniformity normalized; DV - dependence variance; GLN - gray level non-uniformity; GLV - gray level variance; HGLE - high gray level emphasis; LDE - large dependence emphasis; LDHGLE - large dependence high gray level emphasis; LDLGLE - large dependence low gray level emphasis; LGLE - low gray level emphasis; SDE - small dependence emphasis; SDHGLE - small dependence high gray level emphasis; SDLGLE - small dependence low gray level emphasis. Lower case letters (a–c) indicate differences between classes for p < 0.05 independently for each feature.

Table S30. The values (mean ±SD) of features of *Gray Level Dependence Matrix (GLDM)* of output images, filtrated by *Normalize* filter, compared between four classes (0-3) of the Equine Odontoclastic Tooth Resorption and Hypercementosis (EOTRH) syndrome. When features differed significantly (p<0.05).

Feature		EOTRH	0	1	2	3
**DE**		Mean	3.93	3.99	3.77	3.84
		SD	0.45	0.47	0.45	0.63
	p	< 0.0001	a	a	b	ab
**DN**		Mean	688.9	324.4	475.1	401.2
		SD	3565.0	483.9	585.6	377.2
	p	< 0.0001	a	b	a	a
**DNN**		Mean	0.14	0.13	0.16	0.17
		SD	0.08	0.08	0.08	0.09
	p	< 0.0001	ab	a	b	b
**DV**		Mean	65.5	66.4	66.4	72.1
		SD	6.5	7.4	7.1	12.7
	p	0.0665	a	a	a	a
**GLN**		Mean	2432	1863	2254	1984
		SD	4710	1220	1435	847
	p	< 0.0001	a	b	a	a
**GLV**		Mean	0.003	0.002	0.002	0.014
		SD	0.024	0.015	0.006	0.025
	p	< 0.0001	a	b	b	c
**HGLE**		Mean	1.27	1.73	2.13	2.72
		SD	0.85	1.28	1.45	1.45
	p	< 0.0001	a	b	bc	c
**LDE**		Mean	1083	1024	1108	1122
		SD	128	127	122	137
	p	< 0.0001	a	b	a	a
**LDHGLE**		Mean	1387	1821	2411	3059
		SD	981	1463	1740	1664
	p	< 0.0001	a	a	b	c
**LDLGLE**		Mean	1007	825	782	637
		SD	255	333	399	444
	p	< 0.0001	a	b	b	b
**LGLE**		Mean	0.93	0.82	0.72	0.57
		SD	0.21	0.32	0.36	0.36
	p	< 0.0001	a	b	bc	c
**SDE**		Mean	0.001	0.002	0.002	0.004
		SD	0.000	0.001	0.002	0.004
	p	< 0.0001	a	b	ab	b
**SDHGLE**		Mean	0.002	0.003	0.004	0.006
		SD	0.002	0.003	0.004	0.005
	p	< 0.0001	a	b	b	b
**SDLGLE**		Mean	0.001	0.002	0.002	0.003
		SD	0.000	0.001	0.002	0.003
	p	0.001	a	b	a	ab

DE - dependence entropy; DN - dependence non-uniformity; DNN - dependence non-uniformity normalized; DV - dependence variance; GLN - gray level non-uniformity; GLV - gray level variance; HGLE - high gray level emphasis; LDE - large dependence emphasis; LDHGLE - large dependence high gray level emphasis; LDLGLE - large dependence low gray level emphasis; LGLE - low gray level emphasis; SDE - small dependence emphasis; SDHGLE - small dependence high gray level emphasis; SDLGLE - small dependence low gray level emphasis. Lower case letters (a–c) indicate differences between classes for p < 0.05 independently for each feature.

Table S31. The values (mean ±SD) of features of *Gray Level Dependence Matrix (GLDM)* of output images, filtrated by *Bilateral* filter, compared between four classes (0-3) of the Equine Odontoclastic Tooth Resorption and Hypercementosis (EOTRH) syndrome. When features differed significantly (p<0.05).

Feature		EOTRH	0	1	2	3
**DE**		Mean	6.08	5.95	6.09	6.20
		SD	0.32	0.41	0.39	0.35
	p	< 0.0001	ac	b	ab	c
**DN**		Mean	71.7	68.0	77.1	74.3
		SD	37.0	48.7	50.4	35.7
	p	< 0.0001	a	b	a	a
**DNN**		Mean	0.04	0.04	0.03	0.04
		SD	0.01	0.01	0.01	0.01
	p	0.0604	a	a	a	a
**DV**		Mean	88.3	96.9	94.4	92.7
		SD	20.5	20.8	21.7	32.1
	p	0.0175	a	b	ab	ab
**GLN**		Mean	864.4	765.3	885.9	742.5
		SD	395.0	430.8	507.1	372.8
	p	0.0017	a	b	a	ab
**GLV**		Mean	0.46	0.54	0.57	0.82
		SD	0.18	0.22	0.21	0.45
	p	< 0.0001	a	b	b	c
**HGLE**		Mean	7.57	10.63	11.32	14.19
		SD	2.74	3.49	4.15	4.55
	p	< 0.0001	a	b	b	c
**LDE**		Mean	563.9	483.4	495.1	455.4
		SD	132.3	124.3	138.6	161.0
	p	< 0.0001	a	b	b	b
**LDHGLE**		Mean	4114	5502	5936	7002
		SD	1751	2287	2433	3078
	p	< 0.0001	a	b	bc	c
**LDLGLE**		Mean	108.4	57.7	58.1	40.5
		SD	53.6	38.3	39.5	23.8
	p	< 0.0001	a	b	b	c
**LGLE**		Mean	0.21	0.15	0.14	0.12
		SD	0.09	0.07	0.07	0.05
	p	< 0.0001	a	b	bc	c
**SDE**		Mean	0.01	0.02	0.02	0.02
		SD	0.01	0.01	0.01	0.01
	p	< 0.0001	a	b	b	b
**SDHGLE**		Mean	0.10	0.14	0.16	0.23
		SD	0.08	0.10	0.12	0.15
	p	< 0.0001	a	b	bc	c
**SDLGLE**		Mean	0.01	0.01	0.01	0.01
		SD	0.00	0.00	0.00	0.00
	p	0.0041	a	b	ab	ab

DE - dependence entropy; DN - dependence non-uniformity; DNN - dependence non-uniformity normalized; DV - dependence variance; GLN - gray level non-uniformity; GLV - gray level variance; HGLE - high gray level emphasis; LDE - large dependence emphasis; LDHGLE - large dependence high gray level emphasis; LDLGLE - large dependence low gray level emphasis; LGLE - low gray level emphasis; SDE - small dependence emphasis; SDHGLE - small dependence high gray level emphasis; SDLGLE - small dependence low gray level emphasis. Lower case letters (a–c) indicate differences between classes for p < 0.05 independently for each feature.

Table S32. The values (mean ±SD) of features of *Gray Level Dependence Matrix (GLDM)* of output images, filtrated by *Binomial* filter, compared between four classes (0-3) of the Equine Odontoclastic Tooth Resorption and Hypercementosis (EOTRH) syndrome. When features differed significantly (p<0.05).

Feature		EOTRH	0	1	2	3
**DE**		Mean	6.25	6.09	6.24	6.34
		SD	0.27	0.35	0.35	0.25
	p	< 0.0001	a	b	a	a
**DN**		Mean	75.2	70.6	85.0	81.6
		SD	27.6	38.4	45.4	28.6
	p	< 0.0001	a	b	a	a
**DNN**		Mean	0.04	0.04	0.04	0.04
		SD	0.01	0.01	0.01	0.01
	p	0.9692	a	a	a	a
**DV**		Mean	61.8	70.8	64.5	64.5
		SD	23.4	24.6	19.7	31.9
	p	0.0059	a	b	ab	ab
**GLN**		Mean	661.3	628.0	706.8	602.3
		SD	312.4	364.8	427.9	306.6
	p	0.0679	a	a	a	a
**GLV**		Mean	0.78	0.81	0.89	1.22
		SD	0.26	0.30	0.28	0.65
	p	< 0.0001	a	ab	bc	c
**HGLE**		Mean	12.1	14.8	15.5	19.4
		SD	3.7	4.3	4.8	6.5
	p	< 0.0001	a	b	b	c
**LDE**		Mean	286.1	282.0	274.6	268.6
		SD	95.5	86.1	81.6	126.9
	p	0.5828	a	a	a	a
**LDHGLE**		Mean	3263	4377	4590	5375
		SD	1430	1855	1994	2391
	p	< 0.0001	a	b	bc	c
**LDLGLE**		Mean	34.1	22.7	21.8	17.6
		SD	18.0	11.9	10.9	10.6
	p	< 0.0001	a	b	b	b
**LGLE**		Mean	0.14	0.11	0.10	0.09
		SD	0.06	0.04	0.04	0.03
	p	< 0.0001	a	b	bc	c
**SDE**		Mean	0.03	0.04	0.04	0.04
		SD	0.01	0.01	0.01	0.02
	p	< 0.0001	a	ab	b	ab
**SDHGLE**		Mean	0.44	0.53	0.50	0.75
		SD	0.20	0.25	0.25	0.53
	p	0.0015	a	b	ab	b
**SDLGLE**		Mean	0.009	0.009	0.009	0.009
		SD	0.004	0.004	0.004	0.005
	p	0.7197	a	a	a	a

DE - dependence entropy; DN - dependence non-uniformity; DNN - dependence non-uniformity normalized; DV - dependence variance; GLN - gray level non-uniformity; GLV - gray level variance; HGLE - high gray level emphasis; LDE - large dependence emphasis; LDHGLE - large dependence high gray level emphasis; LDLGLE - large dependence low gray level emphasis; LGLE - low gray level emphasis; SDE - small dependence emphasis; SDHGLE - small dependence high gray level emphasis; SDLGLE - small dependence low gray level emphasis. Lower case letters (a–c) indicate differences between classes for p < 0.05 independently for each feature.

Table S33. The values (mean ±SD) of features of *Gray Level Dependence Matrix (GLDM)* of output images, filtrated by *CurvatureFlow* filter, compared between four classes (0-3) of the Equine Odontoclastic Tooth Resorption and Hypercementosis (EOTRH) syndrome. When features differed significantly (p<0.05).

Feature		EOTRH	0	1	2	3
**DE**		Mean	6.27	6.12	6.26	6.37
		SD	0.26	0.33	0.33	0.23
	p	< 0.0001	a	b	b	c
**DN**		Mean	78.1	72.4	87.4	84.0
		SD	28.1	39.5	47.2	30.4
	p	< 0.0001	a	b	a	a
**DNN**		Mean	0.04	0.04	0.04	0.04
		SD	0.01	0.01	0.01	0.01
	p	0.9037	a	a	a	a
**DV**		Mean	57.1	64.0	58.8	60.2
		SD	22.3	22.8	18.5	29.7
	p	0.0334	a	b	ab	ab
**GLN**		Mean	632.5	605.8	682.3	583.2
		SD	306.8	354.3	417.0	299.1
	p	0.0644	a	a	a	a
**GLV**		Mean	0.86	0.87	0.96	1.32
		SD	0.29	0.32	0.30	0.72
	p	< 0.0001	a	a	ab	b
**HGLE**		Mean	13.6	16.1	16.8	21.4
		SD	3.7	4.5	5.2	8.0
	p	< 0.0001	a	b	b	c
**LDE**		Mean	255.1	254.0	248.5	248.2
		SD	89.8	77.7	75.8	119.2
	p	0.8154	a	a	a	a
**LDHGLE**		Mean	3241	4263	4516	5393
		SD	1373	1805	2109	2534
	p	< 0.0001	a	b	bc	c
**LDLGLE**		Mean	26.0	18.8	17.6	15.4
		SD	13.2	9.4	7.9	10.5
	p	< 0.0001	a	b	b	b
**LGLE**		Mean	0.12	0.10	0.09	0.08
		SD	0.04	0.03	0.03	0.03
	p	< 0.0001	a	b	bc	c
**SDE**		Mean	0.04	0.04	0.04	0.05
		SD	0.01	0.01	0.01	0.02
	p	0.0063	a	b	ab	b
**SDHGLE**		Mean	0.60	0.64	0.62	0.97
		SD	0.28	0.28	0.28	0.72
	p	0.0379	a	ab	ab	b
**SDLGLE**		Mean	0.009	0.009	0.009	0.009
		SD	0.005	0.004	0.004	0.005
	p	0.5825	a	a	a	a

DE - dependence entropy; DN - dependence non-uniformity; DNN - dependence non-uniformity normalized; DV - dependence variance; GLN - gray level non-uniformity; GLV - gray level variance; HGLE - high gray level emphasis; LDE - large dependence emphasis; LDHGLE - large dependence high gray level emphasis; LDLGLE - large dependence low gray level emphasis; LGLE - low gray level emphasis; SDE - small dependence emphasis; SDHGLE - small dependence high gray level emphasis; SDLGLE - small dependence low gray level emphasis. Lower case letters (a–c) indicate differences between classes for p < 0.05 independently for each feature.

Table S34. The values (mean ±SD) of features of *Gray Level Dependence Matrix (GLDM)* of output images, filtrated by *LaplacianSharpening* filter, compared between four classes (0-3) of the Equine Odontoclastic Tooth Resorption and Hypercementosis (EOTRH) syndrome. When features differed significantly (p<0.05).

Feature		EOTRH	0	1	2	3
**DE**		Mean	6.21	6.10	6.19	6.31
		SD	0.19	0.23	0.23	0.18
	p	< 0.0001	ac	b	c	d
**DN**		Mean	111.7	105.6	126.7	122.0
		SD	43.0	61.8	73.7	47.1
	p	< 0.0001	a	b	a	a
**DNN**		Mean	0.06	0.06	0.06	0.06
		SD	0.01	0.01	0.01	0.02
	p	0.9146	a	a	a	a
**DV**		Mean	27.1	27.4	27.3	27.1
		SD	9.2	8.0	9.0	12.6
	p	0.9308	a	a	a	a
**GLN**		Mean	450.3	428.1	495.1	425.5
		SD	226.6	256.6	301.1	218.3
	p	0.0073	ab	a	b	ab
**GLV**		Mean	1.70	1.68	1.83	2.52
		SD	0.52	0.50	0.51	1.41
	p	< 0.0001	a	a	ab	b
**HGLE**		Mean	33.2	37.5	39.4	48.6
		SD	9.2	11.8	11.8	14.2
	p	< 0.0001	a	b	b	c
**LDE**		Mean	113.5	110.5	112.8	112.1
		SD	36.2	28.7	31.9	50.9
	p	0.9718	a	a	a	a
**LDHGLE**		Mean	3547	4193	4580	5366
		SD	1522	1679	1763	2277
	p	< 0.0001	a	b	bc	c
**LDLGLE**		Mean	4.40	3.56	3.39	2.96
		SD	2.07	1.83	1.73	2.31
	p	< 0.0001	a	b	bc	c
**LGLE**		Mean	0.05	0.04	0.04	0.03
		SD	0.02	0.02	0.02	0.01
	p	< 0.0001	a	b	bc	c
**SDE**		Mean	0.07	0.08	0.08	0.08
		SD	0.02	0.01	0.02	0.02
	p	0.0261	a	b	ab	ab
**SDHGLE**		Mean	2.51	2.60	2.67	3.48
		SD	0.98	0.98	1.08	1.75
	p	0.0026	a	a	a	b
**SDLGLE**		Mean	0.007	0.007	0.007	0.006
		SD	0.003	0.003	0.004	0.003
	p	0.111	a	a	a	a

DE - dependence entropy; DN - dependence non-uniformity; DNN - dependence non-uniformity normalized; DV - dependence variance; GLN - gray level non-uniformity; GLV - gray level variance; HGLE - high gray level emphasis; LDE - large dependence emphasis; LDHGLE - large dependence high gray level emphasis; LDLGLE - large dependence low gray level emphasis; LGLE - low gray level emphasis; SDE - small dependence emphasis; SDHGLE - small dependence high gray level emphasis; SDLGLE - small dependence low gray level emphasis. Lower case letters (a–d) indicate differences between classes for p < 0.05 independently for each feature.

Table S35. The values (mean ±SD) of features of *Gray Level Dependence Matrix (GLDM)* of output images, filtrated by *DiscreteGaussian* filter, compared between four classes (0-3) of the Equine Odontoclastic Tooth Resorption and Hypercementosis (EOTRH) syndrome. When features differed significantly ($p<0.05$).

Feature		EOTRH	0	1	2	3
**DE**		Mean	6.24	6.08	6.22	6.32
		SD	0.28	0.35	0.36	0.26
	p	< 0.0001	a	b	a	a
**DN**		Mean	73.6	69.3	83.5	79.7
		SD	27.6	37.8	43.9	27.3
	p	< 0.0001	a	b	a	a
**DNN**		Mean	0.04	0.04	0.04	0.04
		SD	0.01	0.01	0.01	0.01
	p	0.8937	a	a	a	a
**DV**		Mean	64.7	75.3	68.1	67.7
		SD	23.8	25.1	20.0	33.3
	p	0.001	a	b	ab	ab
**GLN**		Mean	64.7	75.3	68.1	67.7
		SD	23.8	25.1	20.0	33.3
	p	0.0323	ab	a	b	b
**GLV**		Mean	0.72	0.77	0.85	1.16
		SD	0.25	0.28	0.27	0.60
	p	< 0.0001	a	ab	bc	c
**HGLE**		Mean	11.4	13.9	14.6	18.5
		SD	3.6	4.0	4.4	6.3
	p	< 0.0001	a	b	b	c
**LDE**		Mean	309.1	302.3	292.5	284.1
		SD	98.7	91.1	85.5	131.3
	p	0.3772	a	a	a	a
**LDHGLE**		Mean	3371	4475	4588	5519
		SD	1515	1901	1824	2562
	p	< 0.0001	a	b	b	b
**LDLGLE**		Mean	39.4	26.1	24.8	20.5
		SD	21.1	15.2	12.8	14.9
	p	< 0.0001	a	b	bc	c
**LGLE**		Mean	0.15	0.11	0.11	0.09
		SD	0.06	0.04	0.04	0.04
	p	< 0.0001	a	b	bc	c
**SDE**		Mean	0.03	0.04	0.04	0.04
		SD	0.01	0.01	0.01	0.02
	p	< 0.0001	a	b	b	b
**SDHGLE**		Mean	0.37	0.45	0.43	0.66
		SD	0.17	0.22	0.22	0.48
	p	< 0.0001	a	bc	ab	c
**SDLGLE**		Mean	0.008	0.009	0.009	0.009
		SD	0.004	0.004	0.004	0.005
	p	0.3411	a	a	a	a

DE - dependence entropy; DN - dependence non-uniformity; DNN - dependence non-uniformity normalized; DV - dependence variance; GLN - gray level non-uniformity; GLV - gray level variance; HGLE - high gray level emphasis; LDE - large dependence emphasis; LDHGLE - large dependence high gray level emphasis; LDLGLE - large dependence low gray level emphasis; LGLE - low gray level emphasis; SDE - small dependence emphasis; SDHGLE - small dependence high gray level emphasis; SDLGLE - small dependence low gray level emphasis. Lower case letters (a–c) indicate differences between classes for $p < 0.05$ independently for each feature.

Table S36. The values (mean ±SD) of features of *Gray Level Dependence Matrix (GLDM)* of output images, filtrated by *SmoothingRecursiveGaussian* filter, compared between four classes (0-3) of the Equine Odontoclastic Tooth Resorption and Hypercementosis (EOTRH) syndrome. When features differed significantly (p<0.05).

Feature		EOTRH	0	1	2	3
**DE**		Mean	6.24	6.07	6.22	6.33
		SD	0.27	0.36	0.37	0.26
	p	< 0.0001	a	b	a	a
**DN**		Mean	73.4	69.3	83.4	79.5
		SD	27.6	37.9	44.0	27.5
	p	< 0.0001	a	b	a	a
**DNN**		Mean	0.04	0.04	0.04	0.04
		SD	0.01	0.01	0.01	0.01
	p	0.8781	a	a	a	a
**DV**		Mean	65.2	76.0	68.8	68.1
		SD	23.9	25.3	20.1	33.3
	p	0.0012	a	b	ab	ab
**GLN**		Mean	683.8	643.8	724.5	615.8
		SD	318.8	374.0	437.0	310.1
	p	0.0306	ab	a	b	a
**GLV**		Mean	0.72	0.77	0.85	1.15
		SD	0.24	0.28	0.27	0.60
	p	< 0.0001	a	ab	bc	c
**HGLE**		Mean	11.2	13.8	14.5	18.4
		SD	3.9	3.8	4.6	6.4
	p	< 0.0001	a	b	b	c
**LDE**		Mean	312.0	304.3	294.4	285.6
		SD	99.2	91.2	86.1	131.1
	p	0.3672	a	a	a	a
**LDHGLE**		Mean	3374	4475	4597	5526
		SD	1641	1901	1943	2624
	p	< 0.0001	a	b	b	b
**LDLGLE**		Mean	40.8	26.3	26.0	20.7
		SD	21.3	15.0	15.2	14.8
	p	< 0.0001	a	b	bc	c
**LGLE**		Mean	0.15	0.11	0.11	0.09
		SD	0.06	0.04	0.05	0.04
	p	< 0.0001	a	b	bc	c
**SDE**		Mean	0.03	0.04	0.04	0.04
		SD	0.01	0.01	0.01	0.02
	p	< 0.0001	a	b	b	b
**SDHGLE**		Mean	0.37	0.45	0.43	0.67
		SD	0.17	0.21	0.24	0.48
	p	0.0001	a	ab	a	b
**SDLGLE**		Mean	0.008	0.009	0.009	0.009
		SD	0.004	0.004	0.004	0.005
	p	0.2527	a	a	a	a

DE - dependence entropy; DN - dependence non-uniformity; DNN - dependence non-uniformity normalized; DV - dependence variance; GLN - gray level non-uniformity; GLV - gray level variance; HGLE - high gray level emphasis; LDE - large dependence emphasis; LDHGLE - large dependence high gray level emphasis; LDLGLE - large dependence low gray level emphasis; LGLE - low gray level emphasis; SDE - small dependence emphasis; SDHGLE - small dependence high gray level emphasis; SDLGLE - small dependence low gray level emphasis. Lower case letters (a–c) indicate differences between classes for p < 0.05 independently for each feature.

Table S37. The values (mean ±SD) of features of *Gray Level Run Length Matrix (GLRLM)* of output images, filtrated by *Mean* filter, compared between four classes (0-3) of the Equine Odontoclastic Tooth Resorption and Hypercementosis (EOTRH) syndrome. When features differed significantly (p<0.05).

Feature		EOTRH	0	1	2	3
**GLN**		Mean	163.8	134.2	154.9	136.6
		SD	187.0	65.2	78.3	50.0
	p	0.0014	a	b	a	ab
**GLNN**		Mean	0.30	0.28	0.27	0.25
		SD	0.04	0.03	0.03	0.04
	p	< 0.0001	a	b	b	c
**GLV**		Mean	0.96	1.04	1.07	1.32
		SD	0.47	0.27	0.29	0.49
	p	< 0.0001	a	b	b	c
**HGLRE**		Mean	12.3	13.4	14.2	17.3
		SD	4.5	3.9	4.1	5.9
	p	< 0.0001	a	b	b	c
**LRE**		Mean	46.1	49.8	46.7	39.4
		SD	34.2	23.7	22.7	23.0
	p	0.0101	ab	a	ab	b
**LRHGLE**		Mean	515.4	732.3	751.2	726.6
		SD	317.8	442.5	453.3	406.9
	p	< 0.0001	a	b	b	b
**LRLGLE**		Mean	7.93	4.68	4.24	3.04
		SD	25.48	2.81	2.82	2.07
	p	< 0.0001	a	ab	b	c
**LGLRE**		Mean	0.15	0.14	0.13	0.11
		SD	0.06	0.05	0.05	0.04
	p	< 0.0001	a	ab	bc	c
**RE**		Mean	4.89	4.77	4.91	4.97
		SD	0.31	0.30	0.29	0.24
	p	< 0.0001	a	b	a	a
**RLN**		Mean	108.9	89.6	96.7	104.6
		SD	249.2	43.1	45.4	56.7
	p	0.1997	a	a	a	a
**RLNN**		Mean	0.16	0.17	0.16	0.18
		SD	0.03	0.03	0.03	0.06
	p	< 0.0001	a	b	a	ab
**RP**		Mean	0.26	0.27	0.27	0.28
		SD	0.05	0.04	0.04	0.08
	p	0.0877	a	a	a	a
**RV**		Mean	23.6	26.0	22.7	18.8
		SD	28.1	15.6	14.9	12.8
	p	0.0007	a	b	ab	a
**SRE**		Mean	0.32	0.35	0.33	0.35
		SD	0.05	0.05	0.05	0.09
	p	< 0.0001	a	b	a	ab
**SRHGLE**		Mean	3.85	4.07	4.14	5.75
		SD	1.81	1.54	1.44	3.46
	p	0.0019	a	ab	ab	b
**SRLGLE**		Mean	0.05	0.06	0.05	0.05
		SD	0.02	0.03	0.02	0.02
	p	< 0.0001	a	b	a	a

GLN - gray level non-uniformity; GLNN - gray level non-uniformity normalized; GLV - gray level variance; HGLRE - high gray level run emphasis; LRE - long run emphasis; LRHGLE - long run high gray level emphasis; LRLGLE - long run low gray level emphasis; LGLRE - low gray level run emphasis; RE - run entropy; RLN - run length non-uniformity; RLNN - run length non-uniformity normalized; RP - run percentage; RV - run variance; SRE - short run emphasis; SRHGLE - short run high gray level emphasis; SRLGLE - short run low gray level emphasis. Lower case letters (a–c) indicate differences between classes for p < 0.05 independently for each feature.

Table S38. The values (mean ±SD) of features of *Gray Level Run Length Matrix (GLRLM)* of output images, filtrated by *Median* filter, compared between four classes (0-3) of the Equine Odontoclastic Tooth Resorption and Hypercementosis (EOTRH) syndrome. When features differed significantly (p<0.05).

Feature	EOTRH	0	1	2	3
**GLN**	Mean	143.4	131.7	154.0	134.3
	SD	49.3	65.0	78.6	49.5
p	0.0009	a	b	a	ab
**GLNN**	Mean	0.29	0.28	0.27	0.25
	SD	0.04	0.03	0.03	0.04
p	< 0.0001	a	b	b	c
**GLV**	Mean	0.98	1.08	1.10	1.38
	SD	0.26	0.29	0.30	0.54
p	< 0.0001	a	b	b	c
**HGLRE**	Mean	12.4	14.0	14.5	17.7
	SD	3.6	3.9	4.2	6.5
p	< 0.0001	a	b	b	c
**LRE**	Mean	39.4	43.5	41.7	36.8
	SD	17.2	18.2	20.2	20.7
p	0.053	a	a	a	a
**LRHGLE**	Mean	459.0	659.6	685.2	696.1
	SD	221.1	349.0	417.7	409.0
p	< 0.0001	a	b	b	b
**LRLGLE**	Mean	4.95	3.97	3.75	2.87
	SD	3.02	2.20	2.41	1.92
p	< 0.0001	a	ab	bc	c
**LGLRE**	Mean	0.15	0.13	0.12	0.11
	SD	0.06	0.05	0.05	0.04
p	0.0002	a	ab	bc	c
**RE**	Mean	4.90	4.82	4.94	5.01
	SD	0.24	0.28	0.27	0.21
p	< 0.0001	a	b	ac	c
**RLN**	Mean	83.6	84.6	94.3	98.6
	SD	33.5	42.7	45.5	48.9
p	0.0624	a	a	a	a
**RLNN**	Mean	0.16	0.17	0.16	0.17
	SD	0.03	0.03	0.03	0.05
p	0.006	a	b	ab	ab
**RP**	Mean	0.26	0.27	0.26	0.28
	SD	0.04	0.04	0.04	0.07
p	0.4159	a	a	a	a
**RV**	Mean	19.0	22.3	20.0	17.3
	SD	10.9	11.9	13.1	11.3
p	0.0027	a	b	ab	b
**SRE**	Mean	0.32	0.34	0.32	0.34
	SD	0.05	0.04	0.05	0.08
p	0.0071	a	b	a	ab
**SRHGLE**	Mean	3.96	4.22	4.20	5.76
	SD	1.58	1.61	1.42	3.32
p	0.0106	a	ab	ab	b
**SRLGLE**	Mean	0.05	0.06	0.05	0.04
	SD	0.02	0.02	0.02	0.02
p	0.0018	ab	a	ab	b

GLN - gray level non-uniformity; GLNN - gray level non-uniformity normalized; GLV - gray level variance; HGLRE - high gray level run emphasis; LRE - long run emphasis; LRHGLE - long run high gray level emphasis; LRLGLE - long run low gray level emphasis; LGLRE - low gray level run emphasis; RE - run entropy; RLN - run length non-uniformity; RLNN - run length non-uniformity normalized; RP - run percentage; RV - run variance; SRE - short run emphasis; SRHGLE - short run high gray level emphasis; SRLGLE - short run low gray level emphasis. Lower case letters (a–c) indicate differences between classes for p < 0.05 independently for each feature.

Table S39. The values (mean ±SD) of features of *Gray Level Run Length Matrix (GLRLM)* of output images, filtrated by *Normalize* filter, compared between four classes (0-3) of the Equine Odontoclastic Tooth Resorption and Hypercementosis (EOTRH) syndrome. When features differed significantly (p<0.05).

Feature		EOTRH	0	1	2	3
**GLN**		Mean	110.1	88.3	91.0	82.8
		SD	206.7	29.1	31.4	20.1
	p	0.0068	a	b	ab	b
**GLNN**		Mean	0.99	0.97	0.95	0.86
		SD	0.05	0.08	0.10	0.17
	p	< 0.0001	a	b	b	c
**GLV**		Mean	0.00	0.02	0.03	0.07
		SD	0.03	0.04	0.05	0.08
	p	< 0.0001	a	b	b	c
**HGLRE**		Mean	1.26	1.68	2.04	2.49
		SD	0.83	1.20	1.35	1.28
	p	< 0.0001	a	b	bc	c
**LRE**		Mean	1055	1192	1332	1060
		SD	538	913	918	712
	p	0.0599	a	a	a	a
**LRHGLE**		Mean	1364	2396	2956	2724
		SD	1251	3550	3189	2820
	p	< 0.0001	a	a	b	b
**LRLGLE**		Mean	977.6	890.4	926.0	644.7
		SD	555.6	675.7	863.6	602.0
	p	0.0002	a	a	a	b
**LGLRE**		Mean	0.93	0.83	0.74	0.63
		SD	0.21	0.30	0.34	0.32
	p	< 0.0001	a	b	bc	c
**RE**		Mean	4.53	4.21	4.24	4.47
		SD	0.39	0.41	0.50	0.31
	p	< 0.0001	a	b	b	a
**RLN**		Mean	9.11	7.43	7.66	7.41
		SD	35.86	3.91	2.52	4.07
	p	< 0.0001	a	b	c	bc
**RLNN**		Mean	0.06	0.08	0.08	0.07
		SD	0.02	0.03	0.03	0.02
	p	< 0.0001	a	b	b	b
**RP**		Mean	0.05	0.06	0.05	0.06
		SD	0.01	0.01	0.01	0.02
	p	< 0.0001	a	b	a	a
**RV**		Mean	218.3	257.8	258.4	223.0
		SD	171.7	259.5	220.4	147.0
	p	0.9374	a	a	a	a
**SRE**		Mean	0.03	0.03	0.04	0.08
		SD	0.03	0.04	0.05	0.09
	p	0.0239	ab	ab	a	b
**SRHGLE**		Mean	0.04	0.05	0.06	0.14
		SD	0.07	0.09	0.10	0.16
	p	0.0649	a	a	a	a
**SRLGLE**		Mean	0.02	0.03	0.03	0.06
		SD	0.02	0.03	0.04	0.07
	p	0.0074	ab	ab	a	b

GLN - gray level non-uniformity; GLNN - gray level non-uniformity normalized; GLV - gray level variance; HGLRE - high gray level run emphasis; LRE - long run emphasis; LRHGLE - long run high gray level emphasis; LRLGLE - long run low gray level emphasis; LGLRE - low gray level run emphasis; RE - run entropy; RLN - run length non-uniformity; RLNN - run length non-uniformity normalized; RP - run percentage; RV - run variance; SRE - short run emphasis; SRHGLE - short run high gray level emphasis; SRLGLE - short run low gray level emphasis. Lower case letters (a–c) indicate differences between classes for p < 0.05 independently for each feature.

Table S40. The values (mean ±SD) of features of *Gray Level Run Length Matrix (GLRLM)* of output images, filtrated by *Bilateral* filter, compared between four classes (0-3) of the Equine Odontoclastic Tooth Resorption and Hypercementosis (EOTRH) syndrome. When features differed significantly (p<0.05).

Feature		EOTRH	0	1	2	3
**GLN**		Mean	98.5	97.7	112.6	105.9
		SD	28.9	41.5	49.2	36.6
	p	0.0022	ab	a	b	b
**GLNN**		Mean	0.36	0.33	0.32	0.29
		SD	0.05	0.04	0.04	0.04
	p	< 0.0001	a	b	b	c
**GLV**		Mean	0.63	0.76	0.77	0.96
		SD	0.18	0.21	0.24	0.30
	p	< 0.0001	a	b	b	c
**HGLRE**		Mean	7.44	9.23	9.86	12.17
		SD	2.57	2.87	3.48	3.80
	p	< 0.0001	a	b	b	c
**LRE**		Mean	134.8	131.1	120.6	99.0
		SD	64.0	83.3	56.5	67.6
	p	0.0024	a	a	a	b
**LRHGLE**		Mean	967	1446	1413	1437
		SD	577	1190	902	1116
	p	< 0.0001	a	b	b	b
**LRLGLE**		Mean	26.4	17.0	15.0	9.5
		SD	16.8	15.0	10.5	7.5
	p	< 0.0001	a	b	b	c
**LGLRE**		Mean	0.25	0.21	0.19	0.15
		SD	0.10	0.10	0.09	0.06
	p	< 0.0001	a	b	b	c
**RE**		Mean	5.12	4.88	5.04	5.09
		SD	0.31	0.39	0.42	0.27
	p	< 0.0001	a	b	a	a
**RLN**		Mean	27.9	36.7	39.3	48.9
		SD	11.1	18.0	15.4	31.5
	p	< 0.0001	a	b	b	b
**RLNN**		Mean	0.09	0.12	0.11	0.12
		SD	0.02	0.03	0.03	0.04
	p	< 0.0001	a	b	b	b
**RP**		Mean	0.15	0.17	0.17	0.19
		SD	0.03	0.04	0.04	0.06
	p	< 0.0001	a	b	b	b
**RV**		Mean	66.0	70.4	59.7	48.8
		SD	36.0	53.6	31.0	37.2
	p	0.002	a	b	a	a
**SRE**		Mean	0.23	0.27	0.26	0.28
		SD	0.06	0.06	0.06	0.08
	p	< 0.0001	a	b	b	b
**SRHGLE**		Mean	1.53	1.92	2.11	2.97
		SD	0.76	0.84	1.10	1.79
	p	< 0.0001	a	b	b	c
**SRLGLE**		Mean	0.08	0.08	0.07	0.06
		SD	0.04	0.04	0.03	0.02
	p	< 0.0001	a	a	ab	b

GLN - gray level non-uniformity; GLNN - gray level non-uniformity normalized; GLV - gray level variance; HGLRE - high gray level run emphasis; LRE - long run emphasis; LRHGLE - long run high gray level emphasis; LRLGLE - long run low gray level emphasis; LGLRE - low gray level run emphasis; RE - run entropy; RLN - run length non-uniformity; RLNN - run length non-uniformity normalized; RP - run percentage; RV - run variance; SRE - short run emphasis; SRHGLE - short run high gray level emphasis; SRLGLE - short run low gray level emphasis. Lower case letters (a–c) indicate differences between classes for p < 0.05 independently for each feature.

Table S41. The values (mean ±SD) of features of *Gray Level Run Length Matrix (GLRLM)* of output images, filtrated by *Binomial* filter, compared between four classes (0-3) of the Equine Odontoclastic Tooth Resorption and Hypercementosis (EOTRH) syndrome. When features differed significantly (p<0.05).

Feature		EOTRH	0	1	2	3
**GLN**		Mean	151.8	138.9	162.5	142.1
		SD	51.9	69.1	84.4	52.8
	p	0.0008	a	b	a	ab
**GLNN**		Mean	0.29	0.28	0.27	0.25
		SD	0.04	0.03	0.03	0.04
	p	< 0.0001	a	b	b	c
**GLV**		Mean	0.95	1.06	1.09	1.35
		SD	0.25	0.28	0.29	0.51
	p	< 0.0001	a	b	b	c
**HGLRE**		Mean	12.2	13.8	14.4	17.7
		SD	3.6	3.8	4.2	6.0
	p	< 0.0001	a	b	b	c
**LRE**		Mean	38.7	43.9	41.2	35.4
		SD	17.5	19.8	20.8	20.7
	p	< 0.0001	a	b	b	c
**LRHGLE**		Mean	445.2	657.9	671.1	659.2
		SD	222.1	363.2	418.1	367.1
	p	< 0.0001	a	b	b	b
**LRLGLE**		Mean	4.84	3.99	3.72	2.71
		SD	2.86	2.30	2.45	1.82
	p	< 0.0001	a	ac	b	c
**LGLRE**		Mean	0.15	0.13	0.12	0.11
		SD	0.06	0.04	0.05	0.04
	p	< 0.0001	a	ab	bc	c
**RE**		Mean	4.83	4.75	4.86	4.93
		SD	0.26	0.29	0.27	0.24
	p	< 0.0001	a	b	a	a
**RLN**		Mean	94.9	97.0	108.7	116.8
		SD	39.3	50.5	54.0	65.6
	p	0.0712	a	a	a	a
**RLNN**		Mean	0.17	0.18	0.17	0.19
		SD	0.03	0.03	0.03	0.06
	p	0.0064	a	b	ab	ab
**RP**		Mean	0.27	0.28	0.28	0.30
		SD	0.05	0.04	0.04	0.08
	p	0.3833	a	a	a	a
**RV**		Mean	18.9	22.9	20.2	17.0
		SD	11.1	13.3	13.8	11.6
	p	0.0007	a	b	ab	a
**SRE**		Mean	0.34	0.36	0.35	0.37
		SD	0.05	0.05	0.05	0.09
	p	0.0122	a	b	ab	ab
**SRHGLE**		Mean	4.11	4.45	4.56	6.32
		SD	1.61	1.66	1.55	3.80
	p	0.0015	a	ab	ab	b
**SRLGLE**		Mean	0.06	0.06	0.05	0.05
		SD	0.02	0.02	0.02	0.02
	p	0.0003	a	a	ab	b

GLN - gray level non-uniformity; GLNN - gray level non-uniformity normalized; GLV - gray level variance; HGLRE - high gray level run emphasis; LRE - long run emphasis; LRHGLE - long run high gray level emphasis; LRLGLE - long run low gray level emphasis; LGLRE - low gray level run emphasis; RE - run entropy; RLN - run length non-uniformity; RLNN - run length non-uniformity normalized; RP - run percentage; RV - run variance; SRE - short run emphasis; SRHGLE - short run high gray level emphasis; SRLGLE - short run low gray level emphasis. Lower case letters (a–c) indicate differences between classes for p < 0.05 independently for each feature.

Table S42. The values (mean ±SD) of features of *Gray Level Run Length Matrix (GLRLM)* of output images, filtrated by *CurvatureFlow* filter, compared between four classes (0-3) of the Equine Odontoclastic Tooth Resorption and Hypercementosis (EOTRH) syndrome. When features differed significantly (p<0.05).

Feature		EOTRH	0	1	2	3
**GLN**		Mean	171.4	157.2	188.6	163.2
		SD	57.4	78.4	98.9	61.7
	p	0.0002	a	b	ab	a
**GLNN**		Mean	0.28	0.27	0.26	0.24
		SD	0.03	0.03	0.03	0.04
	p	< 0.0001	a	ab	bc	c
**GLV**		Mean	1.05	1.13	1.18	1.49
		SD	0.27	0.30	0.31	0.62
	p	< 0.0001	a	ab	b	c
**HGLRE**		Mean	13.8	15.4	16.0	20.0
		SD	3.8	4.2	4.7	7.4
	p	< 0.0001	a	b	b	c
**LRE**		Mean	24.6	27.1	25.7	22.8
		SD	11.0	10.8	12.9	12.7
	p	0.0127	ab	a	ab	b
**LRHGLE**		Mean	321.5	443.2	455.3	463.5
		SD	159.8	225.6	272.7	252.6
	p	< 0.0001	a	b	b	b
**LRLGLE**		Mean	2.61	2.27	2.06	1.67
		SD	1.37	1.20	1.18	1.22
	p	< 0.0001	a	ab	bc	c
**LGLRE**		Mean	0.13	0.11	0.11	0.10
		SD	0.04	0.04	0.04	0.04
	p	< 0.0001	a	ab	bc	c
**RE**		Mean	4.67	4.63	4.70	4.76
		SD	0.24	0.26	0.24	0.21
	p	0.0005	ab	a	ab	b
**RLN**		Mean	135.2	132.0	159.9	167.9
		SD	49.3	69.5	81.9	89.9
	p	0.0005	ab	a	b	b
**RLNN**		Mean	0.21	0.21	0.21	0.23
		SD	0.04	0.03	0.03	0.07
	p	0.5471	a	a	a	a
**RP**		Mean	0.32	0.33	0.33	0.35
		SD	0.05	0.05	0.04	0.09
	p	0.8334	a	a	a	a
**RV**		Mean	12.1	14.0	12.8	11.2
		SD	6.8	7.0	8.7	7.3
	p	0.0029	a	b	ab	a
**SRE**		Mean	0.42	0.43	0.43	0.45
		SD	0.05	0.05	0.04	0.09
	p	0.4045	a	a	a	a
**SRHGLE**		Mean	5.87	6.32	6.57	9.05
		SD	2.07	2.12	2.17	4.82
	p	< 0.0001	a	a	ab	b
**SRLGLE**		Mean	0.06	0.06	0.06	0.05
		SD	0.02	0.02	0.02	0.02
	p	0.0047	a	a	ab	b

GLN - gray level non-uniformity; GLNN - gray level non-uniformity normalized; GLV - gray level variance; HGLRE - high gray level run emphasis; LRE - long run emphasis; LRHGLE - long run high gray level emphasis; LRLGLE - long run low gray level emphasis; LGLRE - low gray level run emphasis; RE - run entropy; RLN - run length non-uniformity; RLNN - run length non-uniformity normalized; RP - run percentage; RV - run variance; SRE - short run emphasis; SRHGLE - short run high gray level emphasis; SRLGLE - short run low gray level emphasis. Lower case letters (a–c) indicate differences between classes for p < 0.05 independently for each feature.

Table S43. The values (mean ±SD) of features of *Gray Level Run Length Matrix (GLRLM)* of output images, filtrated by *LaplacianSharpening* filter, compared between four classes (0-3) of the Equine Odontoclastic Tooth Resorption and Hypercementosis (EOTRH) syndrome. When features differed significantly (p<0.05).

Feature		EOTRH	0	1	2	3
**GLN**		Mean	270.1	258.4	302.6	261.4
		SD	120.8	150.8	183.3	119.2
	p	0.002	a	b	a	ab
**GLNN**		Mean	0.21	0.21	0.20	0.19
		SD	0.03	0.03	0.03	0.04
	p	< 0.0001	a	a	bc	c
**GLV**		Mean	1.91	1.91	2.06	2.76
		SD	0.51	0.49	0.52	1.40
	p	< 0.0001	a	a	ab	b
**HGLRE**		Mean	33.2	37.1	39.0	47.9
		SD	9.1	11.6	11.5	13.8
	p	< 0.0001	a	b	b	c
**LRE**		Mean	3.38	3.29	3.32	3.08
		SD	0.67	0.63	0.87	0.89
	p	0.0769	a	a	a	a
**LRHGLE**		Mean	111.1	123.8	131.0	145.9
		SD	34.8	44.1	41.8	43.3
	p	< 0.0001	a	ab	bc	c
**LRLGLE**		Mean	0.14	0.12	0.12	0.10
		SD	0.06	0.06	0.07	0.06
	p	< 0.0001	a	b	b	c
**LGLRE**		Mean	0.05	0.04	0.04	0.04
		SD	0.02	0.02	0.02	0.01
	p	< 0.0001	a	ab	bc	c
**RE**		Mean	3.81	3.76	3.81	3.87
		SD	0.13	0.14	0.13	0.14
	p	< 0.0001	a	b	ac	c
**RLN**		Mean	669.8	657.7	807.1	770.9
		SD	263.5	396.2	490.6	287.7
	p	< 0.0001	ab	a	b	b
**RLNN**		Mean	0.52	0.53	0.53	0.56
		SD	0.04	0.04	0.05	0.07
	p	0.013	a	ab	ab	b
**RP**		Mean	0.66	0.67	0.67	0.69
		SD	0.04	0.04	0.05	0.07
	p	0.0541	a	a	a	a
**RV**		Mean	1.03	0.99	1.02	0.91
		SD	0.34	0.33	0.49	0.46
	p	0.1383	a	a	a	a
**SRE**		Mean	0.75	0.75	0.75	0.77
		SD	0.03	0.03	0.03	0.05
	p	0.0095	a	ab	ab	b
**SRHGLE**		Mean	24.8	27.8	29.3	37.0
		SD	7.1	9.0	9.1	11.9
	p	< 0.0001	a	b	b	c
**SRLGLE**		Mean	0.04	0.03	0.03	0.03
		SD	0.01	0.01	0.01	0.01
	p	0.0003	a	ab	bc	ac

GLN - gray level non-uniformity; GLNN - gray level non-uniformity normalized; GLV - gray level variance; HGLRE - high gray level run emphasis; LRE - long run emphasis; LRHGLE - long run high gray level emphasis; LRLGLE - long run low gray level emphasis; LGLRE - low gray level run emphasis; RE - run entropy; RLN - run length non-uniformity; RLNN - run length non-uniformity normalized; RP - run percentage; RV - run variance; SRE - short run emphasis; SRHGLE - short run high gray level emphasis; SRLGLE - short run low gray level emphasis. Lower case letters (a–c) indicate differences between classes for p < 0.05 independently for each feature.

Table S44. The values (mean ±SD) of features of *Gray Level Run Length Matrix (GLRLM)* of output images, filtrated by *DiscreteGaussian* filter, compared between four classes (0-3) of the Equine Odontoclastic Tooth Resorption and Hypercementosis (EOTRH) syndrome. When features differed significantly (p<0.05).

Feature		EOTRH	0	1	2	3
**GLN**		Mean	141.8	130.7	151.0	133.0
		SD	48.0	63.4	76.2	48.8
	p	0.0019	a	b	a	ab
**GLNN**		Mean	0.30	0.28	0.28	0.26
		SD	0.04	0.03	0.04	0.04
	p	< 0.0001	a	b	b	c
**GLV**		Mean	0.89	1.02	1.05	1.29
		SD	0.23	0.26	0.29	0.46
	p	< 0.0001	a	b	b	c
**HGLRE**		Mean	11.4	12.8	13.4	16.7
		SD	3.5	3.5	3.9	5.7
	p	< 0.0001	a	b	b	c
**LRE**		Mean	47.3	54.8	50.3	42.9
		SD	20.6	27.0	23.7	25.3
	p	0.0063	ab	a	ab	b
**LRHGLE**		Mean	521.5	791.3	763.5	775.5
		SD	265.5	512.9	433.0	449.9
	p	< 0.0001	a	b	b	b
**LRLGLE**		Mean	6.27	5.24	4.85	3.50
		SD	3.56	3.05	3.06	2.55
	p	< 0.0001	a	ab	b	c
**LGLRE**		Mean	0.16	0.14	0.14	0.11
		SD	0.07	0.05	0.05	0.05
	p	< 0.0001	a	ab	bc	c
**RE**		Mean	4.91	4.78	4.93	4.99
		SD	0.26	0.31	0.30	0.24
	p	< 0.0001	a	b	a	a
**RLN**		Mean	76.0	82.7	89.6	95.8
		SD	29.8	39.4	42.0	50.1
	p	0.0574	a	a	a	a
**RLNN**		Mean	0.15	0.17	0.16	0.17
		SD	0.03	0.03	0.03	0.05
	p	< 0.0001	a	b	a	ab
**RP**		Mean	0.25	0.26	0.26	0.27
		SD	0.04	0.04	0.04	0.07
	p	0.0368	a	b	ab	ab
**RV**		Mean	22.9	28.7	24.2	20.4
		SD	13.2	18.1	15.1	14.2
	p	0.0003	a	b	ab	a
**SRE**		Mean	0.30	0.33	0.31	0.33
		SD	0.05	0.05	0.05	0.08
	p	< 0.0001	a	b	a	ab
**SRHGLE**		Mean	3.30	3.66	3.72	5.22
		SD	1.24	1.33	1.33	3.14
	p	0.0002	a	b	ab	a
**SRLGLE**		Mean	0.06	0.06	0.05	0.05
		SD	0.03	0.03	0.02	0.02
	p	< 0.0001	ab	a	b	b

GLN - gray level non-uniformity; GLNN - gray level non-uniformity normalized; GLV - gray level variance; HGLRE - high gray level run emphasis; LRE - long run emphasis; LRHGLE - long run high gray level emphasis; LRLGLE - long run low gray level emphasis; LGLRE - low gray level run emphasis; RE - run entropy; RLN - run length non-uniformity; RLNN - run length non-uniformity normalized; RP - run percentage; RV - run variance; SRE - short run emphasis; SRHGLE - short run high gray level emphasis; SRLGLE - short run low gray level emphasis. Lower case letters (a–c) indicate differences between classes for p < 0.05 independently for each feature.

Table S45. The values (mean ±SD) of features of *Gray Level Run Length Matrix (GLRLM)* of output images, filtrated by *SmoothingRecursiveGaussian* filter, compared between four classes (0-3) of the Equine Odontoclastic Tooth Resorption and Hypercementosis (EOTRH) syndrome. When features differed significantly (p<0.05).

Feature		EOTRH	0	1	2	3
**GLN**		Mean	139.3	128.7	147.8	130.5
		SD	47.3	62.0	73.9	47.9
	p	0.0028	a	b	a	ab
**GLNN**		Mean	0.30	0.28	0.28	0.26
		SD	0.04	0.03	0.04	0.04
	p	< 0.0001	a	b	b	c
**GLV**		Mean	0.89	1.02	1.04	1.29
		SD	0.23	0.26	0.28	0.45
	p	< 0.0001	a	b	b	c
**HGLRE**		Mean	11.2	12.7	13.3	16.6
		SD	3.7	3.4	4.0	5.8
	p	< 0.0001	a	b	b	c
**LRE**		Mean	49.7	57.7	53.4	44.9
		SD	21.3	28.7	25.2	26.1
	p	0.0082	ab	a	ab	b
**LRHGLE**		Mean	543.8	821.8	809.7	810.4
		SD	296.3	516.4	478.7	478.3
	p	< 0.0001	a	b	b	b
**LRLGLE**		Mean	6.81	5.59	5.33	3.67
		SD	3.88	3.42	3.47	2.61
	p	< 0.0001	a	b	bc	c
**LGLRE**		Mean	0.17	0.15	0.14	0.12
		SD	0.07	0.06	0.06	0.05
	p	< 0.0001	a	ab	bc	c
**RE**		Mean	4.92	4.79	4.94	5.00
		SD	0.27	0.32	0.31	0.25
	p	< 0.0001	a	b	a	a
**RLN**		Mean	73.2	80.9	85.8	92.1
		SD	29.2	38.1	39.1	47.9
	p	0.0514	a	a	a	A
**RLNN**		Mean	0.15	0.17	0.15	0.16
		SD	0.03	0.03	0.03	0.05
	p	< 0.0001	a	b	bc	c
**RP**		Mean	0.24	0.26	0.25	0.27
		SD	0.04	0.04	0.04	0.07
	p	0.0126	a	b	ab	ab
**RV**		Mean	23.9	30.0	25.5	21.1
		SD	13.7	18.9	16.1	14.5
	p	0.0004	a	b	ab	a
**SRE**		Mean	0.29	0.32	0.30	0.32
		SD	0.05	0.05	0.05	0.08
	p	< 0.0001	a	a	b	b
**SRHGLE**		Mean	3.13	3.47	3.49	4.97
		SD	1.26	1.13	1.32	3.14
	p	0.001	a	b	ab	b
**SRLGLE**		Mean	0.06	0.06	0.06	0.05
		SD	0.03	0.03	0.02	0.02
	p	< 0.0001	a	b	a	a

GLN - gray level non-uniformity; GLNN - gray level non-uniformity normalized; GLV - gray level variance; HGLRE - high gray level run emphasis; LRE - long run emphasis; LRHGLE - long run high gray level emphasis; LRLGLE - long run low gray level emphasis; LGLRE - low gray level run emphasis; RE - run entropy; RLN - run length non-uniformity; RLNN - run length non-uniformity normalized; RP - run percentage; RV - run variance; SRE - short run emphasis; SRHGLE - short run high gray level emphasis; SRLGLE - short run low gray level emphasis. Lower case letters (a–c) indicate differences between classes for p < 0.05 independently for each feature.

Table S46. The values (mean ±SD) of features of *Gray Level Size Zone Matrix (GLSZM)* of output images, filtrated by *Mean* filter, compared between four classes (0-3) of the Equine Odontoclastic Tooth Resorption and Hypercementosis (EOTRH) syndrome. When features differed significantly (p<0.05).

Feature		EOTRH	0	1	2	3
**GLN**		Mean	8.76	7.49	8.20	7.49
		SD	9.40	2.76	3.16	2.72
	p	0.1896	a	a	a	a
**GLNN**		Mean	0.25	0.25	0.24	0.22
		SD	0.05	0.05	0.04	0.05
	p	0.0001	a	a	a	b
**GLV**		Mean	1.60	1.84	1.83	2.22
		SD	0.46	0.50	0.51	0.72
	p	< 0.0001	a	b	b	c
**HGLZE**		Mean	11.7	11.7	12.3	15.3
		SD	4.9	3.9	4.0	5.9
	p	< 0.0001	a	a	a	b
**LAE**		Mean	52245	33076	38086	32819
		SD	179232	30322	35189	28353
	p	0.1156	a	a	a	a
**LAHGLE**		Mean	479010	502080	598102	580243
		SD	908904	573944	655116	490186
	p	0.005	a	ab	b	ab
**LALGLE**		Mean	18935	2699	3045	2281
		SD	153844	2403	2976	2354
	p	< 0.0001	a	b	b	b
**LGLZE**		Mean	0.23	0.24	0.22	0.19
		SD	0.10	0.10	0.09	0.08
	p	0.0031	ab	a	ab	b
**SZN**		Mean	3.95	3.27	3.69	3.43
		SD	4.04	1.52	1.74	1.63
	p	0.1142	a	a	a	a
**SZNN**		Mean	0.12	0.11	0.11	0.10
		SD	0.05	0.04	0.03	0.03
	p	0.1671	a	a	a	a
**SAE**		Mean	0.27	0.26	0.26	0.25
		SD	0.10	0.09	0.08	0.08
	p	0.6134	a	a	a	a
**SAHGLE**		Mean	2.77	2.41	2.82	3.30
		SD	1.80	1.37	1.60	1.76
	p	0.0009	ab	a	ab	b
**SALGLE**		Mean	0.074	0.082	0.071	0.060
		SD	0.053	0.054	0.045	0.042
	p	0.0059	ab	a	ab	b
**ZE**		Mean	4.42	4.39	4.50	4.61
		SD	0.54	0.48	0.46	0.51
	p	0.007	ab	a	ab	b
**ZP**		Mean	0.017	0.018	0.017	0.019
		SD	0.005	0.005	0.006	0.008
	p	0.0652	a	a	a	a
**ZV**		Mean	47561	29110	33505	28232
		SD	177065	27925	32482	25265
	p	0.1364	a	a	a	a

GLN - gray level non-uniformity; GLNN - gray level non-uniformity normalized; GLV - gray level variance; HGLZE - high gray level zone emphasis; LAE - large area emphasis; LAHGLE - large area high gray level emphasis; LALGLE - large area low gray level emphasis; LGLZE - low gray level zone emphasis; SZN - size-zone non-uniformity; SZNN - size-zone non-uniformity normalized; SAE - small area emphasis; SAHGLE - small area high gray level emphasis; SALGLE - small area low gray level emphasis; ZE - zone entropy; ZP - zone percentage; ZV - zone variance. Lower case letters (a–c) indicate differences between classes for p < 0.05 independently for each feature.

Table S47. The values (mean ±SD) of features of *Gray Level Size Zone Matrix (GLSZM)* of output images, filtrated by *Median* filter, compared between four classes (0-3) of the Equine Odontoclastic Tooth Resorption and Hypercementosis (EOTRH) syndrome. When features differed significantly (p<0.05).

Feature		EOTRH	0	1	2	3
**GLN**		Mean	9.66	8.63	9.51	9.23
		SD	3.64	3.62	4.07	3.59
	p	0.0698	a	a	a	a
**GLNN**		Mean	0.25	0.24	0.23	0.22
		SD	0.05	0.04	0.04	0.04
	p	< 0.0001	a	ab	b	c
**GLV**		Mean	1.54	1.74	1.78	2.04
		SD	0.42	0.44	0.42	0.55
	p	< 0.0001	a	b	b	c
**HGLZE**		Mean	12.0	12.4	12.8	16.0
		SD	3.8	3.9	4.1	6.2
	p	0.0003	a	a	a	b
**LAE**		Mean	27554	25821	28001	25822
		SD	25022	24180	25779	25935
	p	0.3797				
**LAHGLE**		Mean	316958	402733	453463	471845
		SD	317481	449651	514848	526983
	p	0.0221	a	ab	b	ab
**LALGLE**		Mean	3063	2043	2224	1768
		SD	2874	1937	2232	1895
	p	< 0.0001	a	b	ab	b
**LGLZE**		Mean	0.21	0.21	0.20	0.17
		SD	0.08	0.08	0.08	0.07
	p	0.0313	ab	a	ab	b
**SZN**		Mean	4.04	3.42	3.98	3.82
		SD	2.51	1.74	2.31	2.15
	p	0.0753	a	a	a	a
**SZNN**		Mean	0.10	0.10	0.10	0.09
		SD	0.04	0.03	0.03	0.03
	p	0.1083	a	a	a	a
**SAE**		Mean	0.25	0.22	0.23	0.23
		SD	0.09	0.09	0.08	0.08
	p	0.0903	a	a	a	a
**SAHGLE**		Mean	2.59	2.16	2.50	3.23
		SD	1.39	1.24	1.43	2.01
	p	0.0002	ab	a	ab	b
**SALGLE**		Mean	0.06	0.06	0.06	0.05
		SD	0.03	0.04	0.03	0.03
	p	0.1269				
**ZE**		Mean	4.62	4.63	4.72	4.87
		SD	0.41	0.49	0.52	0.57
	p	0.011	a	a	ab	b
**ZP**		Mean	0.02	0.02	0.02	0.02
		SD	0.01	0.01	0.01	0.01
	p	0.3702	a	a	a	a
**ZV**		Mean	24240	22904	24766	22471
		SD	22759	22526	24008	23263
	p	0.4082	a	a	a	a

GLN - gray level non-uniformity; GLNN - gray level non-uniformity normalized; GLV - gray level variance; HGLZE - high gray level zone emphasis; LAE - large area emphasis; LAHGLE - large area high gray level emphasis; LALGLE - large area low gray level emphasis; LGLZE - low gray level zone emphasis; SZN - size-zone non-uniformity; SZNN - size-zone non-uniformity normalized; SAE - small area emphasis; SAHGLE - small area high gray level emphasis; SALGLE - small area low gray level emphasis; ZE - zone entropy; ZP - zone percentage; ZV - zone variance. Lower case letters (a–c) indicate differences between classes for p < 0.05 independently for each feature.

Table S48. The values (mean ±SD) of features of *Gray Level Size Zone Matrix (GLSZM)* of output images, filtrated by *Normalize* filter, compared between four classes (0-3) of the Equine Odontoclastic Tooth Resorption and Hypercementosis (EOTRH) syndrome. When features differed significantly (p<0.05).

Feature		EOTRH	0	1	2	3
**GLN**		Mean	2.74	1.51	1.94	3.48
		SD	17.35	1.94	2.48	4.73
	p	< 0.0001	a	ab	b	c
**GLNN**		Mean	0.96	0.90	0.86	0.80
		SD	0.13	0.18	0.20	0.20
	p	< 0.0001	a	b	bc	c
**GLV**		Mean	0.02	0.05	0.07	0.10
		SD	0.07	0.09	0.10	0.10
	p	< 0.0001	a	b	bc	c
**HGLZE**		Mean	1.11	1.25	1.37	1.47
		SD	0.35	0.49	0.57	0.56
	p	< 0.0001	a	b	bc	c
**LAE**		Mean	4576000	3432000	4192000	2465000
		SD	5772000	5734000	6413000	2430000
	p	< 0.0001	a	b	b	b
**LAHGLE**		Mean	5282000	5498000	7736000	4946000
		SD	6764000	9852000	12330000	5658000
	p	0.0001	a	b	a	ab
**LALGLE**		Mean	4400000	2916000	3306000	1845000
		SD	5760000	5568000	6182000	2413000
	p	< 0.0001	a	bc	b	c
**LGLZE**		Mean	0.97	0.94	0.91	0.88
		SD	0.09	0.12	0.14	0.14
	p	< 0.0001	a	b	bc	c
**SZN**		Mean	1.77	1.16	1.18	1.43
		SD	7.70	0.50	0.57	0.93
	p	< 0.0001	a	ab	bc	c
**SZNN**		Mean	0.95	0.86	0.77	0.60
		SD	0.17	0.26	0.31	0.35
	p	< 0.0001	a	ab	b	c
**SAE**		Mean	0.04	0.10	0.15	0.17
		SD	0.14	0.20	0.22	0.19
	p	< 0.0001	a	b	b	b
**SAHGLE**		Mean	0.05	0.11	0.15	0.17
		SD	0.16	0.23	0.22	0.20
	p	< 0.0001	a	b	b	b
**SALGLE**		Mean	0.04	0.10	0.15	0.17
		SD	0.14	0.20	0.22	0.19
	p	< 0.0001	a	b	b	b
**ZE**		Mean	0.14	0.35	0.58	1.09
		SD	0.48	0.70	0.85	1.06
	p	< 0.0001	a	ab	b	c
**ZP**		Mean	0.0007	0.0011	0.0012	0.0024
		SD	0.0004	0.0011	0.0013	0.0028
	p	< 0.0001	a	b	b	b
**ZV**		Mean	229110	427940	711449	526361
		SD	1429000	1334000	1654000	952263
	p	< 0.0001	a	b	bc	c

GLN - gray level non-uniformity; GLNN - gray level non-uniformity normalized; GLV - gray level variance; HGLZE - high gray level zone emphasis; LAE - large area emphasis; LAHGLE - large area high gray level emphasis; LALGLE - large area low gray level emphasis; LGLZE - low gray level zone emphasis; SZN - size-zone non-uniformity; SZNN - size-zone non-uniformity normalized; SAE - small area emphasis; SAHGLE - small area high gray level emphasis; SALGLE - small area low gray level emphasis; ZE - zone entropy; ZP - zone percentage; ZV - zone variance. Lower case letters (a–c) indicate differences between classes for p < 0.05 independently for each feature.

Table S49. The values (mean ±SD) of features of *Gray Level Size Zone Matrix (GLSZM)* of output images, filtrated by *Bilateral* filter, compared between four classes (0-3) of the Equine Odontoclastic Tooth Resorption and Hypercementosis (EOTRH) syndrome. When features differed significantly (p<0.05).

Feature		EOTRH	0	1	2	3
**GLN**		Mean	5.62	5.83	6.26	5.95
		SD	2.29	2.50	2.42	2.69
	p	0.1466	a	a	a	a
**GLNN**		Mean	0.36	0.34	0.32	0.28
		SD	0.09	0.09	0.08	0.06
	p	< 0.0001	a	ab	b	c
**GLV**		Mean	0.91	1.10	1.14	1.39
		SD	0.36	0.42	0.43	0.46
	p	< 0.0001	a	b	b	c
**HGLZE**		Mean	6.32	6.96	7.69	9.89
		SD	2.54	2.61	3.00	3.63
	p	< 0.0001	a	ab	b	c
**LAE**		Mean	116181	93684	106074	81256
		SD	104594	107367	121791	80949
	p	< 0.0001	a	b	ab	b
**LAHGLE**		Mean	837755	1058000	1194000	1117000
		SD	990116	1524000	1579000	1080000
	p	0.1242	a	a	a	a
**LALGLE**		Mean	21305	11032	12631	7331
		SD	18670	12589	16935	8063
	p	< 0.0001	a	b	b	b
**LGLZE**		Mean	0.40	0.37	0.33	0.27
		SD	0.17	0.16	0.15	0.12
	p	< 0.0001	a	ab	b	c
**SZN**		Mean	2.57	2.80	3.09	3.11
		SD	1.68	1.67	1.73	2.10
	p	0.0913	a	a	a	a
**SZNN**		Mean	0.16	0.16	0.15	0.13
		SD	0.06	0.06	0.05	0.04
	p	0.0188	a	a	ab	b
**SAE**		Mean	0.31	0.32	0.32	0.28
		SD	0.11	0.12	0.12	0.10
	p	0.1944	a	a	a	a
**SAHGLE**		Mean	1.60	1.64	2.02	2.49
		SD	1.05	1.15	1.41	1.73
	p	0.0015	a	a	ab	b
**SALGLE**		Mean	0.15	0.15	0.12	0.09
		SD	0.10	0.09	0.08	0.05
	p	< 0.0001	a	a	a	b
**ZE**		Mean	3.43	3.54	3.72	3.92
		SD	0.45	0.45	0.40	0.51
	p	< 0.0001	a	a	b	b
**ZP**		Mean	0.0087	0.0110	0.0106	0.0124
		SD	0.0037	0.0051	0.0047	0.0074
	p	0.0002	a	b	b	b
**ZV**		Mean	95224	77059	88168	66717
		SD	88547	90035	101806	68499
	p	0.0001	a	ab	b	ab

GLN - gray level non-uniformity; GLNN - gray level non-uniformity normalized; GLV - gray level variance; HGLZE - high gray level zone emphasis; LAE - large area emphasis; LAHGLE - large area high gray level emphasis; LALGLE - large area low gray level emphasis; LGLZE - low gray level zone emphasis; SZN - size-zone non-uniformity; SZNN - size-zone non-uniformity normalized; SAE - small area emphasis; SAHGLE - small area high gray level emphasis; SALGLE - small area low gray level emphasis; ZE - zone entropy; ZP - zone percentage; ZV - zone variance. Lower case letters (a–c) indicate differences between classes for p < 0.05 independently for each feature.

Table S50. The values (mean ±SD) of features of *Gray Level Size Zone Matrix (GLSZM)* of output images, filtrated by *Binomial* filter, compared between four classes (0-3) of the Equine Odontoclastic Tooth Resorption and Hypercementosis (EOTRH) syndrome. When features differed significantly (p<0.05).

Feature		EOTRH	0	1	2	3
**GLN**		Mean	8.83	8.02	9.05	8.51
		SD	2.98	3.17	3.87	3.07
	p	0.0271	ab	a	b	ab
**GLNN**		Mean	0.25	0.24	0.23	0.22
		SD	0.04	0.04	0.04	0.05
	p	< 0.0001	a	ab	bc	c
**GLV**		Mean	1.61	1.87	1.88	2.18
		SD	0.44	0.49	0.49	0.65
	p	< 0.0001	a	b	b	c
**HGLZE**		Mean	11.7	12.4	12.7	15.8
		SD	3.8	3.8	4.0	6.0
	p	< 0.0001	a	a	a	b
**LAE**		Mean	30155	28107	31400	27974
		SD	24166	24427	27548	25315
	p	0.2651	a	a	a	a
**LAHGLE**		Mean	344800	433552	498496	496306
		SD	317234	447143	509812	445154
	p	0.0204	a	ab	b	ab
**LALGLE**		Mean	3360	2224	2495	1908
		SD	2694	1980	2379	1966
	p	< 0.0001	a	b	b	b
**LGLZE**		Mean	0.23	0.22	0.21	0.18
		SD	0.10	0.08	0.09	0.07
	p	0.0092	a	a	ab	b
**SZN**		Mean	4.04	3.59	4.31	4.05
		SD	1.82	1.54	2.43	1.89
	p	0.0656	a	a	a	a
**SZNN**		Mean	0.11	0.11	0.11	0.10
		SD	0.04	0.03	0.03	0.03
	p	0.1034	a	a	a	a
**SAE**		Mean	0.28	0.27	0.26	0.25
		SD	0.08	0.08	0.08	0.08
	p	0.3543	a	a	a	a
**SAHGLE**		Mean	2.91	2.75	2.92	3.42
		SD	1.53	1.34	1.47	1.89
	p	0.1268	a	a	a	a
**SALGLE**		Mean	0.07	0.08	0.07	0.06
		SD	0.05	0.05	0.04	0.04
	p	0.0259	ab	ab	a	b
**ZE**		Mean	4.53	4.51	4.64	4.76
		SD	0.42	0.48	0.48	0.55
	p	0.0024	ab	ab	a	b
**ZP**		Mean	0.0194	0.0202	0.0195	0.0195
		SD	0.0063	0.0057	0.0063	0.0063
	p	0.2283	a	a	a	a
**ZV**		Mean	26648	24977	27888	24432
		SD	22420	22840	25783	22830
	p	0.2656	a	a	a	a

GLN - gray level non-uniformity; GLNN - gray level non-uniformity normalized; GLV - gray level variance; HGLZE - high gray level zone emphasis; LAE - large area emphasis; LAHGLE - large area high gray level emphasis; LALGLE - large area low gray level emphasis; LGLZE - low gray level zone emphasis; SZN - size-zone non-uniformity; SZNN - size-zone non-uniformity normalized; SAE - small area emphasis; SAHGLE - small area high gray level emphasis; SALGLE - small area low gray level emphasis; ZE - zone entropy; ZP - zone percentage; ZV - zone variance. Lower case letters (a–c) indicate differences between classes for $p < 0.05$ independently for each feature.

Table S51. The values (mean ±SD) of features of *Gray Level Size Zone Matrix (GLSZM)* of output images, filtrated by *CurvatureFlow* filter, compared between four classes (0-3) of the Equine Odontoclastic Tooth Resorption and Hypercementosis (EOTRH) syndrome. When features differed significantly (p<0.05).

Feature		EOTRH	0	1	2	3
**GLN**		Mean	15.0	13.5	15.5	15.8
		SD	4.3	5.6	6.9	6.8
	p	0.002	a	b	ab	a
**GLNN**		Mean	0.24	0.23	0.22	0.20
		SD	0.03	0.04	0.03	0.03
	p	< 0.0001	a	a	b	b
**GLV**		Mean	1.64	1.83	1.90	2.19
		SD	0.36	0.44	0.45	0.58
	p	< 0.0001	a	b	b	c
**HGLZE**		Mean	13.6	14.4	15.1	19.0
		SD	4.1	4.3	4.4	6.9
	p	< 0.0001	a	a	a	b
**LAE**		Mean	14354	14018	14372	12978
		SD	14574	13188	13972	12799
	p	0.6144	a	a	a	a
**LAHGLE**		Mean	183960	234333	252992	251620
		SD	206880	254514	298139	266459
	p	0.021	a	b	b	ab
**LALGLE**		Mean	1359	1013	1002	842
		SD	1293	941	914	972
	p	0.0007	a	b	ab	b
**LGLZE**		Mean	0.18	0.18	0.17	0.14
		SD	0.08	0.07	0.07	0.07
	p	0.0005	a	a	a	b
**SZN**		Mean	10.8	9.2	12.0	13.4
		SD	4.3	4.5	6.4	8.2
	p	< 0.0001	a	b	a	a
**SZNN**		Mean	0.17	0.16	0.16	0.16
		SD	0.05	0.04	0.04	0.05
	p	0.0614	a	a	a	a
**SAE**		Mean	0.39	0.37	0.39	0.39
		SD	0.08	0.07	0.07	0.08
	p	0.0569	a	a	a	a
**SAHGLE**		Mean	5.16	5.18	5.85	7.31
		SD	1.76	1.72	2.20	3.11
	p	< 0.0001	a	a	ab	b
**SALGLE**		Mean	0.08	0.07	0.07	0.06
		SD	0.05	0.04	0.03	0.03
	p	0.0076	a	a	ab	b
**ZE**		Mean	4.78	4.77	4.89	5.01
		SD	0.34	0.45	0.41	0.50
	p	0.0027	a	a	ab	b
**ZP**		Mean	0.0353	0.0351	0.0351	0.0440
		SD	0.0108	0.0103	0.0107	0.0247
	p	0.6555	a	a	a	a
**ZV**		Mean	13258	12960	13310	11955
		SD	13771	12629	13419	12049
	p	0.6217	a	a	a	a

GLN - gray level non-uniformity; GLNN - gray level non-uniformity normalized; GLV - gray level variance; HGLZE - high gray level zone emphasis; LAE - large area emphasis; LAHGLE - large area high gray level emphasis; LALGLE - large area low gray level emphasis; LGLZE - low gray level zone emphasis; SZN - size-zone non-uniformity; SZNN - size-zone non-uniformity normalized; SAE - small area emphasis; SAHGLE - small area high gray level emphasis; SALGLE - small area low gray level emphasis; ZE - zone entropy; ZP - zone percentage; ZV - zone variance. Lower case letters (a–c) indicate differences between classes for p < 0.05 independently for each feature.

Table S52. The values (mean ±SD) of features of *Gray Level Size Zone Matrix (GLSZM)* of output images, filtrated by *LaplacianSharpening* filter, compared between four classes (0-3) of the Equine Odontoclastic Tooth Resorption and Hypercementosis (EOTRH) syndrome. When features differed significantly (p<0.05).

Feature		EOTRH	0	1	2	3
**GLN**		Mean	65.6	64.5	77.0	71.2
		SD	23.4	33.5	43.8	25.8
	p	0.0016	a	a	b	b
**GLNN**		Mean	0.17	0.17	0.16	0.15
		SD	0.02	0.02	0.02	0.02
	p	< 0.0001	a	a	ab	b
**GLV**		Mean	2.85	2.93	3.08	3.75
		SD	0.51	0.49	0.55	1.28
	p	< 0.0001	a	ab	bc	c
**HGLZE**		Mean	33.2	35.9	37.7	45.8
		SD	8.8	10.9	10.7	12.9
	p	< 0.0001	a	ab	bc	c
**LAE**		Mean	494.8	399.7	406.0	435.9
		SD	613.2	374.0	438.9	474.5
	p	0.7564	a	a	a	a
**LAHGLE**		Mean	15283	15195	16204	18441
		SD	20751	17819	16483	16836
	p	0.2376	a	a	a	a
**LALGLE**		Mean	18.8	12.7	12.3	12.7
		SD	23.7	12.6	16.4	18.5
	p	0.0038	a	ab	ab	b
**LGLZE**		Mean	0.06	0.06	0.05	0.05
		SD	0.03	0.03	0.03	0.02
	p	< 0.0001	a	ab	ab	b
**SZN**		Mean	121.9	123.8	154.0	166.9
		SD	39.9	60.8	80.2	72.2
	p	< 0.0001	ab	a	bc	c
**SZNN**		Mean	0.31	0.33	0.33	0.34
		SD	0.03	0.04	0.03	0.04
	p	< 0.0001	a	b	b	c
**SAE**		Mean	0.58	0.59	0.59	0.60
		SD	0.03	0.04	0.03	0.04
	p	< 0.0001	a	b	ab	c
**SAHGLE**		Mean	19.1	21.0	22.2	27.6
		SD	5.1	6.3	6.2	7.9
	p	< 0.0001	a	ab	b	c
**SALGLE**		Mean	0.04	0.04	0.04	0.03
		SD	0.02	0.02	0.02	0.01
	p	0.0006	a	a	ab	b
**ZE**		Mean	5.02	4.93	5.01	5.04
		SD	0.21	0.29	0.24	0.22
	p	0.0037	a	b	ab	a
**ZP**		Mean	0.21	0.22	0.22	0.25
		SD	0.04	0.04	0.05	0.09
	p	0.0531	a	a	a	a
**ZV**		Mean	468.2	375.7	381.9	414.5
		SD	602.7	366.3	429.9	463.8
	p	0.7837	a	a	a	a

GLN - gray level non-uniformity; GLNN - gray level non-uniformity normalized; GLV - gray level variance; HGLZE - high gray level zone emphasis; LAE - large area emphasis; LAHGLE - large area high gray level emphasis; LALGLE - large area low gray level emphasis; LGLZE - low gray level zone emphasis; SZN - size-zone non-uniformity; SZNN - size-zone non-uniformity normalized; SAE - small area emphasis; SAHGLE - small area high gray level emphasis; SALGLE - small area low gray level emphasis; ZE - zone entropy; ZP - zone percentage; ZV - zone variance. Lower case letters (a–c) indicate differences between classes for p < 0.05 independently for each feature.

Table S53. The values (mean ±SD) of features of *Gray Level Size Zone Matrix (GLSZM)* of output images, filtrated by *DiscreteGaussian* filter, compared between four classes (0-3) of the Equine Odontoclastic Tooth Resorption and Hypercementosis (EOTRH) syndrome. When features differed significantly (p<0.05).

Feature		EOTRH	0	1	2	3
**GLN**		Mean	7.42	6.98	7.63	6.81
		SD	2.53	2.58	2.98	2.42
	p	0.1169	a	a	a	a
**GLNN**		Mean	0.26	0.26	0.24	0.22
		SD	0.04	0.05	0.04	0.05
	p	< 0.0001	a	a	a	b
**GLV**		Mean	1.52	1.78	1.79	2.16
		SD	0.40	0.50	0.49	0.66
	p	< 0.0001	a	b	b	c
**HGLZE**		Mean	10.7	11.2	11.6	14.8
		SD	3.4	3.6	3.7	5.7
	p	< 0.0001	a	a	a	b
**LAE**		Mean	38710	37112	41960	36943
		SD	28315	33309	37802	32694
	p	0.1575	a	a	a	a
**LAHGLE**		Mean	425904	547603	620720	644869
		SD	370465	615603	597496	578646
	p	0.0148	a	b	a	a
**LALGLE**		Mean	4539	3129	3540	2666
		SD	3300	2791	3318	2732
	p	< 0.0001	a	b	b	b
**LGLZE**		Mean	0.25	0.26	0.24	0.21
		SD	0.11	0.11	0.10	0.10
	p	0.0036	ab	a	ab	b
**SZN**		Mean	3.16	2.94	3.24	2.84
		SD	1.44	1.39	1.46	1.28
	p	0.1246	a	a	a	a
**SZNN**		Mean	0.11	0.11	0.10	0.09
		SD	0.04	0.04	0.03	0.03
	p	0.0093	a	a	ab	b
**SAE**		Mean	0.26	0.25	0.25	0.22
		SD	0.09	0.09	0.08	0.09
	p	0.0234	a	ab	ab	b
**SAHGLE**		Mean	2.35	2.14	2.38	2.60
		SD	1.21	1.17	1.23	1.66
	p	0.2281	a	a	a	a
**SALGLE**		Mean	0.08	0.09	0.08	0.06
		SD	0.06	0.06	0.05	0.05
	p	0.0001	a	a	a	b
**ZE**		Mean	4.31	4.30	4.45	4.55
		SD	0.43	0.48	0.46	0.48
	p	0.0004	a	a	ab	b
**ZP**		Mean	0.0156	0.0168	0.0158	0.0170
		SD	0.0045	0.0050	0.0051	0.0072
	p	0.121	a	a	a	a
**ZV**		Mean	33410	32368	36660	31410
		SD	25750	30584	34989	29090
	p	0.1644	a	a	a	a

GLN - gray level non-uniformity; GLNN - gray level non-uniformity normalized; GLV - gray level variance; HGLZE - high gray level zone emphasis; LAE - large area emphasis; LAHGLE - large area high gray level emphasis; LALGLE - large area low gray level emphasis; LGLZE - low gray level zone emphasis; SZN - size-zone non-uniformity; SZNN - size-zone non-uniformity normalized; SAE - small area emphasis; SAHGLE - small area high gray level emphasis; SALGLE - small area low gray level emphasis; ZE - zone entropy; ZP - zone percentage; ZV - zone variance. Lower case letters (a–c) indicate differences between classes for p < 0.05 independently for each feature.

Table S54. The values (mean ±SD) of features of *Gray Level Size Zone Matrix (GLSZM)* of output images, filtrated by *SmoothingRecursiveGaussian* filter, compared between four classes (0-3) of the Equine Odontoclastic Tooth Resorption and Hypercementosis (EOTRH) syndrome. When features differed significantly (p<0.05).

Feature		EOTRH	0	1	2	3
**GLN**		Mean	7.03	6.68	7.25	6.55
		SD	2.42	2.53	2.81	2.20
	p	0.2156	a	a	a	a
**GLNN**		Mean	0.26	0.26	0.25	0.23
		SD	0.05	0.06	0.04	0.06
	p	0.0004	a	a	a	b
**GLV**		Mean	1.52	1.78	1.78	2.14
		SD	0.42	0.51	0.52	0.68
	p	< 0.0001	a	b	b	c
**HGLZE**		Mean	10.5	10.9	11.3	14.5
		SD	3.6	3.4	3.8	6.0
	p	< 0.0001	a	a	a	b
**LAE**		Mean	41085	39862	45789	39009
		SD	29146	36290	42248	34781
	p	0.0966	a	a	a	a
**LAHGLE**		Mean	444818	583410	667443	676123
		SD	387918	663489	659453	613267
	p	0.0054	a	ab	b	b
**LALGLE**		Mean	5045	3388	4048	2849
		SD	3765	3076	3982	2966
	p	< 0.0001	a	b	b	b
**LGLZE**		Mean	0.26	0.26	0.25	0.21
		SD	0.12	0.11	0.11	0.10
	p	0.0017	a	a	a	b
**SZN**		Mean	2.98	2.71	2.93	2.56
		SD	1.44	1.24	1.34	1.10
	p	0.144	a	a	a	a
**SZNN**		Mean	0.11	0.11	0.10	0.09
		SD	0.04	0.04	0.03	0.03
	p	0.007	a	a	ab	b
**SAE**		Mean	0.25	0.24	0.23	0.21
		SD	0.10	0.10	0.09	0.09
	p	0.2691	a	a	a	a
**SAHGLE**		Mean	2.08	1.83	1.95	2.31
		SD	1.35	1.00	1.14	1.62
	p	0.3829	a	a	a	a
**SALGLE**		Mean	0.08	0.09	0.08	0.06
		SD	0.07	0.06	0.05	0.05
	p	0.0005	ab	a	ab	b
**ZE**		Mean	4.24	4.25	4.40	4.46
		SD	0.46	0.49	0.45	0.48
	p	0.002	ab	a	bc	c
**ZP**		Mean	0.0147	0.0160	0.0148	0.0159
		SD	0.0043	0.0050	0.0049	0.0061
	p	0.0569	a	a	a	a
**ZV**		Mean	35147	34515	39587	32916
		SD	26440	33052	38627	30537
	p	0.1199	a	a	a	a

GLN - gray level non-uniformity; GLNN - gray level non-uniformity normalized; GLV - gray level variance; HGLZE - high gray level zone emphasis; LAE - large area emphasis; LAHGLE - large area high gray level emphasis; LALGLE - large area low gray level emphasis; LGLZE - low gray level zone emphasis; SZN - size-zone non-uniformity; SZNN - size-zone non-uniformity normalized; SAE - small area emphasis; SAHGLE - small area high gray level emphasis; SALGLE - small area low gray level emphasis; ZE - zone entropy; ZP - zone percentage; ZV - zone variance. Lower case letters (a–c) indicate differences between classes for p < 0.05 independently for each feature.
